# RERconverge Expansion: Using Relative Evolutionary Rates to Study Complex Categorical Trait Evolution

**DOI:** 10.1093/molbev/msae210

**Published:** 2024-10-15

**Authors:** Ruby Redlich, Amanda Kowalczyk, Michael Tene, Heather H Sestili, Kathleen Foley, Elysia Saputra, Nathan Clark, Maria Chikina, Wynn K Meyer, Andreas R Pfenning

**Affiliations:** Department of Computational Biology, Carnegie Mellon University, Pittsburgh, PA 15213, USA; Department of Computational Biology, Carnegie Mellon University, Pittsburgh, PA 15213, USA; Neuroscience Institute, Carnegie Mellon University, Pittsburgh, PA 15213, USA; Department of Biological Sciences, Lehigh University, Bethlehem, PA 18015, USA; Department of Computational Biology, Carnegie Mellon University, Pittsburgh, PA 15213, USA; Department of Biological Sciences, Lehigh University, Bethlehem, PA 18015, USA; Department of Computational and Systems Biology, University of Pittsburgh, Pittsburgh, PA 15260, USA; Department of Computational and Systems Biology, University of Pittsburgh, Pittsburgh, PA 15260, USA; Department of Computational and Systems Biology, University of Pittsburgh, Pittsburgh, PA 15260, USA; Department of Biological Sciences, Lehigh University, Bethlehem, PA 18015, USA; Department of Computational Biology, Carnegie Mellon University, Pittsburgh, PA 15213, USA; Neuroscience Institute, Carnegie Mellon University, Pittsburgh, PA 15213, USA

**Keywords:** convergent evolution, evolutionary biology, genetics, diet

## Abstract

Comparative genomics approaches seek to associate molecular evolution with the evolution of phenotypes across a phylogeny. Many of these methods lack the ability to analyze non-ordinal categorical traits with more than two categories. To address this limitation, we introduce an expansion to RERconverge that associates shifts in evolutionary rates with the convergent evolution of categorical traits. The categorical RERconverge expansion includes methods for performing categorical ancestral state reconstruction, statistical tests for associating relative evolutionary rates with categorical variables, and a new method for performing phylogeny-aware permutations, “permulations”, on categorical traits. We demonstrate our new method on a three-category diet phenotype, and we compare its performance to binary RERconverge analyses and two existing methods for comparative genomic analyses of categorical traits: phylogenetic simulations and a phylogenetic signal based method. We present an analysis of how the categorical permulations scale with the number of species and the number of categories included in the analysis. Our results show that our new categorical method outperforms phylogenetic simulations at identifying genes and enriched pathways significantly associated with the diet phenotypes and that the categorical ancestral state reconstruction drives an improvement in our ability to capture diet-related enriched pathways compared to binary RERconverge when implemented without user input on phenotype evolution. The categorical expansion to RERconverge will provide a strong foundation for applying the comparative method to categorical traits on larger data sets with more species and more complex trait evolution than have previously been analyzed.

## Introduction

Determining the genomic elements underlying complex phenotypes is a fundamental and persistent challenge in biology. Despite decades of genomics research, the genetic underpinnings underlying most traits—ranging from lifespan and body size to habitat preference to sleep rhythms to type of body covering—remain elusive.

One way to address this challenge is through the lens of convergent evolution, the process in which multiple unrelated species independently acquire similar phenotypes. These independent evolutionary events are natural replicates of phenotype acquisition that can be used to study genomic changes—when similar genetic changes correspond with similar phenotypic changes, that may indicate a genotype–phenotype relationship (Li et al. 2010; Partha et al. 2017; Kowalczyk et al. 2019).

Genetic convergence can be quantified by measuring convergent evolution at the same individual nucleotides (Hasselmann et al. 2008), individual amino acids (Thomas and Hahn 2015; Rey et al. 2018; Fukushima and Pollock 2023), or through evolutionary rates summarized over a length of sequence, such as a gene (Kowalczyk et al. 2019; Kosakovsky Pond et al. 2020) or regulatory element (Partha et al. 2017; Hu et al. 2019; Kowalczyk et al. 2022; Kaplow et al. 2023; Yan et al. 2023). Evolutionary rates, measured as rate of substitutions along the branches of a phylogeny, change due to selective pressures such as increased evolutionary constraint, relaxation of evolutionary constraint, or positive selection (Pond et al. 2005; Yang 2007). Genetic convergence methods have been applied to identify convergent molecular evolution associated with a wide variety of traits in a large number of clades (e.g. Wang et al. 2020; Espindola-Hernandez et al. 2022; Jin et al. 2023; Yusuf et al. 2023; Bodawatta et al. 2023).

However, a limitation to most phylogenetic comparative methods is their inability to analyze non-ordinal, categorical traits with more than two categories. For example, the mammalian diet type phenotype has three main categories (herbivore, omnivore, and carnivore) that cannot be meaningfully ordered. To study such a phenotype using most existing methods would require coercing the phenotype into binary groupings, which is not straightforward (should we compare herbivore to omnivore and then compare herbivore to carnivore, or should we compare herbivore to all non-herbivores as one group?).

One method that does specifically study categorical data, here referred to as “phylANOVA”, uses an ANOVA test to compare means of a continuous phenotype for extant species across categories and computes *P*-values empirically from simulations to account for phylogenetic relationships between species (Garland et al. 1993). However, phylANOVA is limited by the strict assumptions of the ANOVA test, which are often violated by genomic data like evolutionary rates.

More recently, Ribeiro and Borges presented the delta statistic for calculating phylogenetic signal for categorical traits (Borges et al. 2019; Ribeiro et al. 2023). One of many applications of the delta statistic is identifying genetic elements associated with a categorical phenotype. This statistic has been successfully applied to find genes associated with the evolution of mammalian sleep patterns (Borges et al. 2019). However, the delta statistic cannot be applied in the specific context of finding genetic elements that are convergently accelerated or conserved as it does not provide information on evolutionary rates, and it lacks a method for computing *P*-values that takes phylogenetic relationships into account.

To address the current limitations of phylogenetic comparative methods for categorical trait analysis, we introduce categorical methods within RERconverge, an R package for the genome-wide association of convergent evolutionary rate shifts with convergent phenotypes (Kowalczyk et al. 2019). The rate of substitution along a branch is quantified as an evolutionary rate and, by correcting for factors affecting rates at all genes or on all branches of a phylogeny, we can compute relative evolutionary rates (RERs) that reflect whether a given gene is evolving slower or faster than expected along a branch of the phylogeny (Kowalczyk et al. 2019). Comparing the distributions of these RERs across phenotypes enables us to identify regions of the genome showing convergent rate shifts in association with the phenotype, thus making use of convergent evolution as a tool in comparative genomic analyses.

Unlike many phylogenetic software packages, RERconverge can also infer the ancestral history of a trait to use when associating evolutionary rates with the evolution of the trait. By using inferences about the phenotypes of ancestors of extant species, the method is able to detect even ancient rate shifts associated with phenotype changes. The package also provides a built-in method, known as permulations, for the correction of *P*-values for phylogenetic dependence and other sources of systematic variation leading to nonindependence in the data (Saputra et al. 2021). RERconverge has been used with great success to discover both coding and noncoding elements related to the evolution of continuous and binary traits including mammalian lifespan, hairlessness, and marine habitation (Chikina et al. 2016; Kowalczyk et al. 2020, 2022).

The extension to RERconverge presented here provides three updates enabling the use of the software for analyses of categorical traits: (i) it infers the ancestral evolutionary history of categorical traits; (ii) it provides a permulation strategy for categorical traits to return reliable, phylogeny aware, corrected *P*-values; and (iii) it has options for both parametric and nonparametric statistical tests to associate RERs with categorical phenotypes, including post hoc pairwise testing. As a test case, we applied these new methods to a set of categories representing variation in mammalian diet phenotypes. One common way of categorizing mammalian diets is by trophic level: carnivory, herbivory, and omnivory (Kim et al. 2016). Diet can be further divided into smaller categories including, for example, insectivores and granivores (Eisenberg 1981). Applying our new methodology, we found multiple diet-related pathways enriched for genes with significant differences in RER distributions across diet categories, highlighting the potential of this method for genome-wide analyses.

## Results

### Diet Phenotype Ancestral Reconstruction

To extend RERconverge to enable analyses of categorical traits, we must be able to infer the ancestral history of categorical traits in order to associate phenotypic state with RERs on both terminal and internal branches of the phylogeny. To do so, we implemented maximum likelihood ancestral trait reconstruction using a continuous time Markov model of evolution; a framework that has been commonly applied to categorical traits (Pagel 1994; Pupko et al. 2000; Paradis et al. 2004). Previous RERconverge ancestral trait reconstruction functionality was only available for binary or continuous traits and used an approximate maximum parsimony approach for binary traits and estimation of maximum likelihood states for continuous traits (Maddison 1989; Kowalczyk et al. 2019; Revell 2024).

**Fig. 1.**
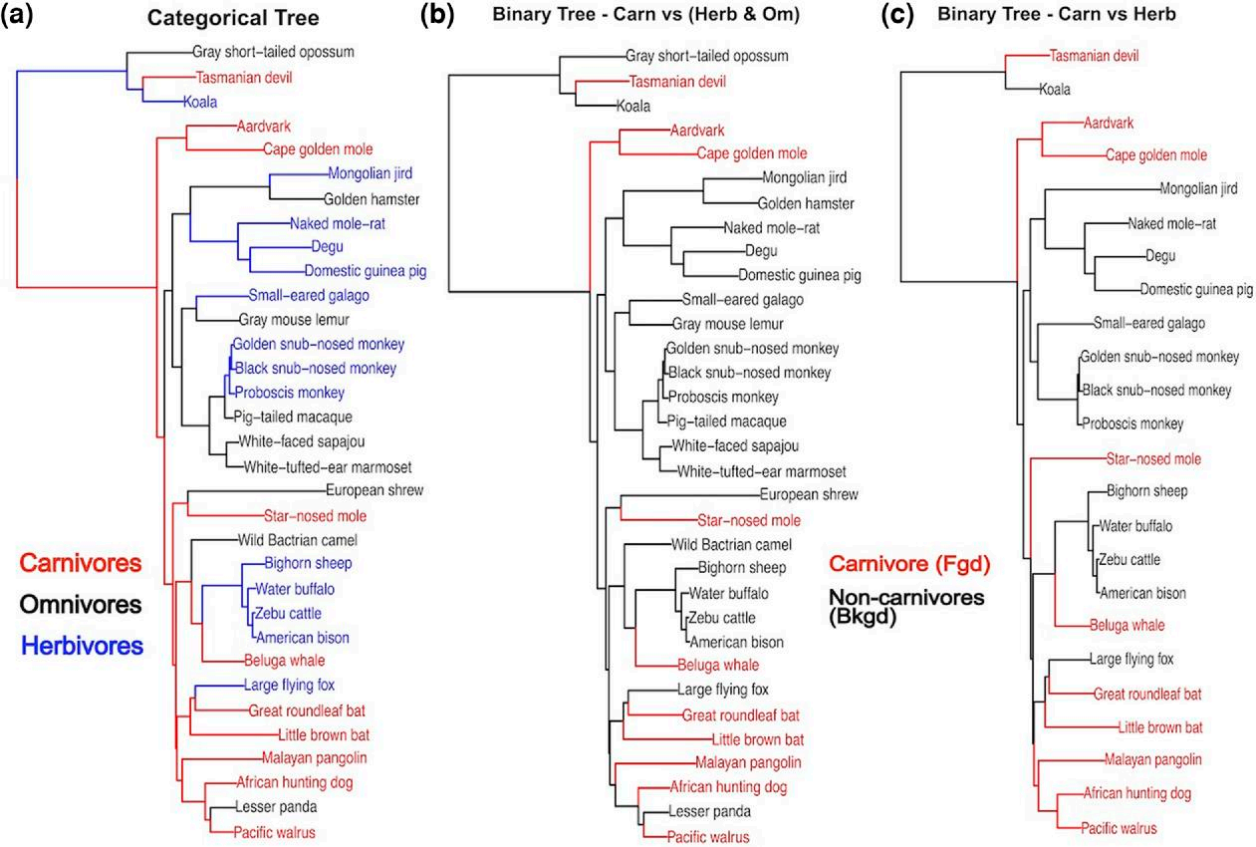
Example of a categorical trait reconstruction on a subset of mammals included in the full analyses (full trait reconstructions supplementary fig. S1, Supplementary Material online). a) Categorical trait reconstruction using maximum likelihood applied to a continuous time Markov model. b) Default binary trait reconstruction with carnivore foreground and herbivore/omnivore background. Uses an approximate maximum parsimony based approach and assumes trait evolution can only occur from background to foreground. c) Default binary reconstruction on phylogeny of carnivores (foreground) and herbivores (background) with omnivores removed. Uses an approximate maximum parsimony based approach and assumes trait evolution can only occur from background to foreground.

To test our extension of the RERconverge method to categorical traits, we obtained non-ordinal categorical phenotype data and a mammalian phylogeny along which to infer ancestral phenotype states. We used the diet phenotype (specifically trophic level: carnivore, omnivore, and herbivore) for 115 mammals, along with gene trees inferred from coding sequence alignments from these species’ high quality genomes (Hecker and Hiller 2020). The diet phenotype was annotated using prior literature for those species (Nowak 1999) (Fig. 1, supplementary fig. S1, Supplementary Material online, supplementary file S1, Supplementary Material online (main analysis phenotype vector)).

In the Markov model framework, the user places constraints on the rates inferred in the transition rate matrix of the Markov model through a rate model (Paradis et al. 2004) (supplementary fig. S2, Supplementary Material online).

Using a likelihood ratio test, as implemented in the expanded categorical RERconverge (Pagel 1994; Kowalczyk et al. 2019), we selected the all rates different (ARD) model to infer the ancestral states (supplementary fig. S2, Supplementary Material online) (supplementary Results, Supplementary Material online).

Under the ARD model, the ancestral mammal was inferred to be a carnivore, while omnivores and herbivores were inferred to have evolved independently multiple times, suggesting the presence of real phenotypic convergence that could be used to identify diet-associated convergent molecular evolution. Specifically, this inference included 4 direct transitions between carnivore and herbivore, 12 direct transitions between carnivore and omnivore, and 19 direct transitions between herbivore and omnivore, where each transition is interpreted as a potential independent convergent event (supplementary fig. S1a, Supplementary Material online, supplementary data S1, Supplementary Material online).

### Methods Comparison

To compare our new categorical RERconverge method to existing methods that handle categorical phenotypes, we performed a phylogenetic simulations “phylANOVA” analysis and a delta statistic analysis (Borges et al. 2019). We also performed two types of binary RERconverge pairwise analyses (Kowalczyk et al. 2019) with different strategies of defining the binary foreground/background split to assess how a method designed for categorical data compares to artificially binarizing a multi-categorical trait (Table 1, supplementary table S1, Supplementary Material online).

**Table 1:** Comparison of the methods

Method	Ancestral reconstruction	Comparisons	*P*-values	Approximate runtime (min)	
				Main analysis^a^ (% of total time spent computing RERs)	Permulations^b^
Categorical RERconverge	Maximum likelihood applied to CTMM	Categorical with post hoc pairwise tests	Parametric, permulations	70.15 (99.8%)	86
Pairwise binary RERconverge (strategy I)	Maximum parsimony	Binarized	Parametric, permulations	70.15 (99.8%)	∼210^c^
Pairwise binary RERconverge (strategy II)	Maximum parsimony	Binarized	Parametric, permulations	120.75 (99.4%)	∼360^d^
PhylANOVA^e^	None	Categorical with post hoc pairwise tests	Simulations	184 (38%)	ND
Delta statistic	Ancestral probability vectors	Categorical	ND	103	ND

^a^Runtime is for 19,137 genes on a 2021 MacBook Pro with an Apple M1 Max chip. Does not include the time to perform a pathway enrichment analysis. Does not include the time to estimate trees from the multiple sequence alignment since this is universal to all the methods. The runtime for pairwise binary RERconverge strategy I was determined using all 115 species with herbivores as foreground and the runtime for pairwise binary RERconverge strategy II was determined using carnivores/herbivores with carnivore foreground (total of 85 species).

^b^Time to run 500 permulations. For this paper, we ran 10,000 permulations and that runtimes for permulations can vary depending on the specific structure of the phenotype.

^c^The runtime was 70 min for the binary phenotype with herbivore foreground. To estimate the total runtime for all three pairwise tests (one for each category as foreground), this was multiplied by 3 to obtain ∼210 min. This is approximate since the runtime of permulations varies depending on the structure of the phenotype.

^d^The runtime was 60 min for the binary phenotype with carnivore foreground and herbivore background (omnivores removed). To estimate the total runtime for all six pairwise tests, this was multiplied by 6 to obtain ∼360 min. This is approximate since the runtime of permulations varies depending on the structure of the phenotype.

^e^The base RERconverge package was used to estimate relative evolutionary rates; those for terminal branches were then associated with the phenotype data for extant species using phylANOVA. Runtime includes time to perform 500 simulations (for this paper, we ran 10,000 simulations). This is included in the main analysis runtime since they are performed by default, rather than being an additional workflow like permulations.

In the following methods comparison, we will refer to the categorical RERconverge statistical test which directly compares all categories as the “omnibus test”, similar to the omnibus test from a traditional ANOVA analysis. Our new categorical method also performs pairwise post hoc testing, which we will refer to as a categorical post hoc test along with the two categories being compared. Post hoc testing compares each pair of traits directly (for example omnivore vs. carnivore) while the omnibus test assesses signal across all traits simultaneously (supplementary table S1, Supplementary Material online).

The phylogenetic simulations analysis uses the implementation from the phytools package, phylANOVA, with the terminal branch RERs computed by RERconverge and assigned to extant species as the continuous variable. This analysis will simply be referred to as phylANOVA. The delta statistic of phylogenetic signal will be referred to as the delta statistic.

To compare our multi-categorical method to binary RERconverge, we used two different strategies to binarize the diet phenotype. In the first type of binary analyses, we chose one state as the foreground and allowed the other two states to form the background without removing species. We will refer to these analyses in terms of the foreground state (e.g. carnivore/non-carnivore). In the second type of analyses, we removed all species of the third state. We will refer to these analyses in terms of the two states being compared in the order foreground/background (e.g. carnivore/herbivore when the foreground state is carnivore and herbivore/carnivore when the foreground state is herbivore) (supplementary table S1, Supplementary Material online).

All of these methods were tested on the same set of 19,137 gene trees based on the reference phylogeny and alignment of 115 mammals (Hecker and Hiller 2020) whose phenotypes we could clearly assign (Nowak 1999). In comparing the different RERconverge methods, we focus on the ancestral reconstruction approach and whether the trait is modeled categorically or binarized (Table 1). To assess categorical RERconverge in comparison to the other categorical methods, phylANOVA and the delta statistic, we focus on key methodological differences including ancestral reconstruction, how *P*-values are computed, and the types of signal detected (Table 1).

#### Overview of Methods Compared

**Fig. 2.**
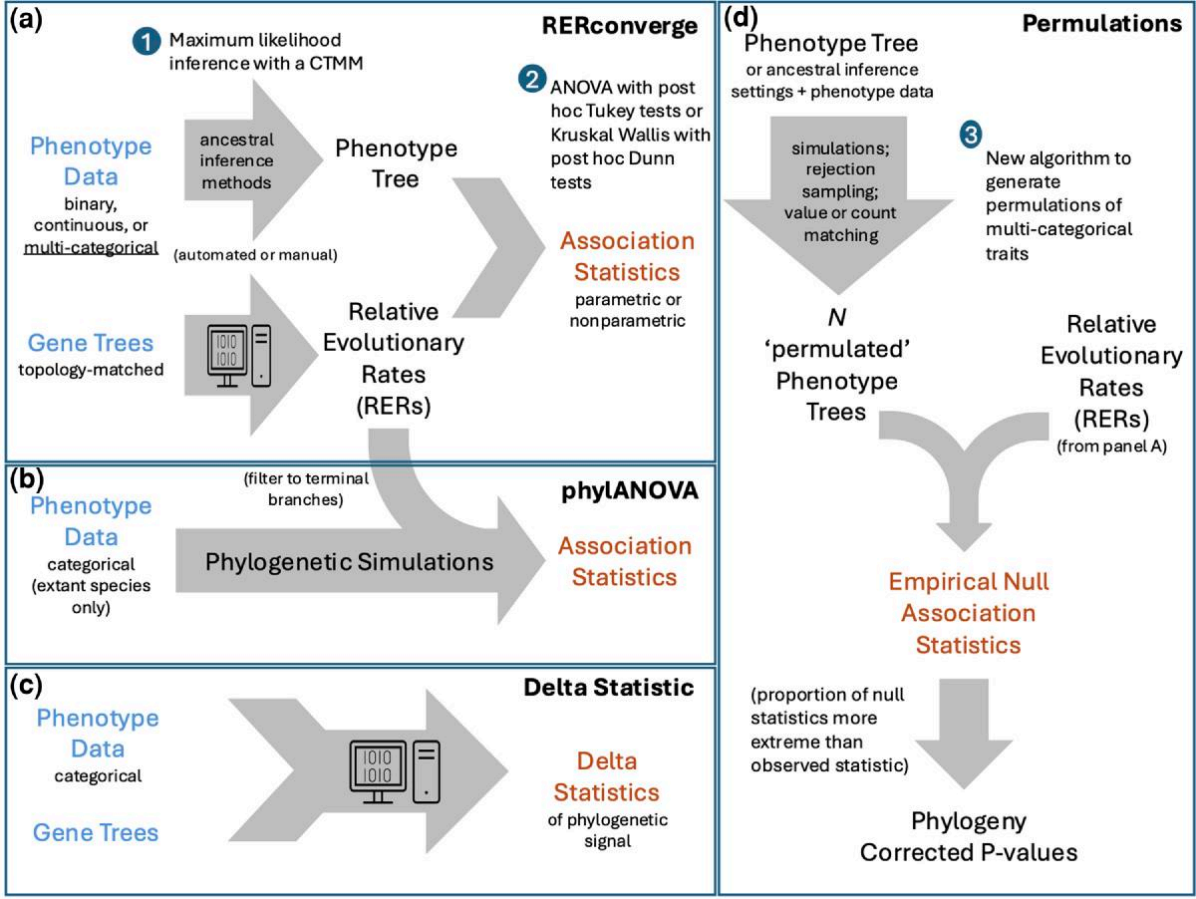
Flow chart of each method included in the methods comparison. a) Flow chart of RERconverge for binary, continuous, or categorical traits. b) Flow chart of phylANOVA using the RERs computed from RERconverge. c) Flow chart of the delta statistic of phylogenetic signal. d) Flow chart of “permulations”. In panels a) and d), numbers 1, 2, and 3 indicate the changes implemented in our update to process/analyze categorical traits.

One of the key methodological differences, the ancestral reconstruction approach, refers to how the different methods use or assign phenotypes to/on internal branches. PhylANOVA only includes extant species and therefore does not assign ancestral states (Table 1, Fig. 2). By default, binary RERconverge uses an approximate maximum parsimony approach to infer ancestral states and we chose for categorical RERconverge to use maximum likelihood with a continuous time Markov model (CTMM) to infer ancestral states (Pagel 1994; Pupko et al. 2000; Paradis et al. 2004) (Table 1, Fig. 2). We note that RERconverge also allows users to manually specify a binary or categorical phenotype tree, including providing a graphical user interface for selecting branches. This approach may be preferred when the default inference is not consistent with other sources of information such as fossil evidence. However, we chose to compare the behavior of the default inference methods because they can be used for analyses in which only data for extant species are available.

Another key methodological difference is how *P*-values are calculated (Table 1). Data in a phylogenetic context always violate the assumption of traditional statistical testing that each observation is independent because the data are related to each other based on their shared evolutionary history represented by their phylogeny. Therefore, to calculate meaningful *P*-values, it is helpful to construct an empirical null distribution by generating many null instances and calculating the *P*-value as the proportion of the null instances that have a more extreme statistic than observed using real data. The methods presented here use different strategies to create these null instances. PhylANOVA uses a simulation approach to create null values (in our case, null terminal branch RERs), a procedure that creates new values based on inferred evolution along the phylogeny. Simulations have the advantage of generating values with respect to the phylogeny, but they have the limitation that simulated values may have a very different distribution from that of original values. RERconverge uses permulations, which generate null phenotype values using simulations restricted by the observed phenotype distribution to overcome that limitation of simulations. The delta statistic uses permutations of the phenotype to generate null instances. While permuted phenotypes represent the null hypothesis of no phylogenetic signal, they do not represent the null hypothesis that a gene is not involved in the evolution of a phenotype since we expect even null phenotypes to show some phylogenetic signal (Saputra et al. 2021), so here we assess the delta statistics using its statistic alone.

#### Comparison of Categorical RERconverge to Pairwise Binary RERconverge: Influence of Ancestral State Reconstruction and Removing Species

Before comparing categorical RERconverge to other methods that handle categorical data, we first compare to the existing binary RERconverge method to assess the impact of the CTMM ancestral reconstruction and the effect of removing species during trait binarization. Which strategy is used to coerce the multi-categorical diet trait into a binary trait has different consequences on the default binary ancestral reconstruction. In particular, the choice to remove a category of species from the analysis or to assign it to the background will have a different impact on the ancestral inference as illustrated in our toy example by the different states assigned to the common ancestor of the Pacific walrus and African hunting dog (Fig. 1b and c). Coercion to a binary trait has additional consequences on the calculation of relative evolutionary rates if species are removed.

**Fig. 3.**
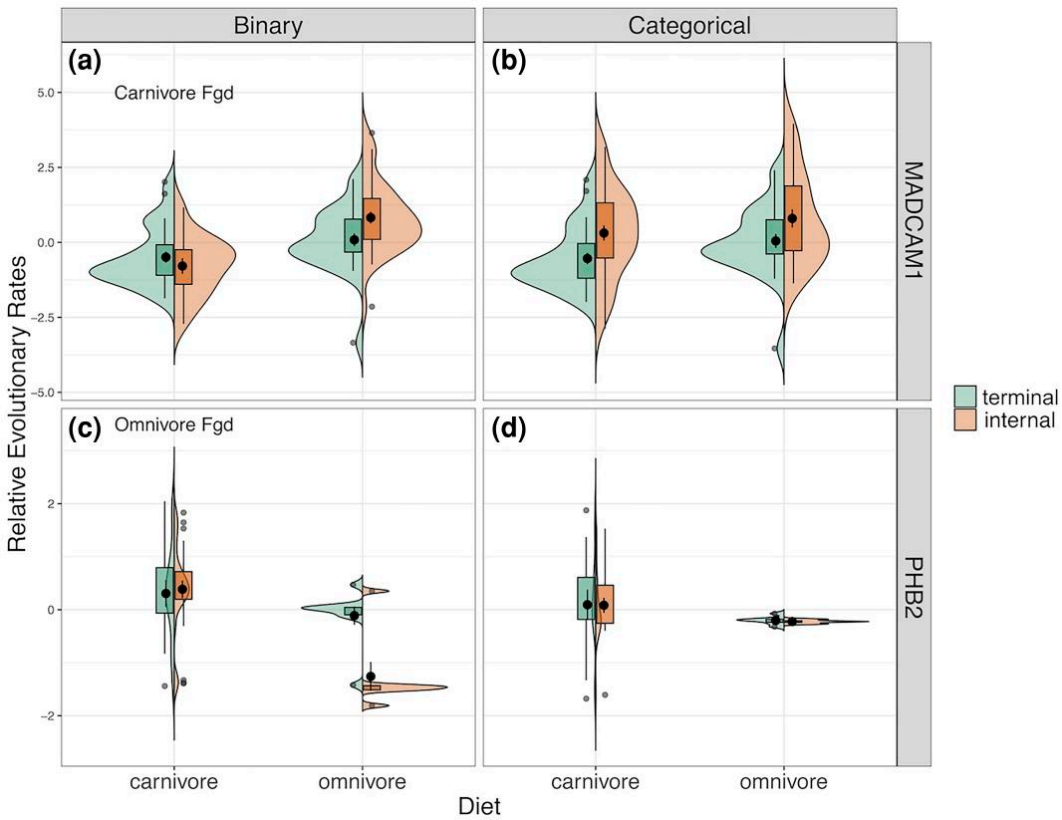
Each violin plot compares the distribution of RERs between inferred carnivore and omnivore branches for MADCAM1 (top row) and PHB2 (bottom row). The distribution on the left is the distribution of RERs for terminal branches and the distribution on the right is the distribution of RERs for internal branches. The left column shows the RERs from the binary analyses of carnivores versus omnivores with either carnivore foreground (for MADCAM1) or omnivore foreground (for PHB2). The right column shows the RERs from the categorical analysis.

Given these consequences, we wanted to investigate how such differences in internal branch assignments and RER calculation impact the gene association statistics produced by RERconverge. We focused on the carnivore/omnivore and omnivore/carnivore binary analyses because their distributions of permulation *P*-values differed the most from that of the corresponding categorical post hoc test (supplementary fig. S4b, Supplementary Material online), suggesting that something such as differences in internal branch assignment could be driving the difference in the apparent signal of these methods. We then selected two genes that each had a significant (large effect) rate shift in one of these binary comparisons but did not show a significant rate shift according to the corresponding categorical post hoc test. In both genes, whether a rate shift is observed or not is driven by differences in the RER distributions of the internal branches (Fig. 3).

MADCAM1 is significantly accelerated in omnivores according to the carnivore/omnivore binary analysis (permulation *P* = 0.0067), however this is not the case in the corresponding categorical post hoc test (permulation *P* = 0.2161). Separating the relative evolutionary rates by terminal versus internal branches, we see that in the categorical method, the RERs of carnivores are driven upwards due to the presence of internal branches with large RERs assigned as carnivores; some of these branches were likely assigned as omnivores or excluded in the binary analysis (Fig. 3b). This leads to no significant difference between the distributions of RERs in carnivores and omnivores in the categorical post hoc test.

PHB2 is significantly accelerated in carnivores according to the omnivore/carnivore binary analysis (permulation *P* < 1 × 10^−4^) but not in the categorical post hoc test (permulation *P* = 0.0776). The signal for this gene in the binary analysis is being driven by a group of internal branches assigned as omnivores with negative RERs (Fig. 3c). There is no corresponding group of internal branches with negative RERs assigned as omnivores—or any other category—in the categorical reconstruction, likely because removing all herbivores affects the computation of RERs. When this group of internal branches with negative RERs is no longer present, there is no significant difference in RERs between carnivores and omnivores (Fig. 3d). Though the true ancestral phenotypes and evolutionary rates may remain unknown, these results suggest that the categorical method may help to control for false positives.

**Fig. 4.**
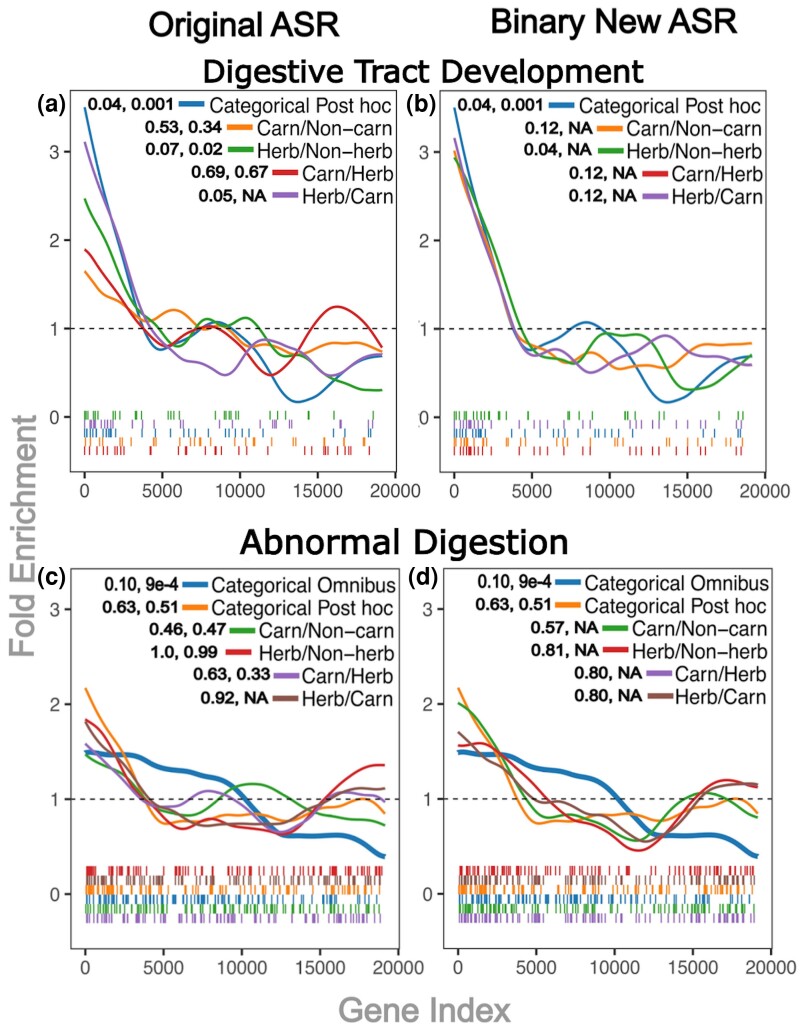
Fold enrichment and barcode plots for the abnormal digestion and digestive tract development pathways, respectively. *P*-values are reported as FDR (Benjamini–Hochberg), permulation *P*-value. For tests where permulations were not performed, the permulation *P*-value is reported as NA. a) The categorical carnivore/herbivore post hoc test is compared to the four different original binary analyses which compared carnivores to herbivores. b) The categorical carnivore/herbivore post hoc test is compared to the four binary RERconverge analyses in which the new ancestral state reconstruction (ASR) method has been used to assign phenotypes to branches. c) The categorical omnibus test and the categorical carnivore/herbivore post hoc test are compared to the four different original binary analyses which compared carnivores to herbivores using the default method for phenotype inference. d) The categorical omnibus test and the categorical carnivore/herbivore post hoc test are compared to the four binary RERconverge analyses in which the new ASR method, maximum likelihood applied to a continuous time Markov model, has been used to assign phenotypes to branches.

Effects of the ancestral reconstruction extend beyond the gene level results to the pathway enrichment results as well. One GO pathway for which we may expect to see significant enrichment is digestive tract development (Subramanian et al. 2005; Liberzon et al. 2011). Carnivores have been shown to have shortened digestive tracts relative to non-carnivores (Stevens and Hume 1995), and we would expect the digestive tract to specialize to a species’ diet. Digestive tract development was significantly enriched in the carnivore/herbivore categorical post hoc test (permulation *P* = 0.0011), while enrichment is lower and *P*-values higher among the carnivore/non-carnivore, herbivore/non-herbivore, carnivore/herbivore, and herbivore/carnivore binary pairwise analyses (Fig. 4a).

To test the effect of the ancestral reconstruction method relative to potentially different relative evolutionary rate values (in binary analyses where species were removed) and the different statistical tests employed between the categorical post hoc and binary pairwise methods, we used the CTMM ancestral reconstruction method to infer the ancestral states for each of the four sets of binary phenotypes. We then performed the rest of the binary analysis unchanged (Fig. 4b). Note that the two binary methods in which omnivores were removed (red and purple lines) now had the same assignment of ancestral states because the reconstruction is no longer dependent on foreground choice. Using the new ancestral reconstruction caused the enrichment of digestive tract development genes to increase in all of the binary tests except for the herbivore/carnivore binary analysis such that their fold enrichment curves more closely matched that of the categorical post hoc test (Fig. 4a and b). In those tests with increased fold enrichment, the Benjamini–Hochberg corrected *P*-values decreased as well. Thus, we infer the differences in enrichment were being driven almost entirely by the ancestral reconstruction. Using the CTMM for ancestral reconstruction resulted in increased enrichment for a pathway with clear relevance to the diet phenotype, suggesting that capturing more complex patterns of evolution and using more well-supported reconstructions can improve power at the pathway enrichment level as well.

#### Comparison of Categorical RERconverge to Pairwise Binary RERconverge: Influence of Including Multiple Categories

Beyond avoiding the direct impacts of binarization on ancestral state reconstruction and species removal, the addition of categorical statistical tests in categorical RERconverge allows for the comparison of RERs of multiple phenotype categories simultaneously. This allows the method to detect rate shifts when either all or a subset of categories have those shifts (see supplementary Categorical RERconverge Results, Supplementary Material online). We demonstrate that the categorical omnibus test can identify an enriched pathway of relevance that is not identified by any one binary or categorical pairwise/post hoc comparison, regardless of the method of ancestral reconstruction.

Similar to digestive tract development, we might expect the MGI pathway, abnormal digestion, to be associated with diet type (Subramanian et al. 2005; Liberzon et al. 2011). We found significant enrichment of genes within this pathway in the categorical omnibus test but not in the categorical post hoc or pairwise binary analyses (Fig. 4c and d, supplementary fig. S5, Supplementary Material online; omnibus permulation *P* = 9 × 10^−4^ vs. all categorical post hoc permulation *P* ≥ 0.27, all pairwise binary permulation *P* ≥ 0.10). The lack of enrichment for the pairwise binary analyses remained even when performed with the CTMM ancestral reconstruction as was done with the digestive tract development pathway (Fig. 4c and d, supplementary fig. S5, Supplementary Material online). This suggests that the categorical omnibus test is capturing rate shifts spread out across multiple pairwise comparisons which alone aren’t sufficient to detect enrichment for this pathway.

**Fig. 5.**
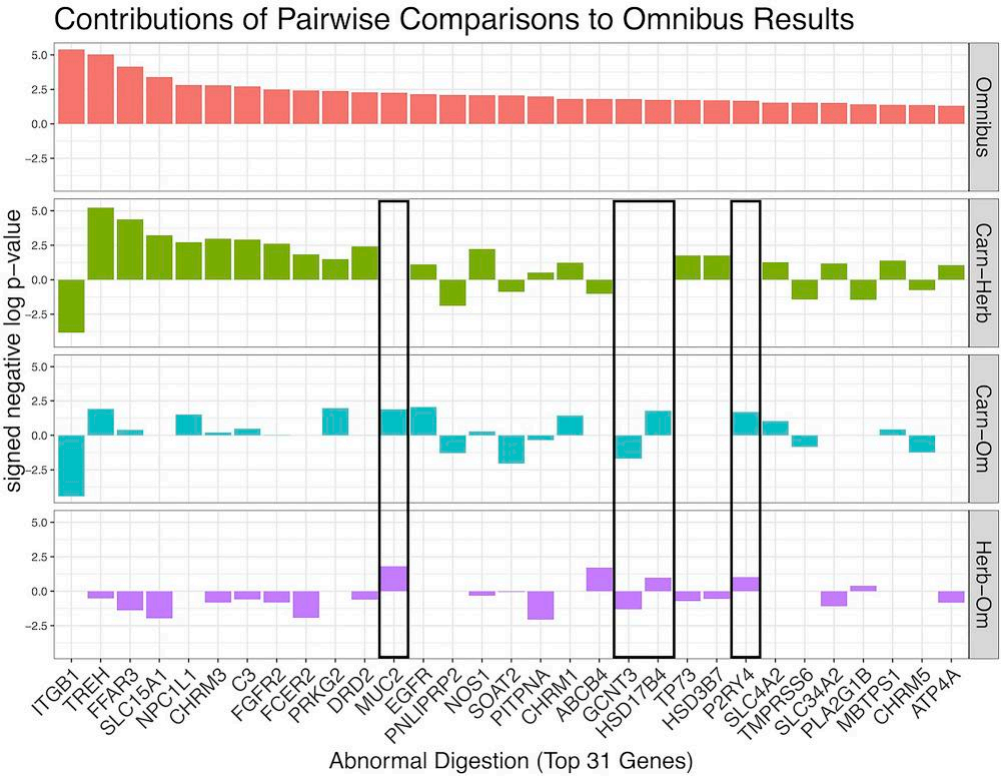
Bar plots of the top 31 genes in the abnormal digestion pathway (as ranked by the omnibus results) for the categorical omnibus and pairwise tests. The height of the bar represents the signed negative log *P*-value.

To interpret the improved performance of the omnibus statistic, we next explored the behaviors of the 31 genes in the abnormal digestion pathway with an uncorrected *P*-value from the categorical omnibus test less than 0.05 (supplementary data S2, Supplementary Material online). There are two important factors which may be contributing to the stronger enrichment under the omnibus test compared to the pairwise tests. First, some genes within this pathway exhibit significant rate shifts in opposite directions under the same post hoc comparison(s). For instance, ITGB1 is evolving slower in carnivores compared to herbivores and omnivores, whereas TREH is evolving faster in carnivores compared to herbivores and omnivores (Fig. 5). Binary RERconverge determines enrichment using a Wilcoxon rank sum test to identify whether genes within a pathway are together shifted toward accelerated evolutionary rates or toward slower evolutionary rates in foreground lineages. As a result, genes within the same pathway with evolutionary rate shifts in opposite directions with regard to the phenotype do not both contribute to the same enrichment signal and may even “cancel out” each other's effect. The omnibus test is one-sided, so high ranking genes within the same pathway contribute to gene set enrichment, regardless of the direction of the rate shift for each gene. Second, significant differences in evolutionary rates for genes in this pathway arise among all three pairwise comparisons, not just one. Though many of the top genes in this pathway have rate shifts in carnivores relative to herbivores, there are multiple top genes within the abnormal digestion pathway whose individual signal is being driven by differences in evolutionary rates between carnivores/omnivores and/or herbivores/omnivores instead (Fig. 5, indicated by black boxes). The observation that these comparisons contribute to the enrichment of abnormal digestion genes (Fig. 5) emphasizes that important information would be lost by excluding them.

Together, the digestive tract development and abnormal digestion pathways demonstrate that the enrichment signal of a pathway may be driven by a single pairwise comparison or by multiple pairwise comparisons. Whether more than one pairwise comparison is contributing to enrichment may depend on which genes are experiencing changes in selective pressure in each phenotype state and on the statistical power of each pairwise test. In the case of enrichment being driven by a single pairwise comparison, as with digestive tract development, we would not necessarily expect an omnibus test to improve the strength of the enrichment. Alternatively, when multiple pairwise comparisons are driving the enrichment, we expect that using a categorical omnibus test will improve our power to detect the pathway, and indeed this was the case with the abnormal digestion pathway.

#### Comparison to PhylANOVA

The abnormal digestion pathway demonstrates that using a categorical omnibus test has advantages over pairwise binary comparisons alone. A simple way to perform an omnibus test on RERs might be to compare the terminal branch RERs returned by RERconverge across phenotype categories using phylANOVA (Table 1, Fig. 2). However, we show that phylANOVA vastly underperforms compared to our categorical RERconverge. Comparing the simulation *P*-values to the permulation *P*-values using quantile–quantile plots (supplementary figs. S3 and S4, Supplementary Material online gray vs. red) shows that phylANOVA has much lower power to detect genes with convergent shifts in evolutionary rates than categorical RERconverge when the same number (10,000) simulations/permulations are performed. For example, phylANOVA detects 142 genes with *P* < 0.05, whereas categorical RERconverge detects 1086 genes with *P* < 0.05 with the omnibus test. To highlight that this global difference in power was related to the power to detect diet-specific signal, and not just a difference in false positive rate, we demonstrated that phylANOVA was less effective compared to binary and categorical RERconverge at detecting diet-relevant enriched pathways; for example, 0% of significantly enriched MGI pathways in the carnivore/herbivore post hoc phylANOVA results were related to metabolism or the digestive, liver, or biliary systems, in comparison to 8% to 23% of the carnivore/herbivore binary or categorical RERconverge results (supplementary fig. S7, Supplementary Material online, supplementary phylANOVA Comparison Results, Supplementary Material online).

#### Comparison to the Delta Statistic

**Fig. 6.**
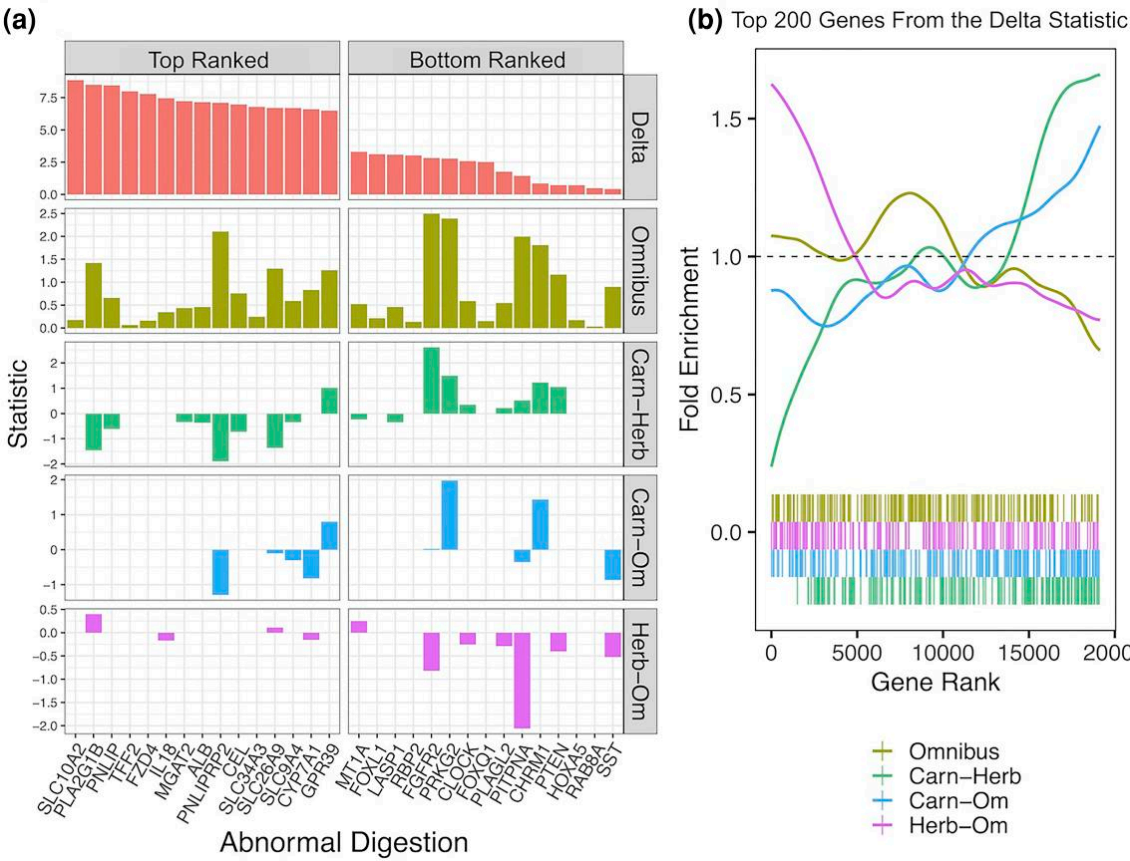
Different abnormal digestion genes drive results for the delta statistic and categorical RERconverge. a) Bar plots of the statistic used to calculate enrichments for the top 15 genes in the abnormal digestion pathway (right) and the bottom 15 genes in the abnormal digestion pathway (left), as ranked by the delta statistic. For the delta statistic method, the statistic is delta, the measure of phylogenetic signal. For the RERconverge methods, the statistic is the signed log *P*-value. b) The fold enrichment and barcode plots among the RERconverge omnibus and post hoc tests of the top 200 genes ranked by the delta statistic of phylogenetic signal.

Next we compare categorical RERconverge to another method, the delta statistic, that also seeks to address the lack of available comparative tools to study categorical traits. Although, unlike the other methods in this analysis, the delta statistic does not use evolutionary rates, we still expect it to identify genes associated with the convergent diet phenotypes based on molecular evolutionary patterns. More details on how the delta statistic is calculated are given in the delta statistic paper (Borges et al. 2019; Ribeiro et al. 2023) and summarized in the supplementary Methods, Supplementary Material online. In order to determine if the delta statistic and categorical RERconverge identified similar or different signatures of convergent evolution, we first compared both methods on the abnormal digestion pathway, which was significantly enriched according to both. The genes in the abnormal digestion pathway with the largest effect sizes identified by the delta statistic did not correspond with the most significant genes in this pathway identified by omnibus categorical RERconverge (Fig. 6a). Although there is no clear relationship between the delta statistic and the omnibus results, patterns emerge when the results are broken down into their pairwise comparisons. The largest delta statistic genes tended to be those which were evolving slower in carnivores compared to omnivores or herbivores, as indicated by negative test statistics for post hoc RERconverge (Fig. 6a). Genes in the pathway which were ranked lowest according to the delta statistic, but which exhibited a rate shift, tended to be those evolving faster in carnivores compared to herbivores or omnivores (Fig. 6a).

To determine if this trend persists across all genes in the analysis, not just those within the abnormal digestion pathway, we plotted the fold enrichment of the top 200 delta statistic genes among the categorical RERconverge tests (Fig. 6b). In the fold enrichment plot, gene rank is from lowest to highest *P*-value for the omnibus test. Since the post hoc tests are two-sided, genes were ranked from lowest *P*-values with positive effect sizes to lowest *P*-values with negative effect sizes, such that genes with particularly high or low ranks correspond to genes with large rate shifts in either the positive or negative direction. The overall results indeed recapitulate what we observed within the abnormal digestion pathway. Genes with greater phylogenetic signal for the diet phenotype tend to evolve slower in carnivores compared to herbivores (Fig. 6b, green). To a lesser extent, the same trend is observed for carnivores compared to omnivores (Fig. 6b, blue) and omnivores compared to herbivores (Fig. 6b, pink). There is no enrichment of the top delta statistic genes among the omnibus results (Fig. 6b, yellow), indicating that overall the two methods are identifying different, though potentially complementary, top genes associated with this diet phenotype.

### Example of Downstream Analyses of Amino Acid Site-Level Variation

**Fig. 7.**
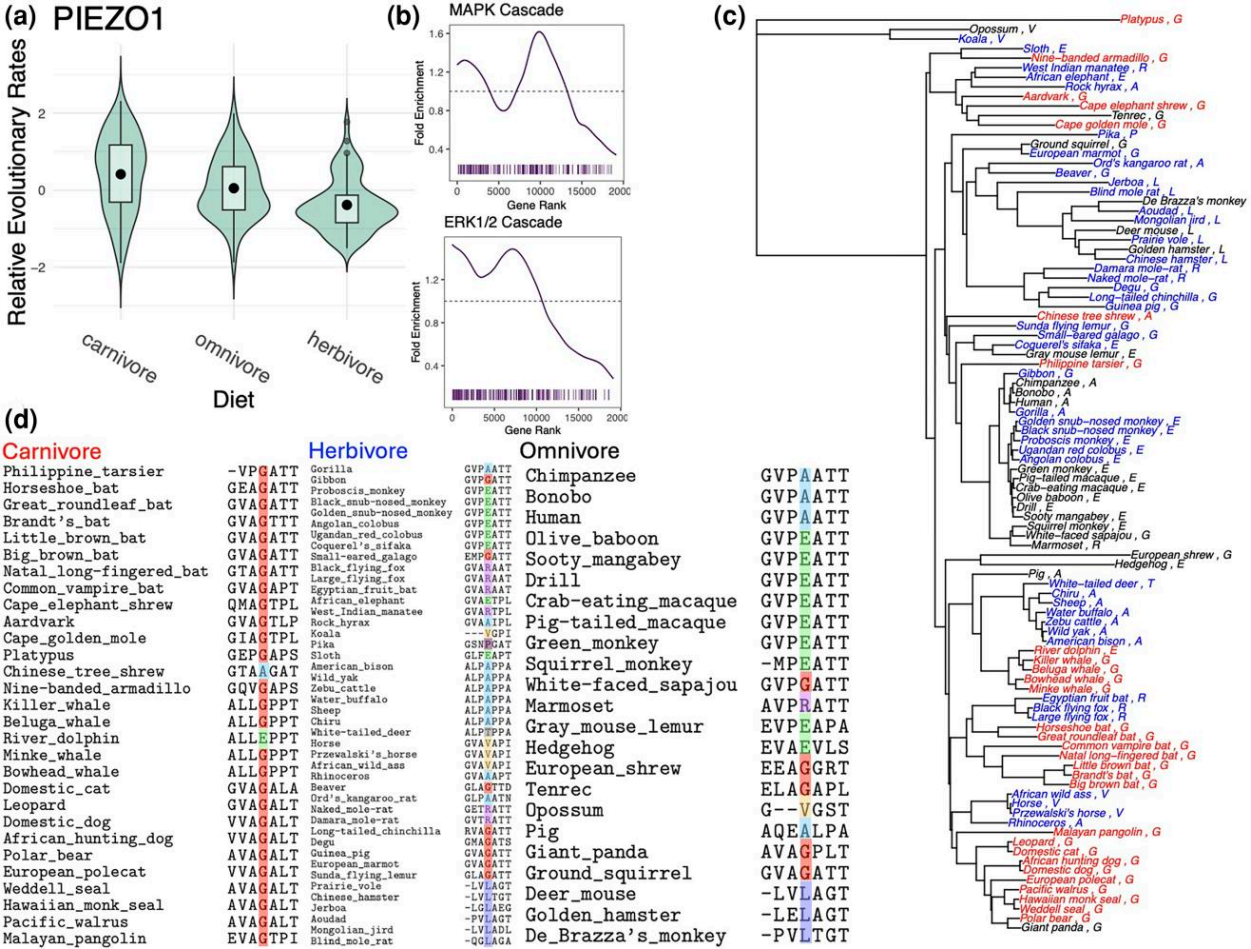
PIEZO1 is a gene identified by categorical RERconverge with a clear site-level pattern based on diet. a) Violin plot of relative evolutionary rates of PIEZO1 showing a significant difference in rates between diet categories. b) Fold enrichment and bar code plots of the MAPK cascade and positive regulation of ERK1/2 cascade GO pathways which had significant enrichment under the categorical carnivore/herbivore pairwise test. c) Phylogeny with carnivores, herbivores, and omnivores. The identity of the highlighted residue is written next to the species name. d) Segment of the multiple sequence alignment with the residue with site-level differences among diets highlighted. Most carnivores possess a glycine whereas there is much more variety at this position in herbivores and omnivores.

After using categorical RERconverge to detect diet relevant genes, we propose that a user begin to explore amino acid site-level variation to understand the nature of the molecular evolutionary changes in a relevant gene. We decided to focus on the PIEZO1 gene due to its important role in the digestive system (He et al. 2023) and significant difference in evolutionary rates across groups as detected by our categorical RERconverge method (omnibus test permulation *P* = 0.029) (Fig. 7a). PIEZO1 is a mechanosensitive ion channel which opens in response to mechanical forces. The ensuing influx of cations, especially Ca^2+^, is involved in many downstream processes including activation of integrin, ERK1/2-MAPK, and other signaling pathways involved in cell differentiation during digestive system development (He et al. 2023). Interestingly, other integrin-related genes were among the most significant genes identified by the categorical RERconverge analysis (supplementary fig. S6, Supplementary Material online, supplementary Categorical RERconverge Results, Supplementary Material online, supplementary data S2, Supplementary Material online), and both positive regulation of ERK1/2 cascade (permulation *P* = 0.0175) and MAPK cascade (permulation *P* = 0.0143) pathways were enriched among the categorical carnivore/herbivore post hoc test results (Fig. 7b). Together, these results may imply a candidate role for PIEZO1 mediated cell differentiation during digestive system development in the evolution of diet type, though more follow-up research would be necessary.

An analysis of the PIEZO1 alignment identified at least one position with site-level differences in conservation between diet categories. A site-level difference was determined by comparing at each site the set of amino acids possessed by the species in one diet category with the sets of amino acids possessed by species in one or more of the other diet categories; filtering to sites with the lowest similarity scores between these sets; and only keeping sites for which plotting the tree indicated that differences in amino acid composition across diet categories were independent of clade membership in the phylogeny (Fig. 7c). The residue corresponding to residue 1,835 in the human PIEZO1 amino acid sequence is glycine in all but two of the 29 carnivore lineages (ignoring lineages with a gap character). While 8 of the 44 herbivore lineages and 5 of the 23 omnivore lineages also possess a glycine, this residue is more variable among herbivores and omnivores (Fig. 7d). This suggests that carnivores may be less tolerant to changes in this position compared to herbivores and omnivores. In human PIEZO1, this residue falls within a disordered region between two transmembrane domains (Surhone et al. 2023). It is also the site of a known, likely benign, missense variant within human populations that changes alanine to valine (Sherry et al. 1999).

### Permulations Results

Previously, binary and continuous permulations were demonstrated to account for nonuniform null distributions and to refine pathway enrichment results by taking nonindependence of gene ranks into account (Saputra et al. 2021). We verified that categorical permulations corrected for nonuniform null distributions while phylogenetic simulations did not (supplementary fig. S3a, Supplementary Material online, supplementary Categorical Permulations Results, Supplementary Material online). We also demonstrate that categorical permulations have the same ability to refine pathway enrichment results. For example, we observed that olfactory genes commonly cluster together in rank (supplementary fig. S3b, Supplementary Material online). As a result, olfactory signaling and olfactory transduction were two of the strongest pathway enrichment results according to non-permulated *P*-values (*P* = 3.49 × 10^−5^ and 6.94 × 10^−5^). These had lower *P*-values than the starch and sucrose metabolism pathway from the canonical gene set (*P* = 1.00 × 10^−4^) (Subramanian et al. 2005; Liberzon et al. 2011), which has a clear relationship to the diet phenotype. After permulations, starch and sucrose metabolism was retained as the top result (permulation *P* = 0.0021), while olfactory signaling and olfactory transduction were no longer among the top enriched pathways (permulation *P* = 0.2866 and 0.3320) (supplementary fig. S3b, Supplementary Material online).

### Permulations Timing

Categorical permulations demonstrate how we can expand phylogenetic permulations to work with categorical phenotype data, however the current implementation of this method has limitations. The rejection sampler (see Methods, step (i)) becomes very slow for large trees with greater than three categories, especially in phenotypes which are more highly clustered according to phylogeny. To allow for comparison in runtimes between the number of categories used in the permulations, the diet phenotype was further subset into six phenotypes (omnivore, carnivore, herbivore, insectivore, piscivore, and anthropivore).

We compared both the effect of species number included in the analysis and number of categories used in the analysis on the time required to complete permulations. While the number of species did not appear to have an effect on permulation time, the number of categories in the analysis had a drastic effect (supplementary fig. S12, Supplementary Material online). We used linear regression to assess the effect of the number of species included in the analysis on the time to perform the analysis. In the two-category analysis, where species number appeared to be strongly correlated with permulation time (*R*^2^ > 0.5), the effect appeared to be minor (0.02 s per species) (supplementary fig. S10a, Supplementary Material online). For analyses with three or more categories, species number appeared to have little to no effect on permulation runtime (*R*^2^ = 0.03, 0.14, 0.01, respectively) (supplementary fig. S10b to e, Supplementary Material online).

**Fig. 8.**
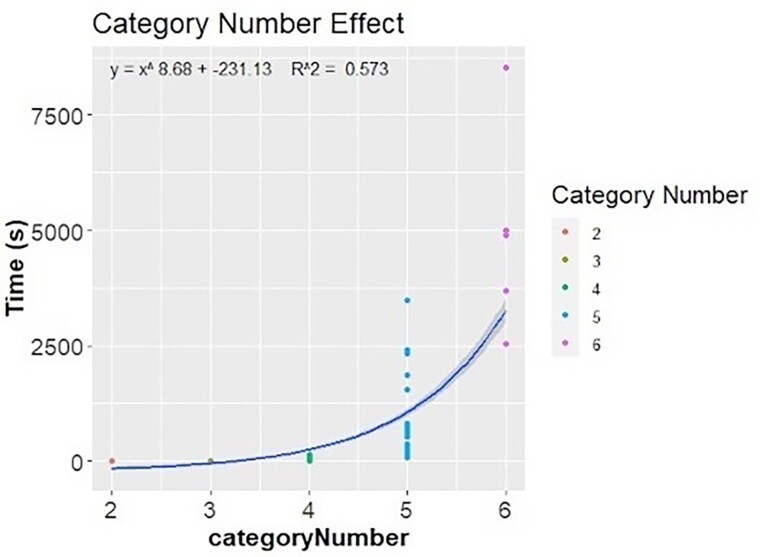
Inferred exponential relationship between number of categories in a phenotype and time required to complete permulations.

We first compared the effect of category number on permulation time by comparing the average time to complete a set of permulations with each number of categories. The time required increased dramatically with the number of categories. Time (seconds) per phenotype set increased exponentially (*y* = *x*^8.11^ − 223.66, *R*^2^ = 0.567) as the number of categories increased, ranging from 7.2 s for two categories to 4,933 s for six categories (Fig. 8; supplementary table S2, Supplementary Material online).

Additionally, we directly compared identical species sets with different category numbers, based on combining mergeable phenotypes (supplementary fig. S13, Supplementary Material online). This analysis provided further evidence that the runtime increase grows more pronounced as additional categories are added even while keeping the set of species the same (see supplementary Permulations Timing Results, Supplementary Material online).

### Permulations Relaxation

As shown above, adding categories increases the runtime of permulations exponentially, as currently implemented. This limits the number of categories that can feasibly be analyzed using categorical RERconverge if permulations are to be performed. To address these limitations, we developed a relaxed version of permulations that does not enforce such strict rejection constraints during the first step (supplementary Methods, Supplementary Material online). Through repeating this same analysis of permulation runtimes across different phenotype sets with different degrees of relaxation, we demonstrate that using a small relaxation of the phenotype matching criterion (10%) can significantly speed up the permulations, especially as category numbers increase, and is not expected to affect the plausibility of the phenotype structures of the permulated trees (supplementary fig. S8, Supplementary Material online, supplementary fig. S11, Supplementary Material online, supplementary tables S3 and S4, Supplementary Material online).

## Discussion

Here, we extend the RERconverge method to include the analysis of categorical traits. Our extension includes updated methodology for ancestral trait reconstruction, statistics that can be applied to categorical variables, and the adaptation of permulations to correct for the effects of uneven phylogenetic distribution of categorical traits (Fig. 2). To benchmark these features, we used the enrichment of the diet-related pathways “digestive tract development” and “abnormal digestion” in the categorical analysis of diet traits. Our results on the “digestive tract development” pathway demonstrate the importance of improving ancestral trait reconstruction with CTMM, even when binary phenotypes are used (Fig. 4a and b). Though ancestral state reconstruction by maximum likelihood applied to a CTMM is a standard approach, our implementation within the RERconverge workflow is crucial to developing a method that allows users to compare relative evolutionary rates across more than two phenotypic categories simultaneously (Fig. 2). Our results on the “abnormal digestion” pathway demonstrate that categorical analysis can identify convergent evolution in relevant genes even when a series of binary pairwise measurements fail (Fig. 4c and d, Fig. 5) Our methods have been added to the RERconverge package (Kowalczyk et al. 2019) and are already freely available. For those intending to use our methods, if runtime is a concern, when comparing four or more categories we recommend using a relaxation level of 10% for permulations. While exact effects differ between trees, using 10% relaxation provides order of magnitude speed increases without significantly affecting results (supplementary fig. S8, Supplementary Material online, supplementary fig. S11, Supplementary Material online).

In addition to comparing results across different ways of running RERconverge, we compared categorical RERconverge to an ANOVA approach (Revell 2012) and to a delta statistic approach (Borges et al. 2019). Although the ANOVA approach has been extensively used, it performed poorly in finding diet signal relative to our RERconverge updates and the delta statistic (supplementary fig. S4, Supplementary Material online). This is perhaps unsurprising given that phylogenetic ANOVA only considers phenotypes for extant species, whereas RERconverge includes all branches within the phylogeny of the sampled species, leading to different sample sizes (and hence different power) for the same species set. The delta statistic and categorical RERconverge both show diet-related signal, but genes driving the signal differed between the two methods (Fig. 6). For example, for the “abnormal digestion” pathway, the enrichment results were similar between the categorical omnibus test we developed and the delta statistic approach. However, different genes contributed to the enrichment; e.g. ITGB1 and TREH were captured by RERconverge omnibus test while SLC10A2 and PNLIP showed greater association in the delta statistic analysis. These differences may stem from methodological differences—the delta statistic detects a different type of evolutionary signal from RERconverge—but they may also be driven by potential bias in genes captured by the delta statistic (Fig. 6). In the case of the diet phenotype, the delta statistic was biased toward capturing signal driven by slower evolution of carnivore lineages compared to other lineages. This trend may be the consequence of the delta statistic using certainty in ancestral state inference to identify phylogenetic signal. Reconstruction certainty, among many factors, depends on inferred phylogeny branch lengths which are related to evolutionary rates. We may expect a trait's ancestral history on a phylogeny to be inferred with greater or less certainty when the branch lengths also exhibit a certain pattern, since many ancestral likelihood inference methods model the probability of phenotype change as a function of branch length (Pagel 1994; Pupko et al. 2000; Paradis et al. 2004). Thus, a certain pattern in branch lengths may contribute to the observed phenotype structure. In the case of mammalian diets, slower evolutionary rates among carnivores may improve the certainty of inferring carnivorous ancestors, corresponding to what we would expect from our maximum likelihood reconstruction on the master tree (supplementary fig. S1, Supplementary Material online), and leading to higher delta statistics in genes with slower evolutionary rates in carnivores. However, it is unclear to what extent these patterns in branch lengths and ancestral inference certainty reflect the real biology or methodological uncertainty in the inferred branch lengths, gene tree structure, and ancestral likelihoods. Although the results are only explored for one trait, our results would suggest that the delta statistic may be biased toward detecting genes with patterns of evolution driven by single pairwise comparisons evolving in one direction. That limitation is compounded by a lack of post hoc testing available through the delta statistic to evaluate which comparisons contribute to the omnibus signal.

Our methodological updates and analysis provide new insights into the evolution of diet. Throughout the course of the research, we created a ranked list of genes that have convergently evolved with diet across mammals (supplementary data S2, Supplementary Material online). These genes were enriched for pathways like “abnormal digestion” and “digestive tract development”, which suggest that there is substantial signal within the analysis (supplementary data S3, Supplementary Material online). To confidently connect the associations with the evolution of diet, examination with other tools, including the Hyphy, BUSTED-PH model (Murrell et al. 2015), should be performed. The functional validation of specific genes detected through this analysis would also increase the confidence in the individual results. However, there are already a number of promising candidates identified. For example, the PIEZO1 gene (omnibus test permulations *P* = 0.029) of the peripheral nervous system has been extensively studied in the context of diet (He et al. 2023). Disruptions of PIEZO1 function in human and mouse models lead to differences in digestion and iron metabolism, both related to the evolution diet phenotypes (Ma et al. 2021; Amado et al. 2024) This brings up the possibility that evolutionary annotations of function could help to interpret rare genetic variation associated with human disease phenotypes.

Future work may further improve our understanding of dietary evolution by integrating larger sets of species and more fine-scale resolution of dietary phenotypes combined with different reconstruction methods, beyond the categories we have included here. Additionally, diet can be represented in ways other than a non-ordinal categorical trait, such as the varying degrees of carnivory expressed by different mammals (Wilman et al. 2014; Pollard and Puckett 2022; Pollard et al. 2023). Evaluation of diet from the continuous perspective and comparing those results with categorical analyses would provide confidence and nuance to the genes and pathways connected with diet evolution.

There are already many applications of tests for convergent evolution across a variety of genomes (Marcovitz et al. 2019). The reduction in cost of genome sequencing has led to an encouraging proliferation of comparative genomics resources. In recent years, these have expanded from dozens of species (Zhang et al. 2014; Hecker and Hiller 2020) to hundreds of species (Christmas et al. 2023). Additional coordinated large scale genome sequencing efforts continue to expand resources and genome quality (Teeling et al. 2018; Rhie et al. 2021). With these resources, new computational approaches are being developed to obtain more accurate protein annotations from these diverse genomes (Kirilenko et al. 2023). In parallel, field work and coordinated databases are providing increased high-quality data on trait annotations (e.g. Stefen et al. 2022). As the genome sequences, protein annotation, and trait annotations continue to improve, there will be an increasing need for methods that can handle increased scale of data and trait complexity. The improved ability of RERconverge to handle categorical traits provides a good foundation for the community to use newly emerging resources to make new biological discoveries.

## Methods

### Mammalian Coding Sequence Alignments and Trees

Mammalian coding sequence alignments were extracted from a whole-genome multiple alignment of 120 mammal species (Hecker and Hiller 2020). Coding exons were extracted based on human genome (hg38) coordinates from GENCODE version 36 (GENCODEV36) (Frankish et al. 2019). Transcripts were chosen only from “protein_coding” genes and the “canonical” isoforms were used, that is a single isoform meant to represent each gene. Exon alignments were extracted from a maf format alignment with human as reference using functions from the RPHAST package (Hubisz et al. 2011). RPHAST was also used to enforce the human reading frame and translate all sequences to amino acids. The resulting amino acid alignments were used to infer gene trees for each gene using a fixed species tree topology using the phylogenetic package phangorn (Schliep 2011).

### Diet Phenotype Assignment

Diet data were collected from each species from Walker's Book Of Mammals (Nowak 1999). Species were classified on the exclusive or nearly exclusive (90%+) composition of their diet. Those consuming plant matter were considered Herbivores (49 sp.), those consuming arthropods were considered Insectivores (18 sp.), and those consuming terrestrial vertebrates were considered Carnivores (10 sp.). Species which exclusively consumed aquatic vertebrates, shellfish, and/or bivalves were considered Piscivores (nine sp.). Species which consumed more than one type of food source were considered Omnivores (25 sp.). Species which were capable of surviving off of primarily human-derived food sources (e.g. food waste, cardboard, and plaster) were classified as Anthropivores (five sp.). Anthropivores, though a poorly defined phenotype, represent a subset of omnivores, and as such this category was included to perform direct comparisons in timing between category numbers.

All six of these phenotypes were used in the permulations timing and relaxation analyses (supplementary file S2, Supplementary Material online (permulations phenotype vector); supplementary file S3, Supplementary Material online (tree image)). In addition, the grouping of Carnivore–Piscivore was combined to the phenotype Vertivore (19 sp.) and Omnivore–Anthropivore combined to the phenotype Generalist (30 sp.). Piscivores and anthropivores are subsets of carnivores and omnivores, respectively, and thus provided biologically sound phenotype merges to allow for direct comparison between different numbers of categories used to describe the same species set.

In the main three-way analyses, where the primary goal was to demonstrate the categorical method rather than to identify fine-grained genetic difference between diet types, certain simplifications were made to compress all the species in the data set into three groups. To this end, insectivores were considered carnivores since, not distinguishing between vertebrates and invertebrates, insectivores are a specialized carnivore. Additionally, some species which were considered omnivores in the six-category analysis were considered carnivores in the three-category analyses, due to those species consuming a mixture of vertebrates and invertebrates, which are not distinguished in the three-category analyses.

### CTMM Ancestral Reconstruction

Further details beyond what are presented in the Results section are provided in the supplementary Methods, Supplementary Material online.

### Permulations

Permulations generate permuted phenotype trees that maintain the phylogenetic relationships in the data. For instance, binary permulations maintain the same number of foreground species and the same structural relationships between foreground species (Saputra et al. 2021). Once the permulated phenotypes are generated, the association between RERs and each permulated phenotype is computed for each gene using the same RERconverge functions that calculate gene association statistics in the main analysis (Fig. 2). Empirical *P*-values are then computed for each gene as the proportion of null effect sizes more extreme than the observed effect size for that gene (Fig. 2).

The categorical permulations algorithm employs three steps to ensure that the permulated phenotype contains the same number of species with each trait value as the original phenotype: (i) Rejection sampling: phenotypes are simulated from the CTMM that was used to reconstruct the ancestral history of the trait. As with binary and continuous permulations, the simulation is based on a phylogeny with branch lengths representing the average genome-wide evolutionary rate along that branch. Any simulated phenotype in which there is not the same number of extant species with each trait value as the original phenotype is rejected. (ii) Permutation of internal traits: the simulated values for internal species are ignored. Instead, the originally inferred internal trait values are permuted and assigned to internal species in the permulated phenotype. (iii) Re-organize internal traits: a search technique similar to simulated annealing is used to re-organize the internal states relative to the simulated extant states to improve the likelihood of the permulated phenotype (supplementary fig. S9, Supplementary Material online).

Further details on the categorical permulations algorithm are provided in the supplementary Methods, Supplementary Material online.

### Permulations Timing

Permulations, in particular the generation of permulated phenotype trees, were performed using the same species as the main analysis. Each time value reported is the time required to run 5 permulations to account for fluctuations in each permulation's generation time. Permulations were performed on all combinations of phenotypes, including the merged Vertivore and Generalist phenotypes, for a total of 24 two-category comparisons, 34 three-category comparisons, 24 four-category comparisons, 8 five-category comparisons, and 1 six-category comparison, for a grand total of 91 category sets. Further details are provided in the supplementary Methods, Supplementary Material online.

### Categorical Statistical Analysis

Relative evolutionary rates are associated with the categorical phenotype in one of two ways. The default approach is to use a nonparametric Kruskal–Wallis test followed by post hoc Dunn tests (Ogle et al. 2023). There is also an option to use an ANOVA test followed by post hoc Tukey tests (Foundation for Statistical Computing, n.d.). The Kruskal–Wallis test reports the epsilon squared effect size computed as $\frac{H}{n\;-\;1}\;$ where *H* is the Kruskal–Wallis test statistic and *n* is the total number of observations (branches), and the corresponding *P*-value (King et al. 2018). The Dunn test reports the *Z* statistic for each pairwise test and the *P*-value after adjusting for multiple comparisons. The ANOVA test reports the eta squared effect size computed as $\frac{\mathrm{sum}\;\mathrm{squares}\;\mathrm{effect}}{\mathrm{sum}\;\mathrm{squares}\;\mathrm{effect}\;+\;\mathrm{sum}\;\mathrm{square}\;\mathrm{residual}}$, and the corresponding *P*-value. The Tukey test reports the difference between each pair of means and the *P*-value corrected for multiple comparisons.

### Other Methods

Details on the other methods (Binary RERconverge, phylANOVA, and the delta statistic) are provided in the supplementary Methods, Supplementary Material online.

## Supplementary Material

msae210_Supplementary_Data

## Data Availability

RERconverge GitHub: https://github.com/nclark-lab/RERconverge. Permulations Timing/Relaxation Analysis GitHub: https://github.com/MichaelTene7/CategoricalPermulationsTiming. Alignments and Trees zip file available on KiltHub: https://doi.org/10.1184/R1/24897987.v1.
