# Supplementary material for: RERconverge Expansion: Using Relative Evolutionary Rates to Study Complex Categorical Trait Evolution
Source: Mol Biol Evol. 2024 Oct 15;41(11):msae210. doi: 10.1093/molbev/msae210 (PMC11529301; doi:10.1093/molbev/msae210)
Supplement: msae210_Supplementary_Data [file msae210_supplementary_data.zip › supplement.pdf]

Supplementary Results

Categorical RERconverge

To further emphasize the robust ability of categorical RERconverge to identify different pairwise patterns of shifts in evolutionary rates, we look at the distributions of RERs of two genes within the digestive tract development pathway, ITGB4 and ITGA6 (**Fig. S6**). These genes show different patterns relative to omnivores; ITGB4 illustrates that the distributions of relative evolutionary rates may differ significantly between all three categories, alternatively ITGA6 illustrates the case where only the RERs of herbivores differ significantly from carnivore/omnivores. Regardless, both genes are identified by our categorical method. This result is particularly compelling since these genes encode integrin subunits which tend to associate to form a heterodimer (O'Leary et al., 2016), and interestingly both show acceleration among carnivores compared to herbivores (**Fig. S6A,B;** ITGB4 $p = 5.662 \times 10^{-14}$, adjusted $p = 1.0719 \times 10^{-9}$, permulation $p = 0.0$; ITGA6 $p = 5.74 \times 10^{-5}$, adjusted $p = 0.00407$, permulation $p = 0.006$).

phylANOVA Comparison

Unlike the gene results, the quantile-quantile plots of pathway enrichment p-values were less informative of method performance. For one, the pathway enrichment p-values were computed in different ways for the RERconverge analyses compared to phylANOVA, and the RERconverge approach, which uses a pathway-level application of permulations, is more conservative due to methodological differences (see Supplementary Methods). Secondly, the power to detect enriched pathways reflected in the quantile-quantile plots does not seem to correspond with more refined diet-related results, as we illustrate with the carnivore/herbivore MGI pathway enrichment results (**Fig. S6**).

Refinement of diet-related results is measured as the proportion of digestive system, liver/biliary system, or metabolism related pathways out of the total number of pathways identified by that method (**Fig. S6B**). Significant enrichment was determined by meeting an adjusted p-value threshold of 0.15. We chose a more relaxed adjusted p-value threshold than 0.05 because only two pathways had adjusted p-values less than 0.05 using the phylANOVA method. Using a more relaxed p-value reflects that while the analysis may be statistically underpowered due to hard limitations like the number of species included in global alignments, the top results could still be meaningful. The binary and categorical RERconverge methods had more power, and therefore many more enriched pathways were detected at an adjusted p-value threshold of 0.15. To obtain a reasonable number of top pathways meeting an adjusted p-value threshold of 0.15, we also apply a permulation p-value threshold of 0.025. This choice reflects that the permulation p-value is correcting for non-independence in gene ranks and in the phylogeny, so a low permulation p-value is indicative of the significant adjusted p-value being due to association with the phenotype rather than other sources of correlation in the data. While some of the refinement in the pathway enrichment results are likely due to applying this additional significance threshold, it still reflects an advantage of RERconverge over phylANOVA since permulations cannot be performed with phylANOVA. Furthermore, in this case, none of the enriched pathways detected by phylANOVA met our criteria for diet-relevance, so even applying additional thresholds or filtering would not improve the proportion of relevant pathways identified by the phylANOVA method.

While the proportion of diet-relevant results discovered by categorical and pairwise binary RERconverge suggests both methods are sufficiently powered to detect diet-relevant pathways, the same is not found for phylANOVA which shows lower power to detect these diet-specific pathways (**Fig. S6B**).

Categorical Permulations

We expect categorical permulations to account for non-uniform null distributions. To determine if this was the case, we plotted histograms and quantile-quantile plots of the parametric and permulation p-values (**Fig. S3A**). The categorical pairwise tests all showed a large enrichment for high parametric p-values of around 1. This enrichment of high p-values is also detected in the permulations and is thus no longer present among the permulation p-values. This demonstrates that the enrichment of high p-values is most likely specific to this statistical test and sources of nonindependence in the data rather than representing a true pattern specific to the diet phenotype.

Notably, the simulation p-values calculated by phylANOVA still have an enrichment of high p-values near one (**Fig. S3A**). In fact, this enrichment is even more extreme than that of the categorical pairwise parametric p-values. PhylANOVA simulates the RERs using a Brownian Motion model, so while this takes phylogenetic dependence into account, it doesn't account for other systematic variation such as nucleotide content or genome quality that can affect the RER values themselves and lead to non-uniform null distributions. The enrichment of parametric and phylANOVA simulation p-values near 1 and removal of such enrichment by permulations was observed across all the pairwise tests (**Fig. S3A**). Thus, unlike permulations, phylogenetic simulations were not able to correct for this pattern.

Permulations Timing

In order to create identical species sets with different category numbers, we combined mergeable phenotypes. For instance, [Herbivore-Carnivore-Piscivore] was compared to [Herbivore-<Carnivore&Piscivore>]. The singly-combined phenotype sets were found to be faster than the unmerged phenotypes, particularly when there were more than 4 categories in the unmerged analysis (**Fig. S13**). On average, the merged phenotypes were 23%±14%, 284%±144%, and 1502%±762% faster at 3v2, 4v3, 5v4, respectively once outliers were removed. Two 6v5 comparisons exist in the dataset, with the merged phenotype being faster but to highly different degrees (speed increase of 112% and 3319%). The double combination phenotypes were found to be dramatically faster than the unmerged phenotypes, being 481%, 5014±3484%, and 32,220% faster at 4v2, 5v3, and 6v4 respectively. Note that these results are from a relaxation level of 10%, and as such the differences between category number is even more pronounced in unrelaxed permulations (**Table S3, Fig. S12**).

Permulations Relaxation

We tested the speed increase from relaxation by running the timing test for each phenotype set at 0%, 5%, 10%, and 20% relaxation. The speed increase from relaxation is dependent on the number of categories, increasing non-linearly as the number of categories increases (**Fig. S11, Table S4**). For two categories, the improvements are marginal: with a mean improvement across all phenotype sets of 1.3%, 12%, and -1.5% for 5%, 10%, and 20% relaxation respectively. For three categories, the improvements were 3%, 57%, and 63%. For four categories, the improvements were 18%, 646%, and 1991%, dramatically faster than unrelaxed permulations. While unrelaxed versions of 5 and 6 category phenotype sets could not be completed, the 20%

relaxed permulations were faster than the 10% relaxed permulations by 964% for five category sets, and by 40,000% for the six category set. When outliers were removed from the data, the speed increase was more pronounced.

The relaxed and unrelaxed permulation p-values had a mean difference of < 0.03 for the omnibus and all pairwise comparisons for both the smallest (IPC) and largest (HMG) phenotype set, and there was no significant difference in the effect of relaxation on p-value between IPC and HMG.

In order to verify that a small relaxation of 10% on the extant species counts while maintaining internal category counts exactly as they are in the original ancestral reconstruction did not noticeably reduce the evolutionary plausibility of the permulated phenotypes, we generated 10,000 permulations and computed the log likelihood of each one (see Supplementary Methods, equation 1). We plotted the distributions of the log likelihoods of the simulated phenotypes that passed the rejection sampler (see Supplementary Methods, step 1) against the distributions of the log likelihoods of the permulated phenotypes that were generated from these simulations (see Supplementary Methods, steps 2 & 3). We expect plausible permulated phenotypes to have log likelihoods similar to the simulations. The relaxation did not greatly affect the distribution of log likelihoods of the permulated phenotypes (**Fig. S8**). Thus using a small relaxation can significantly speed up the permulations, but is not expected to harm the evolutionary plausibility (log likelihoods) of the permulated trees.

Supplementary Methods

CTMM Ancestral Reconstruction

Maximum likelihood estimation is used to infer a transition rate matrix, Q, from the user supplied phylogeny, rate model, and extant phenotype data. The phylogeny is the master tree which includes all species in the analysis and has branch lengths representing average genome-wide evolutionary rates. The transition matrix is inferred using the *fit_mk* function from the castor package (Louca & Doebeli, 2018), and is used to compute the marginal ancestral likelihoods at each node. Code for the computation of ancestral likelihoods is heavily based on *ace* from the package ape, using the same double pass algorithm but is modified to work with unrooted, non-dichotomous trees (Paradis et al., 2004). Each node is then assigned the state with the maximum marginal likelihood. Though this is not the same as the assignment of states that maximizes the joint likelihood, marginal ancestral likelihoods can be computed rapidly even for large phylogenies.

The rate model describes the number and position of free rate parameters in the transition rate matrix. In order to compare rate models, RERconverge implements a likelihood ratio test which computes the log likelihood of the fitted transition matrix under each user supplied rate model. Pairwise comparisons are made between each rate model with its more complex rate models and the likelihood ratio and p-value is computed for each comparison. If the two rate models are nested (the simpler one is a special case of the more complex one), then the likelihood ratio is distributed as a chi-squared with degrees of freedom equal to the difference in the number of free parameters between the simpler and more complex model and the p-value is determined accordingly (Pagel, 1994). Other packages implement a similar test for nested models including

anova in the ape package (Paradis et al., n.d.). However, the likelihood ratio test in RERconverge works with both nested and non-nested models and will automatically detect whether the models are nested or not. For non-nested models, Monte Carlo simulations are used to determine an empirical p-value for the likelihood ratio (Pagel, 1994).

For binary phenotypes, the user can choose whether branches are considered foreground based on the state of possessing the convergent trait or based on the transition from not possessing to gaining the trait. Both methods were tested for hairless species with no large impact found (Kowalczyk et al., 2022). However, with categorical traits especially when the ancestral trait is unclear, the number of possible transition types is quadratic with respect to the number of categories. Thus we decided to assign edges based on state rather than transition. Edges were assigned the state of their descendant nodes such that all edges leading to extant species are assigned based on the observed phenotype.

In order to handle missing species, RERconverge computes "paths" (Kowalczyk et al., 2019). When species are missing, certain nodes are no longer necessary so edges will be combined into "composite" edges and the paths describe what state values to use for these composite edges. Categorical RERconverge assigns the composite edges the state of the most recent edge in the master tree. This ensures that composite edges leading to extant species always use the observed state.

Permulations

Algorithm Details

In step 2, in which internal trait values are permuted and assigned to internal species in the permulated phenotype, the assignment is weighted by the ancestral likelihoods calculated from the simulated tip values. This ensures that the initial permutation of internal traits is more optimal than a completely random shuffle. In the following step, the permuted internal states are reorganized relative to the simulated extant states to improve the likelihood of the permulated phenotype. This is accomplished through a series of swaps. Pairs of candidate nodes are suggested based on which internal nodes from step (2) disagree most with the ancestral likelihoods at that node. A swap is made, and if the swap improves the likelihood of the tree, then the swap is kept. To avoid getting stuck, the swap is also made with a small probability even if it does not improve the likelihood of the tree. This generates a plausible trait history that exactly matches trait category counts and has a comparable likelihood to the original simulation.

Algorithmically step 3 of categorical permulations is computed as follows: Let Q be the instantaneous transition rate matrix that was fit on the phenotype data. Let A be the matrix of ancestral likelihoods where each row is a node and each column is a phenotype state. The initial likelihood of the tree after step 2 is computed.

$$likelihood = \prod_{i=1}^{num\ edges} (e^{Qt_i})_{x_i, y_i} \quad (1)$$

where t_i is the length of edge i, e^{Qt_i} is the transition probability matrix, x_i is the ancestor on edge i, and y_i is the descendant on edge i. For each internal node i currently assigned state y, ratios are computed for all other states x.

$$ratio_{i,x} = \frac{A_{i,x}}{A_{i,y}}$$

Thus a large ratio indicates that node i prefers to be in state x over its current state y.

A ratio, $r1$, is selected at random with more weight placed on larger ratios so that poorly assigned nodes are more likely to be swapped. Let $r1 = ratio_{i,x}$ where node i is currently in state y. Then, a second ratio, $r2$, is selected from a list of ratios, $ratio_{j,y}$, where each node j is currently in state x. Once again, more weight is placed on larger ratios. A swap in which node i is switched to state x and node j is switched to state y is then proposed.

The likelihood of the tree is recomputed under the swap. If the likelihood improves, then the swap is kept. If the likelihood does not improve, then the swap is made with probability $u = exp(- dh/T_k)$ where $dh = log(\frac{likelihood\ before\ swap}{likelihood\ after\ swap})$. Thus if dh is large (the swap is very unfavorable), u will be small and it is less likely to make the swap. T_k represents the "temperature" which is a common feature of simulated annealing algorithms. The temperature begins high during early iterations and decreases as the iterations go on. Thus, in the early iterations u is larger, and unfavorable swaps are made with a higher probability. This allows the algorithm to try more swaps, especially early on, to increase the overall number of potential state configurations it explores, even if some of those swaps initially decrease the likelihood. Only allowing swaps that improve the likelihood may cause the trees to get stuck before they reach a more favorable state assignment.

At the end of each cycle k, the new temperature is calculated as $T_k = \frac{T_0}{1 + \alpha k}$ where T_0 and α were chosen semi-arbitrarily and are currently 10 and 0.9 respectively. 100 cycles were run, each with 10 iterations for a total of 1000 iterations.

In order to determine the effectiveness and importance of each step in the above algorithm, we plotted the distributions of tree log likelihoods after each step (**Fig. S9**). After step 2, the log likelihoods decrease because we replace the simulated internal states with a permutation of the original internal states. However, after step 3, the completed permulated trees (tan) return to having the same or improved likelihoods compared to the simulations (blue). Thus, plotting the log likelihood distributions after each step demonstrates why step 3 is extremely important for ensuring the overall plausibility of the permulated phenotypes and thus maintenance of the types of phylogenetic dependencies present in the original phenotype tree.

Two different methods were originally developed for permulations; the CC (complete case) method which generates permulated phenotypes of the complete topology and SSM (species subset match) which is more time intensive in order to correct for missing species among some gene trees (Saputra et al., 2021). In the CC method, each permulation of the complete topology has the same number of species in each category. However, when a gene tree has missing species, the corresponding branches in the complete topology are ignored when associating the phenotype with the RERs of that gene. These branches may be assigned different categories (states) in each permulation, so the exact number of branches assigned to each category in each permulation for any given gene is not constant. Despite this drawback, the CC method is significantly faster, so we only implemented a categorical approach for the CC method.

Calculation of Permulation P-values

From the empirical distribution of null effect sizes, one sided p-values are computed for the omnibus test and two-sided p-values are computed for the post hoc tests according to the equations shown below.

$$p_{permulation} = \frac{\sum_i \mathbb{I}(ES_{null,i} \geq ES_{obs})}{N}$$ (one-sided)

$$p_{permulation} = \frac{\sum_i \mathbb{I}(|ES_{null,i}| \geq |ES_{obs}|)}{N}$$ (two-sided)

Where i indexes over the effect sizes computed for each permulation, ES is the effect size, and N is the number of permulations performed. (In practice, N is the number of permulations for which the returned effect size for that gene is not "NA". "NA" is returned in cases where, due to missing species, the permulated phenotype does not have enough species of one or more categories for association statistics to be calculated (see discussion of CC vs. SSM permulation methods above on the impact of missing species)). The numerator contains the indicator function, which evaluates to one when the expression inside is true and to zero otherwise.

Permulations Timing

Permulations were performed using both the SYM and ER rate models, for which the timing patterns broadly matched. The ARD rate model was not used, as it was not compatible with all of the phenotype sets. Trends were consistent across rate models, with the ER method being moderately faster than the SYM method; the difference in speed between ER and SYM varying slightly with relaxation, category set, and category number.

For each category set, timing tests were performed five times, for a total of 25 permulations. All timing measurements presented outside the supplement were performed using the SYM model at 10% relaxation, as performing 5 or 6 category comparisons at 5% and 0% relaxation was non-practical even when performing only 25 permulations. Data from the ER model follows similar trends, and the results for the ER model can be found in the supplement. Outliers were classified as any phenotype for which the time's average z-score (inclusive of outlier) > 2, while the dataset's standard deviation was greater than its mean. This allowed for detection of phenotypes which were dramatically affecting the mean of the dataset. All code used for permulation timing can be found here: [https://github.com/MichaelTene7/CategoricalPermulationsTiming]

Species number effect on permulations times

Tests for the effects of the number of species on permulation time were conducted by comparing the number of species in the tree to the permulation time for each number of categories. To account for differences caused by an increased number of categories, trees were only compared to others with the same number of categories.

For each number of categories, the effect of species number on permulation time was computed using linear regression. Calculations were performed both including and excluding outliers believed to be caused by phenotype clustering effects. Phenotype cluster effects are a phenomena observed where permulations take a much longer time if a single phenotype which comprises a large portion of the tree occurs in a monophyletic or nearly monophyletic clade; this results in very few valid alternates for use as a permulated tree, greatly increasing run time. More broadly,

phenotype cluster effects are a sign that permulations runtimes can be highly dependent on the structure of the phenotype and the tree topology. The rate limiting step of permulations is the rejection sampler (see Methods) in which the runtime depends most directly on the number of rejections made by the rejection sampler. The number of rejections comes down to how many ways exist to obtain the correct number of species counts per category in an evolutionarily feasible way (i.e. via simulation). This can lead to unintuitive results in which increasing the number of species while holding category number constant actually reduces the runtime (**Fig. S10C**).

Category number effect

Category number effect on permulation time was determined by comparing the time of permulations with the categories split against the time of permulations where the categories were merged. For instance, the time for the four-way comparison Herbivore-Insectivore-Carnivore-Piscivore was compared against the three-way Herbivore-Insectivore-<Carnivore&Piscivore>. This comparison was done for all possible combinations of the merged categories, with 3vs2 categories (10 comparisons), 4vs3 categories (16 comparisons), 5vs4 categories (10 comparisons), and 6 vs 5 categories (2 comparisons). Additionally, comparisons were made where both of the mergeable categories were combined. For instance, the five-way Herbivore-Carnivore-Piscivore-Omnivore-Anthropivore was compared against the three-way Herbivore-<Carnivore&Piscivore>-<Omnivore&Anthropivore>. This was done for all 4vs2 categories (1 comparison), 5vs3 categories (2 comparisons), and 6vs4 categories (1 comparison). Finally, the overall average times for each category number were

compared to each other. The average time for a category number was obtained by averaging the times for each of its phenotype sets.

Permulations Relaxation

Increasing the number of categories beyond three was demonstrated to significantly slow down permulations. The computational bottleneck occurs in the first step when phenotypes are simulated from the CTMM and simulations without the same number of extant species in each category as the original phenotype are rejected. Understandably, exactly matching counts becomes more challenging and thus slower as the number of categories increases. In order to handle these cases, a relaxed version of permulations was developed. This relaxation defines a percentage of the original counts that the simulated phenotype can fall within, and only rejects the simulation if the counts are not within this range. There are certain theoretical limitations to the current relaxation approach in that the internal category counts are still matched strictly to the original data. However, in practice we found that using a relatively small relaxation (of 10%) led to a significant speed improvement without reducing the evolutionary plausibility of the permulated phenotypes (as measured by the tree likelihoods) (**Fig. S8**) or causing large deviations in the p-values.

Relaxation effect on permulation speed

The full set of permulations were performed at relaxation levels of 0%, 5%, 10%, and 20% for the ER and SYM rate models, for all 91 category sets. Results presented are drawn from the SYM model, with the similar ER model results included in the supplement. For the 0% and 5% relaxation level on the 5 category and 6 category data, 10 permulations of each category set were

unable to be completed within 72 hours. As such, the mean time is displayed as a conservative minimum by assuming the set of permulations took 72 hours to run to completion, though the real number is likely far higher, as in fact only approximately half the permulations completed in that time.

We compared the time to complete each of the 91 category sets between the relaxation levels for each rate model. Additionally, we compared the time to complete each of number-of-category permulations, eg. time to complete all three-way category sets, between relaxation levels.

Relaxation effects on permulation results

Permulation P-value calculations were performed on both unrelaxed and 10% relaxed category sets, with 20,000 permulations of each. The resulting p-values for all 19137 genes in the Hiller dataset were then compared, and the mean difference between relaxed and unrelaxed p-values for all genes was calculated. The category sets used were the three-category sets: Herbivore-<Omnivore-Anthropivore>-<Carnivore-Piscivore> category set (the largest set) and the Piscivore-Carnivore-Insectivore category set (the smallest set). Category sets with more than 3 categories were not considered, due to the impracticality of running unrelaxed permulations with more than 3 categories.

Other Methods

Binary RERconverge

We compared the results to those of multiple binary RERconverge analyses. We employed two approaches to account for there being greater than two categories (**Fig 2B, C**). In the first

approach (method I in **Table 1**), one category was chosen as the foreground and the other two categories were set as background. This was performed three times; once for each category. In the second approach (method II in **Table 1**), we ran six pairwise binary RERconverge analyses. In these analyses, only species from two categories were included, the other species were removed from the tree and were not included in the calculation of relative evolutionary rates. For each pair of categories, two analyses were performed by switching which category was considered foreground.

However, the second binary method with herbivore foreground and carnivore background was excluded from the analysis because it had over a 10x slower permulations runtime than the other methods. The slowdown was caused by the rejection sampler because, to match foreground numbers, only permulated phenotypes including the same large clades as in the original data were being kept. This would incorrectly overestimate the inflation of low p-values.

Binary permulations with 10,000 permulated trees were performed on the remaining eight sets of binary gene correlations results. A modified version of binary permulations, which has been applied to hibernation and vocal learning phenotypes (Wirthlin et al., 2022; Christmas et al., 2023), was performed which approximately matches the extant and internal foreground species counts to within a defined range. Phylogenetic simulations are still used to replicate phylogenetic dependencies in the data but unlike original binary permulations, the permulated trees do not perfectly match the structure of the original phenotype due to greater complexity of the diet phenotype as compared to the phenotypes for which binary permulations was originally developed.

Rank-based pathway enrichments were calculated using the Wilcoxon Rank Sum test as implemented in the RERconverge package. Enrichment permulations were performed by calculating null enrichment statistics on all 10,000 null sets of gene correlation statistics and computing empirical p-values for each pathway based on the number of null enrichment statistics as or more extreme than the observed enrichment statistic.

Phylogenetic Signal

The delta statistic of a phylogenetic signal measures the degree to which a trait evolves according to a given phylogeny. It is based on the idea that when a trait is highly associated with a phylogeny, that phylogeny will be good at predicting the ancestral states of species with low uncertainty because the phylogeny effectively traces the evolutionary history of the trait. Thus the delta statistic summarizes the amount of uncertainty, measured as Shannon entropy, in a given set of ancestral likelihoods (Borges et al., 2019).

We used the publicly available, faster, python code from a recent update to this paper (Ribeiro et al., 2023) to calculate the delta statistic for each of our 19,137 gene trees with the 3-category diet phenotype. Ancestral likelihoods were calculated using the *ace* function from the ape package with an all rates different (ARD) rate model in order to be consistent with Borges et al. 2019.

All of the gene trees used in the RERconverge analysis were confined to have the same topology as the master tree. We believe this constraint may limit the degree of variation in phylogenetic signal observed between different genes and impact the ability of the delta statistic to identify

significant genes. Thus, we constructed unconstrained gene trees using iqtree (Nguyen et al., 2015) and repeated the delta statistic analysis.

Enrichments were calculated using the same approach as for all the RERconverge methods, using the Wilcoxon Rank Sum test as implemented in the RERconverge package. Enrichment permulations were not performed.

PhylANOVA

We used PhylANOVA from the phytools package to perform the phylogenetic simulation method as described by Garland et. al. 1993 (Garland et al., 1993; Revell, 2012). This method is designed to correct p-values for non-independence between species due to hierarchical phylogenetic relationships. This is accomplished by computing p-values from empirical distributions of F statistics (the ANOVA statistic) rather than from standard tabular values. The empirical distributions of F statistics are determined from simulations of the continuously valued response variable (in this case the relative evolutionary rates) along a known phylogeny using a Brownian Motion model. The assignments of species to each category remains the same during the simulations and calculation of the null distribution of F statistics.

Technically, the relative evolutionary rates are not a continuously valued phenotype that evolved along the tree, rather they are a measure of evolutionary change calculated from the multiple sequence alignments used to construct the trees. However, the branch lengths we used for the simulations are those from the average tree rather than the individual gene trees from which the relative evolutionary rates are calculated so this should reduce the problem of circularity.

PhylANOVA was run with 10,000 simulations in order to be consistent with the number of permulations performed on the RERconverge methods.

Enrichments were calculated using the Wilcoxon Rank Sum test as implemented in the RERconverge package. Enrichment permulations were not performed.

Methods Comparison

Pathway Enrichment Analysis for each Method

Pathway enrichments for sets of MGI and GO pathways (Subramanian et al., 2005; Liberzon et al., 2011) were computed for all binary RERconverge analyses, the categorical RERconverge analysis, the phylANOVA analysis, and the delta statistic analysis. For the binary and categorical RERconverge analyses, the genes were ranked by their parametric p-values. Permulations were then performed at the pathway level to correct both for non-independence among the species and for non-independence in gene rank (groups of genes in pathways often shift together in rank) (Saputra et al., 2021). The gene association results (p-values and effect sizes) from the 10,000 permulations were used to recalculate pathway enrichments for each permulation, providing 10,000 null enrichment statistics for each pathway. The permulation p-value for a pathway is the proportion of null statistics more extreme than the observed enrichment statistic. PhylANOVA does not store the intermediate F statistics of each simulation, so it was not possible to perform "phylogenetic simulations" to correct p-values at the pathway level in a way analogous to how we applied permulations to do so. Instead, the genes were ranked by their simulation p-values and the multiple hypothesis testing adjusted p-values returned directly by the pathway enrichment test were used for each pathway.

Works Cited

Louca, S., & Doebeli, M. (10 2017). Efficient comparative phylogenetics on large trees. *Bioinformatics*, *34*(6), 1053–1055. doi:10.1093/bioinformatics/btx701

Nguyen, L. T., Schmidt, H. A., von Haeseler, A., & Minh, B. Q. (2015). IQ-TREE: a fast and effective stochastic algorithm for estimating maximum-likelihood phylogenies. *Molecular biology and evolution*, *32*(1), 268–274. https://doi.org/10.1093/molbev/msu300

O'Leary, N. A., Wright, M. W., Brister, J. R., Ciufo, S., Haddad, D., McVeigh, R., Rajput, B., Robbertse, B., Smith-White, B., Ako-Adjei, D., Astashyn, A., Badretdin, A., Bao, Y., Blinkova, O., Brover, V., Chetvernin, V., Choi, J., Cox, E., Ermolaeva, O., … Pruitt, K. D. (2016). Reference sequence (RefSeq) database at NCBI: current status, taxonomic expansion, and functional annotation. *Nucleic Acids Research*, *44*(D1), D733–D745.

Supplementary Figures and Tables

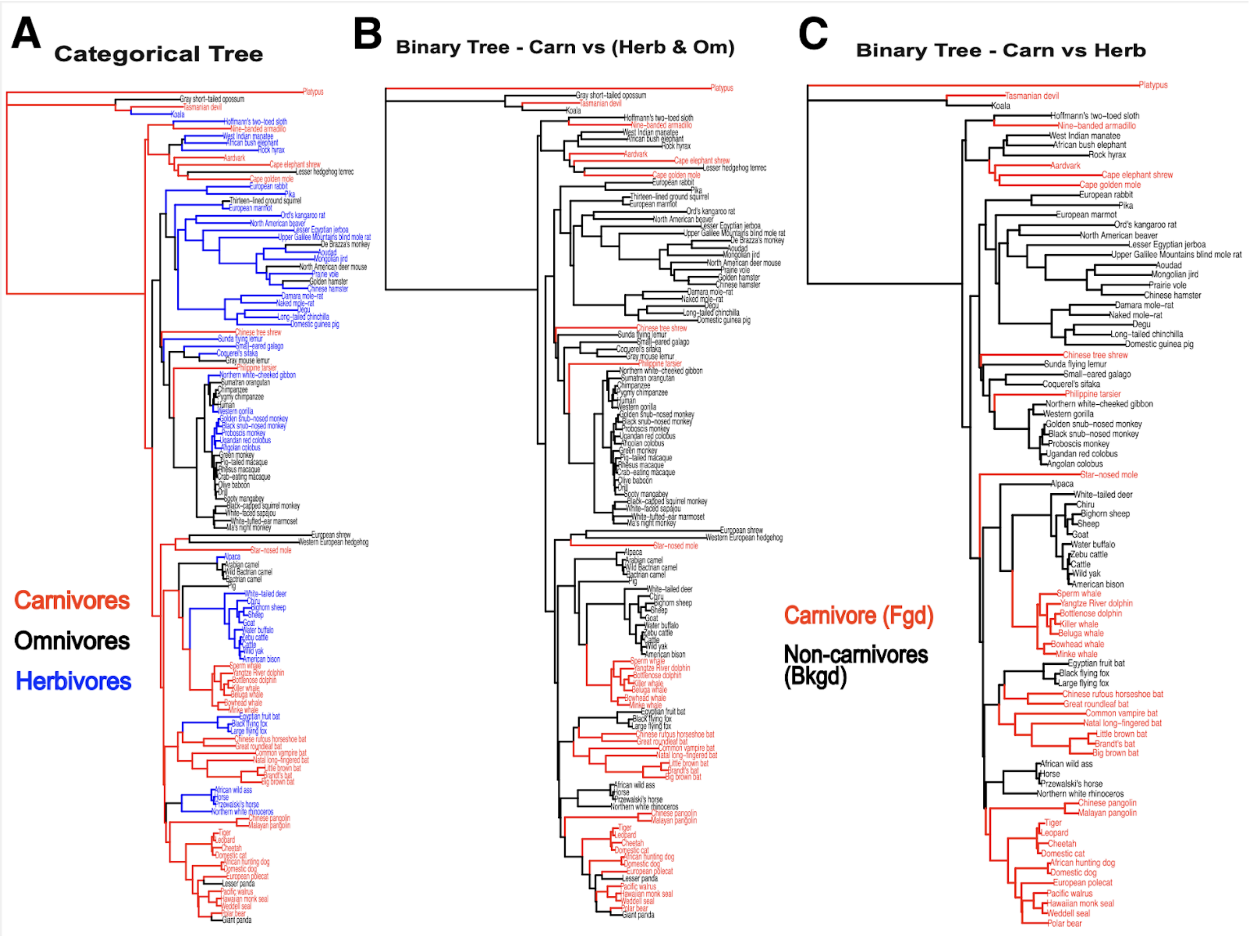

Figure S1. A) Categorical trait reconstruction using maximum likelihood applied to a continuous time markov model on the full phylogeny used in the analysis. B) Example of one of the binary trait reconstructions in which the foreground is carnivores and the background is herbivores and omnivores. Uses an approximate maximum parsimony based approach and assumes trait evolution can only occur from background to foreground. C) Example of one of the binary trait reconstructions in which the foreground is carnivore and the background is herbivores, with omnivores removed. Uses an approximate maximum parsimony based approach and assumes trait evolution can only occur from background to foreground.

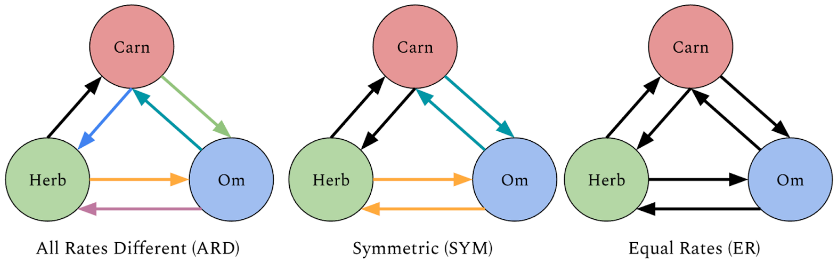

Figure S2. The circles represent the three categories (states) - carnivore (Carn), herbivore (Herb), omnivore (Om). The colored arrows represent the transition rates where arrows of the same color represent transitions with the same rate.

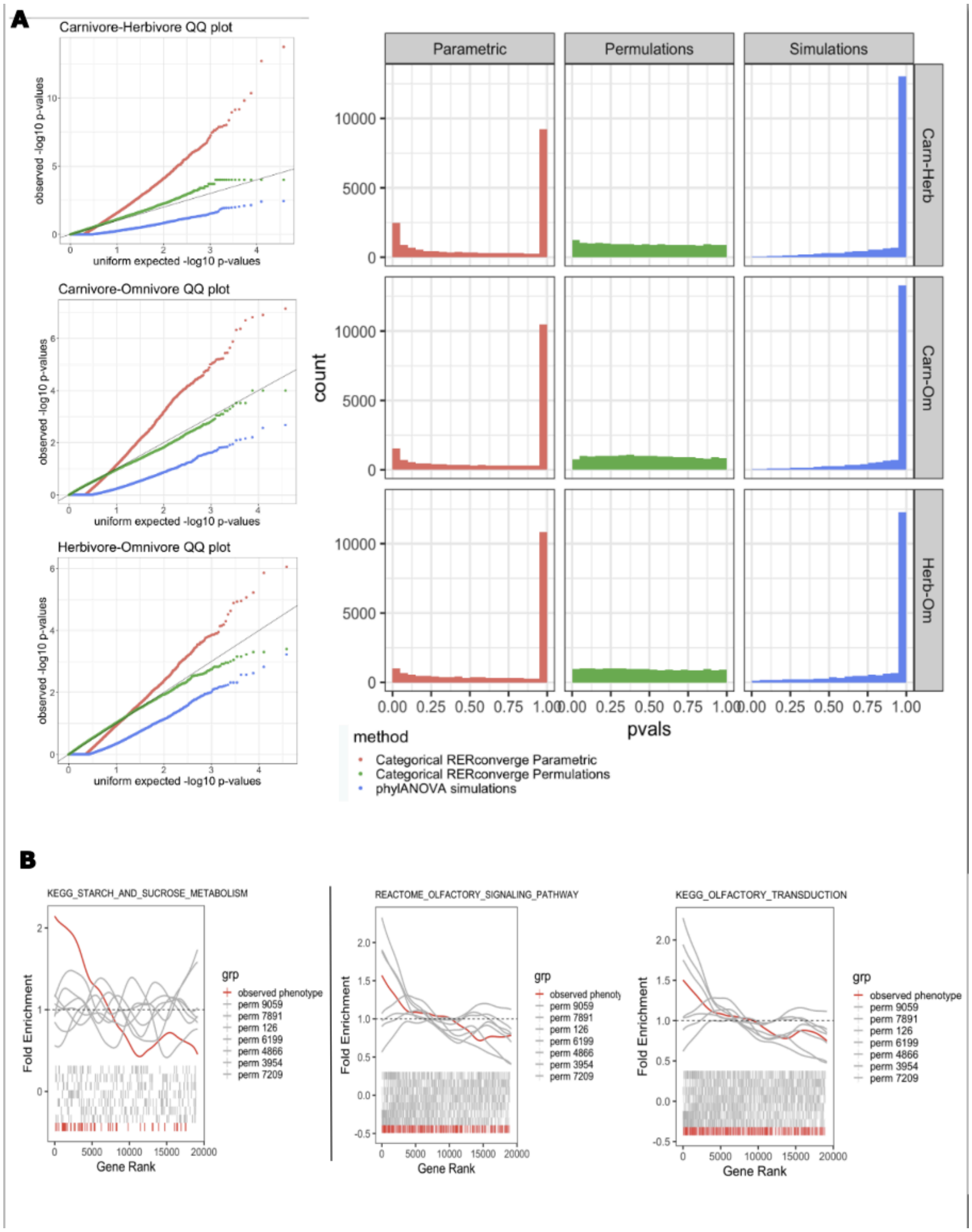

Figure S3. A) Quantile quantile plots and histograms of the categorical RERconverge raw (parametric) p-values (red) and permulation p-values (green) and the phylanova simulation p-values (blue). B) Fold enrichment and barcode plots showing the enrichment of genes in the Kegg starch and sucrose metabolism pathway (left) and two olfactory pathways (right). Red indicates the results for the observed phenotype, gray indicates the results for a random selection of seven (out of the 10,000) permulated phenotypes.

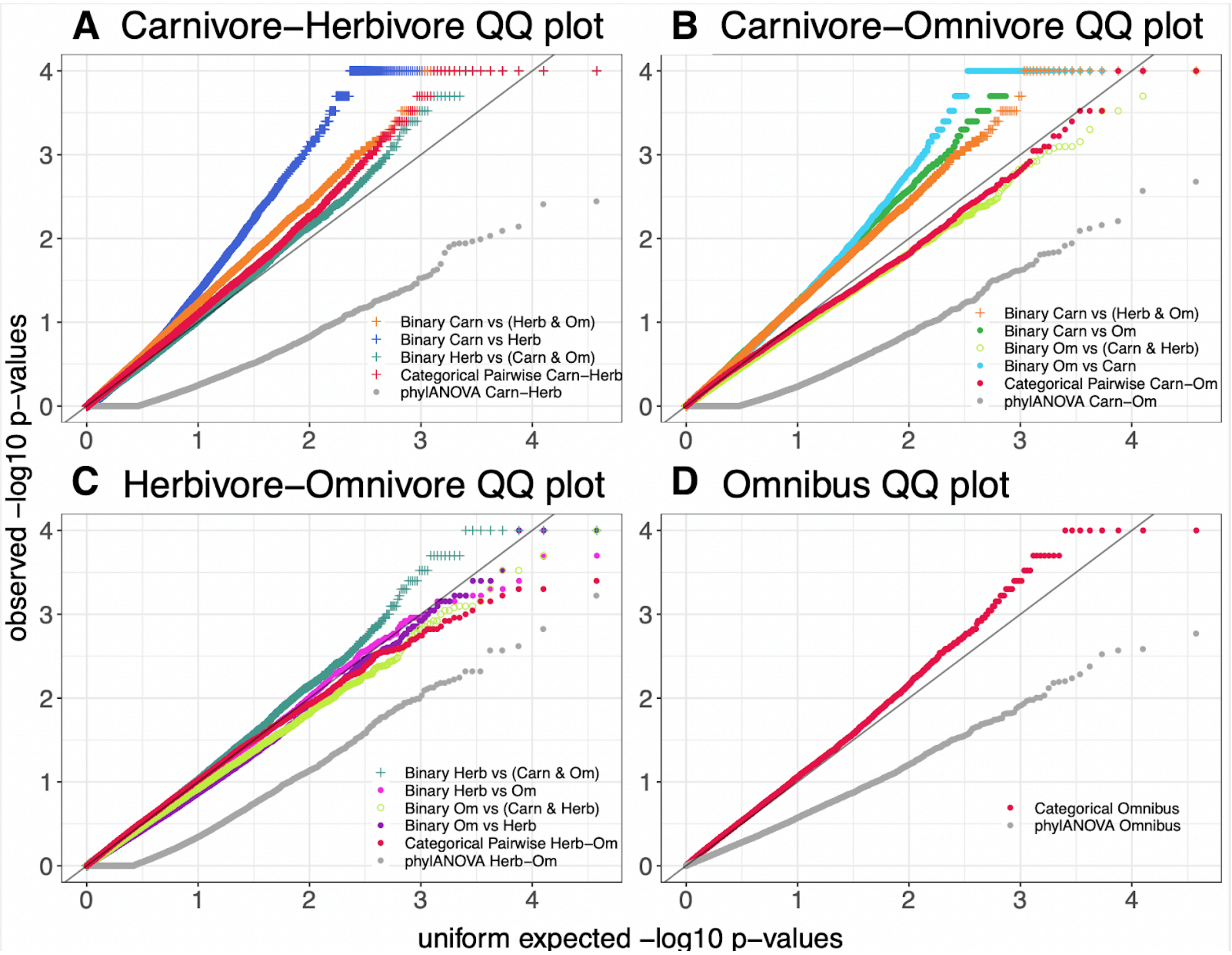

Figure S4. Quantile quantile (QQ) plots of the permulation or simulation p-values for the results of categorical RERconverge, phylANOVA, and pairwise binary RERconverge. A-C) A + symbol is used to denote any comparison in which carnivores and herbivores are separated between the foreground and background. An open circle symbol denotes the analysis in which carnivores and herbivores together form the background and omnivores form the foreground.

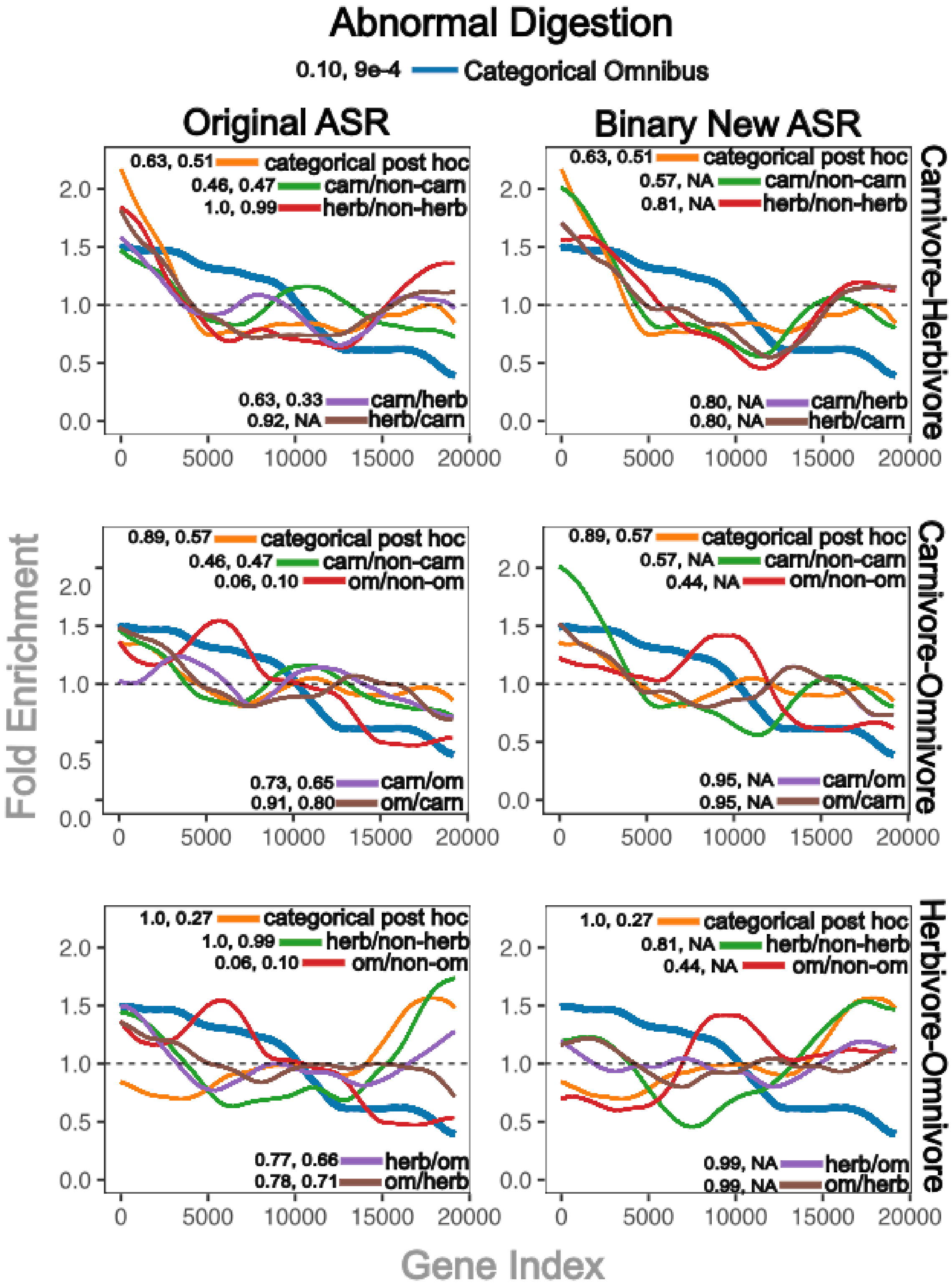

Figure S5. Fold enrichment plots of the abnormal digestion pathway. P-values are reported to the left of the method name as (adjusted p-value, permulation p-value). The permulation p-value is NA to indicate permulations were not performed for that method. Significant enrichment was determined as an adjusted p-value ≤ 0.10 and a permulation p-value ≤ 0.05.

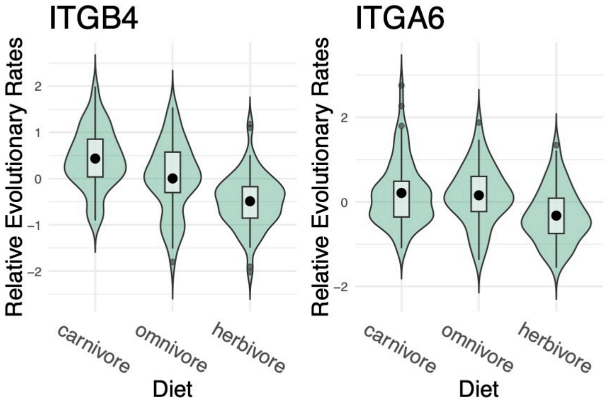

Figure S6. Violin plots of the distribution of relative evolutionary rates across each diet category for A) ITGB4 and B) ITGA6.

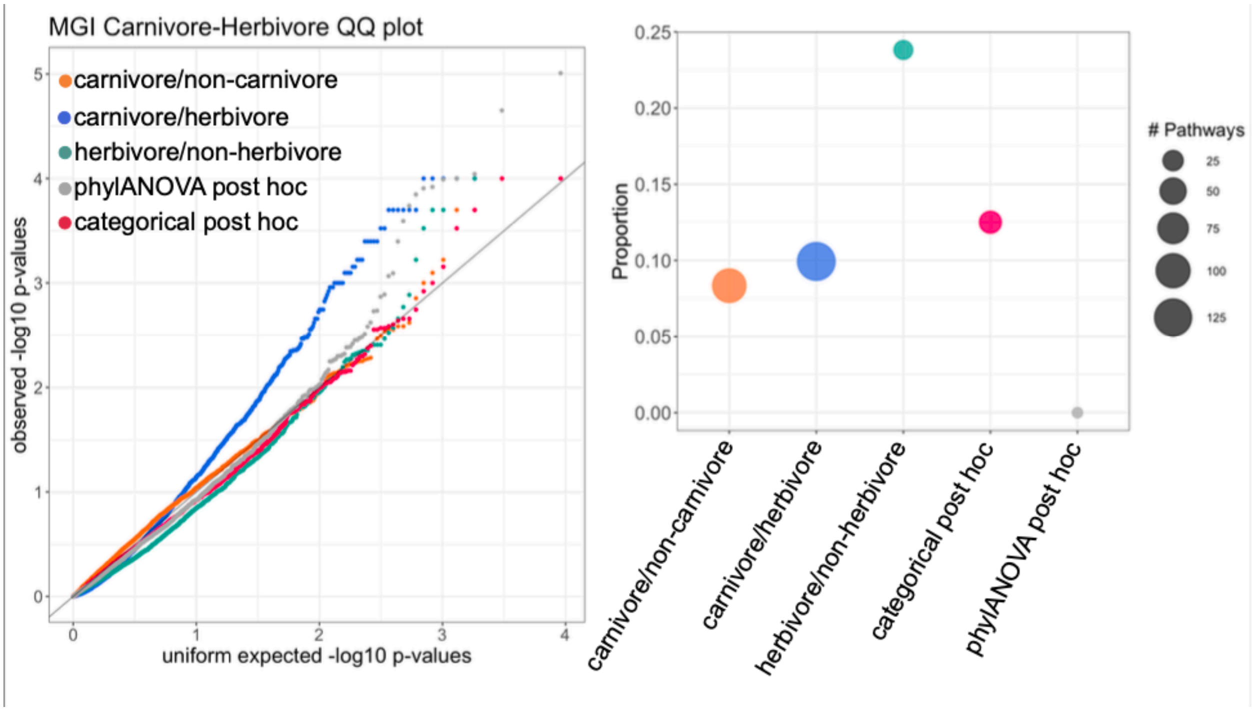

Figure S7. A) Quantile-quantile plot of the enrichment p-values (permulation p-values for the RERconverge results and adjusted p-values for the phylANOVA results). B) The proportion of top enriched pathways that are digestive system, liver/biliary system, or metabolism related. The size of the dots represents the total number of pathways for that method meeting the significance threshold of p.adj < 0.15 and, for the RERconverge methods, permpval < 0.025.

Methods Comparison Analyses

**Method**	**Comparison**	**Number of Species**	**Foreground State**	**Background State(s)**
Categorical RERconverge	omnibus	115		
	carnivore/herbivore (post hoc)			
	carnivore/omnivore (post hoc)			
	herbivore/omnivore (post hoc)			
Binary RERconverge	carnivore/non-carnivore	115	carnivore	herbivore, omnivore
	herbivore/non-herbivore	115	herbivore	carnivore, omnivore
	omnivore/non-omnivore	115	omnivore	carnivore, herbivore
	carnivore/herbivore	85	carnivore	herbivore
	herbivore/carnivore	85	herbivore	carnivore
	carnivore/omnivore	66	carnivore	omnivore
	omnivore/carnivore	66	omnivore	carnivore
	herbivore/omnivore	79	herbivore	omnivore
	omnivore/herbivore	79	omnivore	herbivore
PhylANOVA	omnibus	115		
	carnivore/herbivore (post hoc)			
	carnivore/omnivore (post hoc)			
	herbivore/omnivore (post hoc)			
Delta statistic	omnibus	115		

Table S1. Overview of each analysis performed under each method in the Methods Comparison.

Average Permulations Times

	Mean	SD	No Outlier Mean	No Outlier SD
Two Category	7.1987	0.428	7.198	0.428

Three Category	9.1044	0.951	9.1044	0.951
Four Category	38.591	34.49	32.614	18.64
Five Category	2358.0	5049.	302.38	214.1
Six Category	4933.0	NA	4933.0	NA

Table S2. Average time in seconds of all phenotype sets for each category number.

Speed Increases for Relaxing Permulations

Relaxation (%)	comparison	Mean Difference (%)	Sd Difference (%)	Outlier Mean Difference (%)	Outlier Sd Difference (%)
Single	Single	Single	Single	Single	Single
20	3v2	2.510765	3.333216	2.510765	3.333216
20	4v3	32.56813	33.64818	32.56813	33.64818
20	5v4	155.0417	196.6984	355.9884	661.9572
20	6v5	320.1345	385.5756	320.1345	385.5756
10	3v2	23.12227	13.83585	23.12227	13.83585
10	4v3	284.4025	144.2443	376.9865	395.6866
10	5v4	1501.997	762.154	4703.116	6873.465
10	6v5	1715.987	2268.198	1715.987	2268.198
5	3v2	48.57902	27.11924	48.57902	27.11924
5	4v3	1195.116	835.5254	1195.116	835.5254
5	5v4	NaN	NA	NaN	NA
5	6v5	NaN	NA	NaN	NA
0	3v2	51.14266	30.8599	51.14266	30.8599
0	4v3	1591.495	1627.517	1591.495	1627.517
0	5v4	NaN	NA	NaN	NA
0	6v5	NaN	NA	NaN	NA
Double	Double	Double	Double	Double	Double

20	4v2	32.80652	NA	32.80652	NA
20	5v3	163.9363	88.87492	163.9363	88.87492
20	6v4	1028.2	NA	1028.2	NA
10	4v2	481.8241	NA	481.8241	NA
10	5v3	5014.968	3484.625	5014.968	3484.625
10	6v4	32220.14	NA	32220.14	NA
5	4v2	1947.827	NA	1947.827	NA
5	5v3	NaN	NA	NaN	NA
5	6v4	NaN	NA	NaN	NA
0	4v2	1173.703	NA	1173.703	NA
0	5v3	NaN	NA	NaN	NA
0	6v4	NaN	NA	NaN	NA

Table S3. Percent speed increases for reducing the number of categories in the analysis, at each relaxation level.

Permulations Times and Speed Increases at each Relaxation Level

	Mean_0	Sd_0	Mean_5	Sd_5	%Dif_5	Mean_10	Sd_10	%Dif_10	Mean_20	Sd_20	%Dif_20
twoCategory	8.11	0.543	8.007	0.549	1.370	7.198	0.428	12.76	8.243	0.4890	-1.53157
threeCategory	14.35389	4.1969	13.883	5.0644	3.3853	9.1044	0.951	57.657	8.8042	0.5050	63.033
fourCategory	243.393	112.52	205.41	162.61	18.486	32.614	18.64	646.26	11.6372	2.3934	1991.49
fiveCategory	4320+	NA	4320+	NA	NA	302.38	214.1	NA	28.408	23.735	NA
sixCategory	4320+	NA	4320+	NA	NA	4933.0	NA	NA	119.679	NA	NA
twoCategoryAll	8.11748	0.543	8.0077	0.5498	1.3704	7.1987	0.428	12.762	8.2437	0.4890	-1.5315
threeCategoryAll	14.3538	4.1969	13.883	5.0644	3.3853	9.1044	0.951	57.657	8.8042	0.5050	63.033
fourCategoryAll	276.698	196.8	205.41	162.61	34.70	38.591	34.49	616.98	11.6372	2.3934	2277.6
fiveCategoryAll	4320+	NA	4320+	NA	NA	2358.0	5049.	NA	61.5213	96.201	NA
sixCategoryAll	4320+	NA	4320+	NA	NA	4933.0	NA	NA	119.679	NA	NA

Table S4. Table of the time average and standard deviations of time required to complete a phenotype set with a given number of categories, at each relaxation level tested. %Dif column represents the percentage speed increase of the relaxation level over 0% relaxation. As times for 0% and 5% relaxation for could not be completed, times were very conservatively estimated as the minimum time required to reach the run time produced (see methods), and no %Dif could be created.

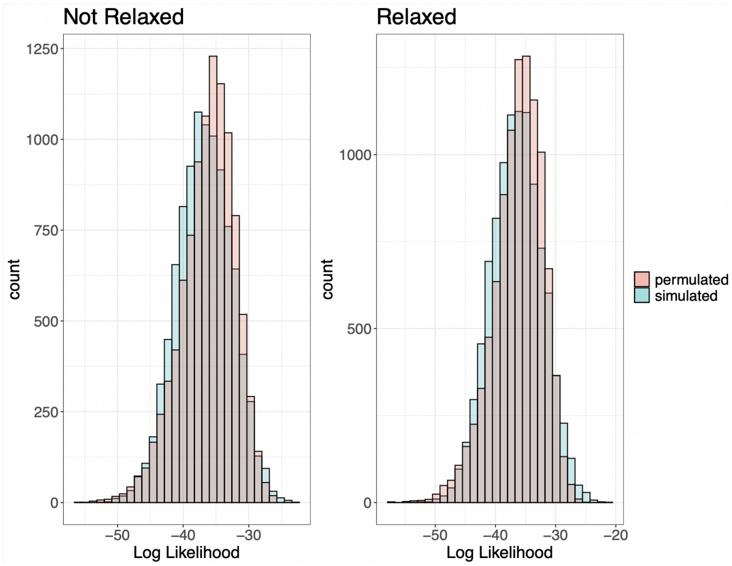

Figure S8. Histograms of the log likelihoods of the 10,000 simulated trees (blue) compared to the log likelihoods of the finished permulated trees (pink) for permulations without relaxation (left) and permulations with relaxation (right).

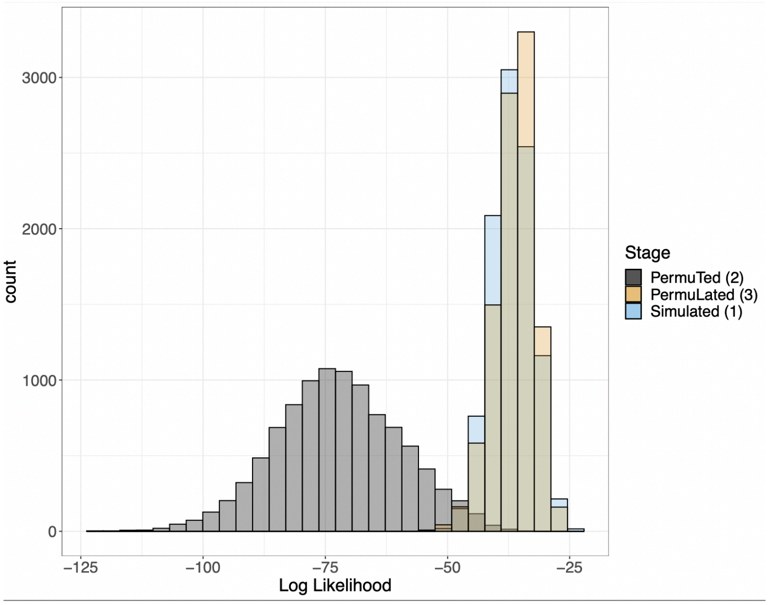

Figure S9. In blue are the log likelihoods of the 10,000 simulated trees (after step 1), in gray are the log likelihoods of the 10,000 permuted trees (after step 2), and in tan are the log likelihoods of the 10,000 finished permulated trees (after step 3).

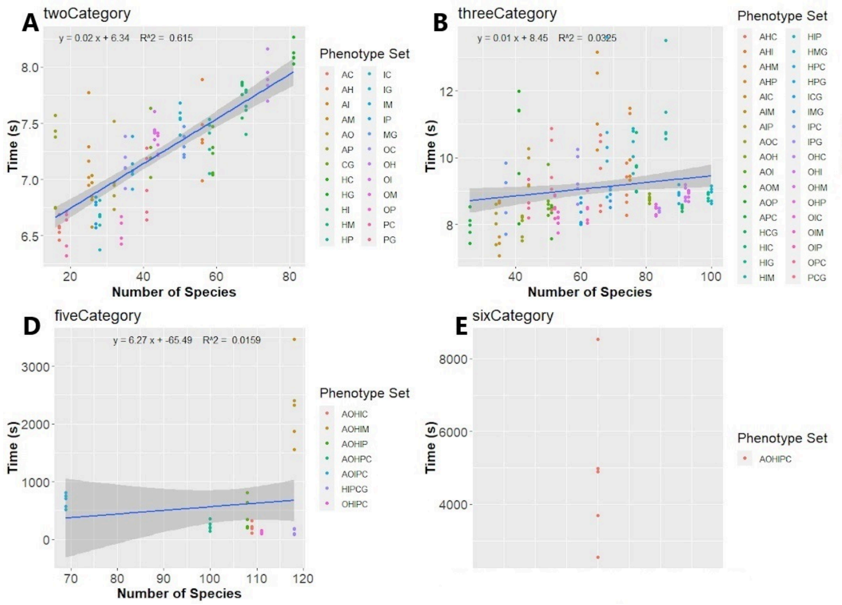

Figure S10. Linear regressions of time to complete permulations against number of species included in permulations. For two category analyses (A), species number has an effect, but it is minor (0.02 seconds per species). For analyses with three or more categories (B - E), there does not appear to be an effect. Note the differences in y-axis scale between category numbers.

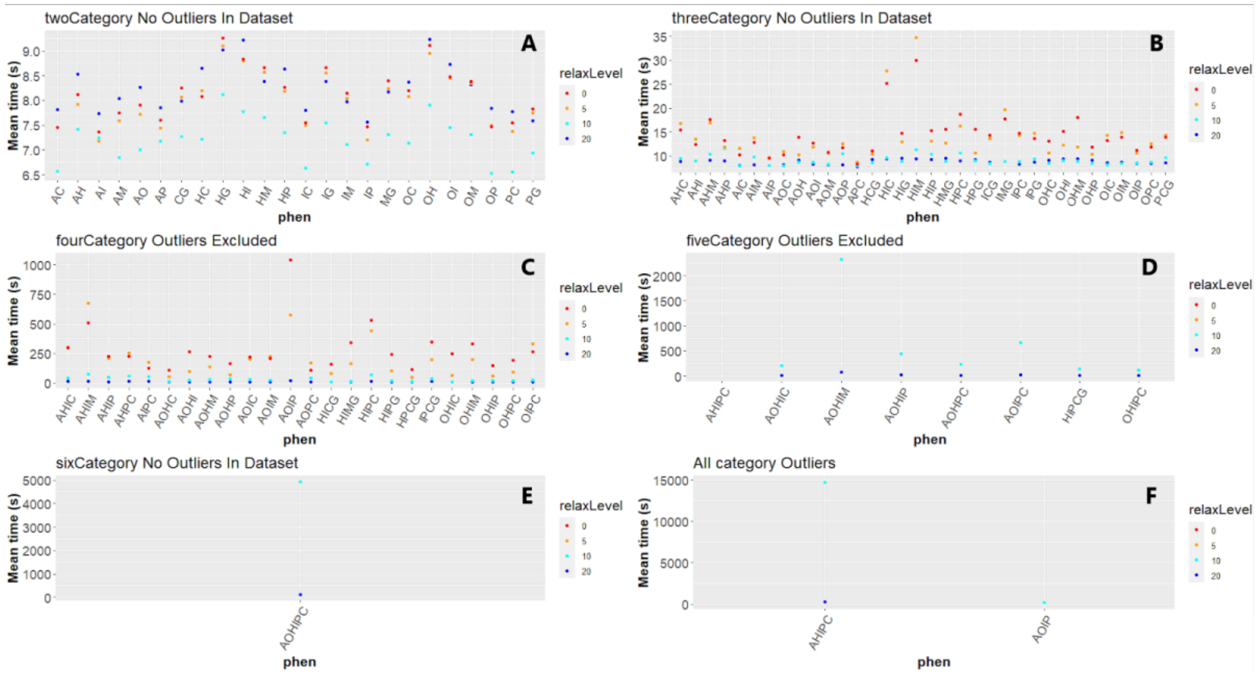

Figure S11. Plots of average time in seconds required to complete each phenotype set, arranged by category number (A,B,C,D,E). Note the difference in y-axis scales between categories.

Outliers are present in a separate plot (F) to allow for readable scales. Each point represents the average of five time trials of the phenotype, with the color of the point indicating the relaxation level of the time trials.

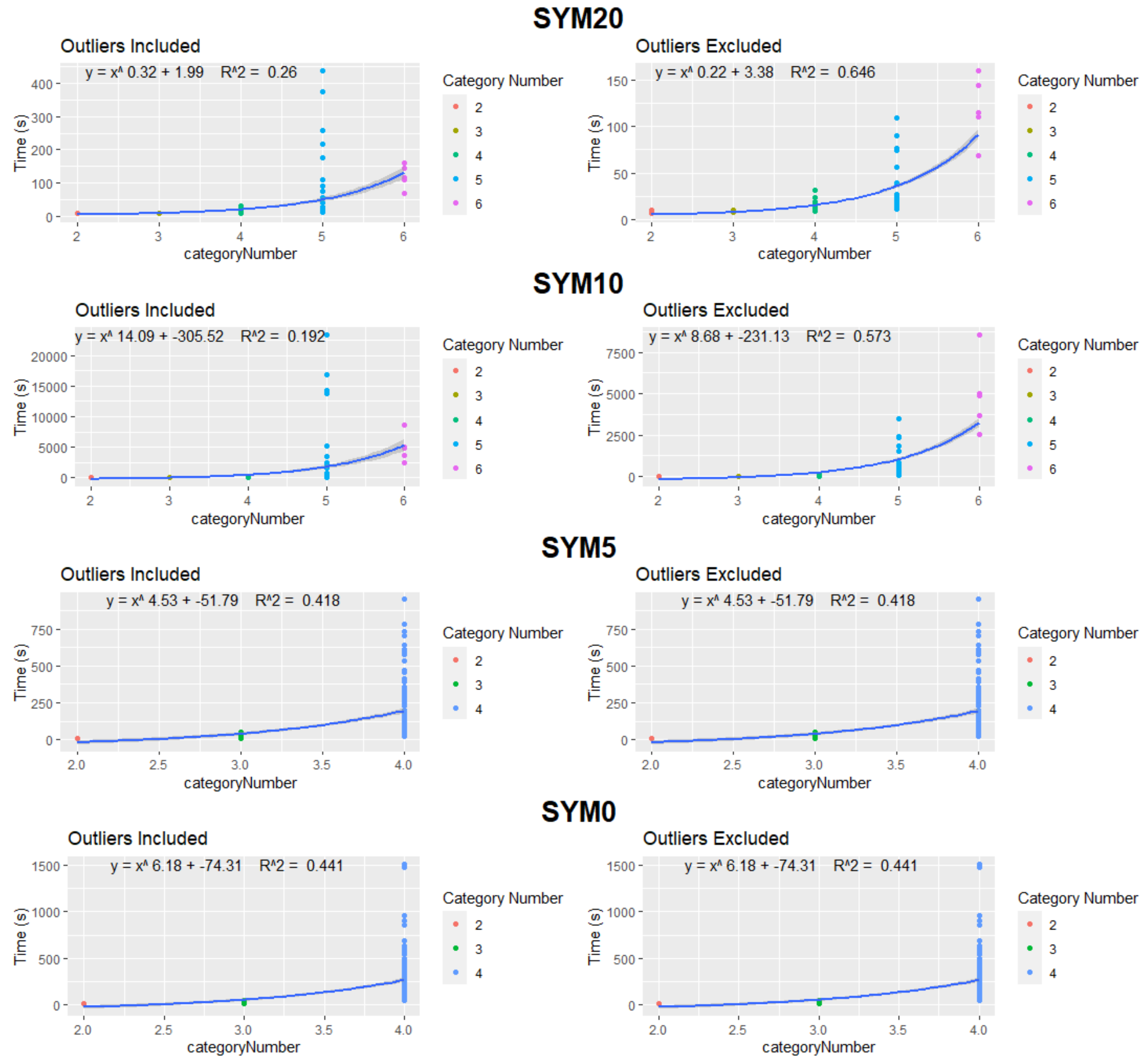

Figure S12. Graphs of the exponential effect of category number on permulations time. Note that the 5% and 0% relaxation graphs do not include 5 category or 6 category analyses.

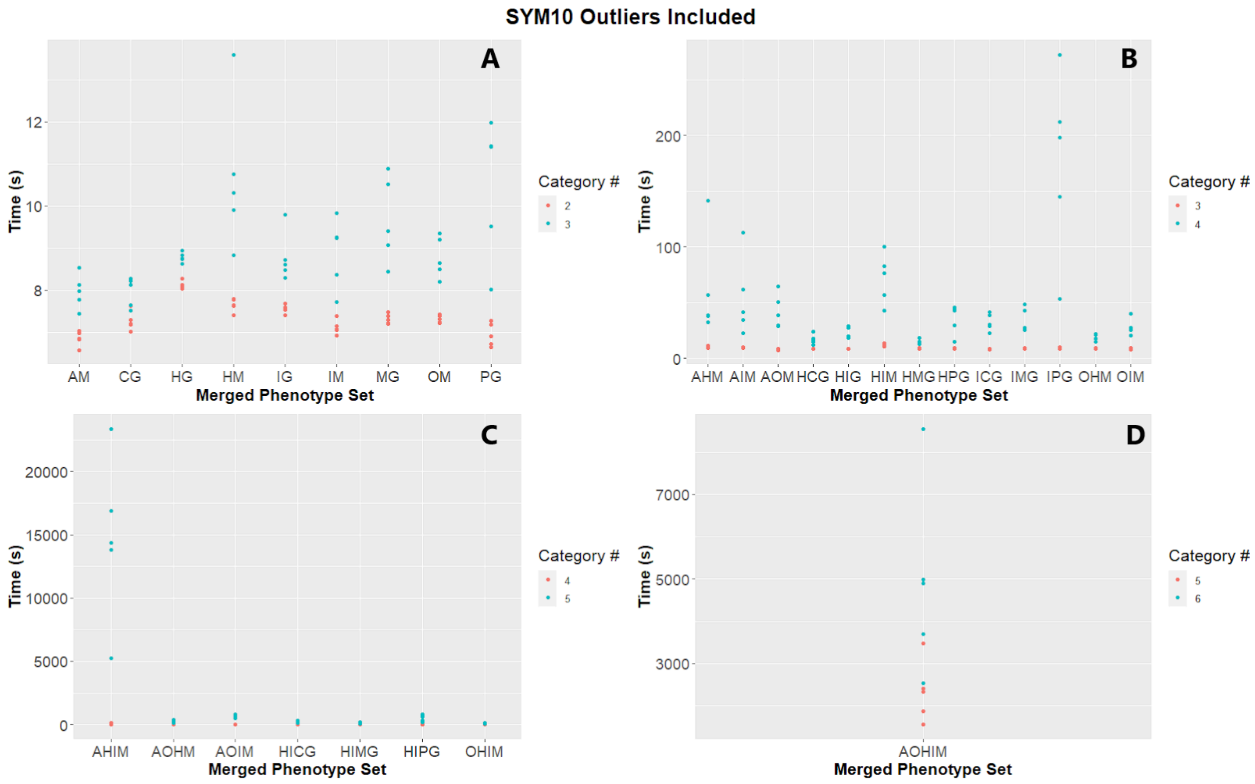

Figure S13. Plots of runtime in seconds of all singly-combinable phenotypes; when combined two (A), three (B), four (C), and five (D) categories, showing both merged and unmerged times. Unmerged phenotypes sets (eg. Herbivore-Carnivore-Piscivore) are shown in blue, merged phenotype sets (eg. Herbivore-<Carnivore&Piscivore>) are shown in red. The X-axis displays the first letter of the phenotypes used in the merged phenotype set.
