# Supplementary material for: RERconverge Expansion: Using Relative Evolutionary Rates to Study Complex Categorical Trait Evolution
Source: Mol Biol Evol. 2024 Oct 15;41(11):msae210. doi: 10.1093/molbev/msae210 (PMC11529301; doi:10.1093/molbev/msae210)
Supplement: msae210_Supplementary_Data [file msae210_supplementary_data.zip › vignettes.pdf]

# Categorical Trait Analysis Walkthrough

July 05, 2023

## Contents

**Overview** **2**
Data Input Requirements and Formatting . . . 2

**Analysis Walkthrough** **2**
Installation . . . 2
Inferring the Phenotype Tree . . . 3
Generating **paths** using `char2PathsCategorical` . . . 5
Visualization . . . 6
Correlating gene evolution with categorical trait . . . 8

**Enrichment Walkthrough** **10**

**Conclusion** **11**

This walkthrough describes how to use the updates to RERconverge for analyzing categorical traits. This update builds on existing RERconverge objects. First time users should first read the "RERconverge Analysis Walkthrough" vignette.

# Overview

The following document illustrates how to perform a categorical trait analysis after relative evolutionary rates have been calculated. To learn how to calculate relative evolutionary rates using `getAllResiduals` follow the "RERconverge Analysis Walkthrough" vignette.

**Output** is a list of two data objects. The first is a data frame containing a list of genomic elements with association statistics between the genomic element's evolutionary rate and the phenotype. The second object is a list of data frames for each pairwise test between the phenotype categories. For n phenotype categories, there will be $\binom{n}{2}$ data frames in this list. Each data frame is a list of genomic elements with association statistics describing the difference in relative evolutionary rates of genomic elements between the two categories.

## Data Input Requirements and Formatting

The required inputs are as follows:

1. Phylogenetic trees of the same format described in the "RERconverge Analysis Walkthrough" vignette.

2. Species-labeled phenotype values

- The species labels MUST match the tree tip labels that were used in `getAllResiduals` to calculate the relative evolutionary rates (RERs)
- a named numeric vector of categorical trait values

# Analysis Walkthrough

## Installation

Follow the steps for installing RERconverge on the wiki, up to the "Install from Github" step. Then, load the RERconverge library.

```
if (!require("RERconverge", character.only = T, quietly = T)) {
  require(devtools)
  install_github("nclark-lab/RERconverge", ref = "master")
  # ref refers to the branch being installed
}
library(RERconverge)
```

Follow the instructions in the "RERconverge Analysis Walkthrough" vignette in order to read in gene trees using `readTrees` and calculate evolutionary rates using `getAllResiduals`. That vignette describes how to save the RER object for later using `saveRDS`. Save both the RER object and the trees object. Make sure that you save these objects into your working directory for your project. We will read them in using `readRDS`. We will also read in the phenotype data.

It is very important that the names of the phenotype data EXACTLY match the names of the species that were used to calculate the relative evolutionary rates in `getAllResiduals`. To ensure this is the case, follow the instructions in the "RERconverge Analysis Walkthrough" vignette.

To use your own data that has already been saved in your working directory use the following code to read in the .rds files replacing the names of the files here with the names of your files:

```
# read in the trees
toyTrees =readRDS("toyTrees.rds")

# read in the phenotype data
basalRate = readRDS("basalRate.rds")

# read in the RERs
RERmat = readRDS("bodyTempRERs.rds")
```

To use the same data as in this walk through, run the following code to read in the trees, load in the phenotype data, and calculate the relative evolutionary rates.

```
# find where the package is located on your machine
rerpath = find.package('RERconverge')

# read in the trees with the given file name
toytreefile = "subsetMammalGeneTrees.txt"
toyTrees=readTrees(paste(rerpath,"/extdata/",toytreefile,sep=""), max.read = 200)

# load the phenotype data into your workspace
# This will create a named vector with the name basalRate
data("basalRate")

# calculate the relative evolutionary rates with getAllResiduals
RERmat = getAllResiduals(toyTrees, useSpecies = names(basalRate))
```

## Inferring the Phenotype Tree

Next, we generate the phenotype tree from the species-labeled phenotype vector using `char2TreeCategorical`.

This function uses code from castor(Louca and Doebeli 2017) and internally calls the function `getAncLiks` which borrows heavily from the function `ace` in ape(Paradis and Schliep 2019) and from the functions `fitMk` and `rerootingMethod` in phytools(Revell 2012). The relevant citations are given below:

For more information on choosing a rate model (see parameter `model` below) refer to the "Ancestral State Reconstruction for Binary and Categorical Traits" vignette.

This function takes the following inputs:

- `tipvals`: The named vector of phenotype data. This may be categorical data or binary data. If the binary phenotype data is of type logical (TRUE/FALSE), `char2TreeCategorical` automatically returns the inferred ancestral states as a binary tree with branch lengths of 1 and 0.
- `treesObj`: The trees object containing every gene tree
- `useSpecies`: Specifies the subset of species to use in the analysis. This vector of species should match the subset used to calculate RERs.
- `model`: A character or matrix describing the model used for fitting the transition rate matrix. The default option is `"ER"`, short for equal rates indicating that all transitions between states occur at the same rate. `"SYM"` (symmetric) and `"ARD"` (all rates different) are also options. For more information, reference the ace documentation in ape or the "Ancestral State Reconstruction for Binary and Categorical Traits" vignette, which provides an in depth tutorial for selecting a rate model.
- `plot`: A boolean specifying whether to plot the phenotype tree.
- `anctrait`: If provided, the states of ancestral species will be assigned to this trait rather than being inferred using maximum likelihood methods. If provided, it MUST be one of the traits in the phenotype vector. The default value is `NULL`.

The code below shows how to obtain a phenotype tree using `char2TreeCategorical`.

```r
allspecs = names(basalRate)

# unrooted
phenTree = char2TreeCategorical(basalRate, toyTrees, useSpecies = allspecs, model = "ER",
                                plot = TRUE)
```

```
## Species from master tree not present in useSpecies: Platypus,Opossum,Tasmanian_devil,Wallaby,Armadil

## [1] "The integer labels corresponding to each category are:"
## high  low  med
##    1    2    3
```

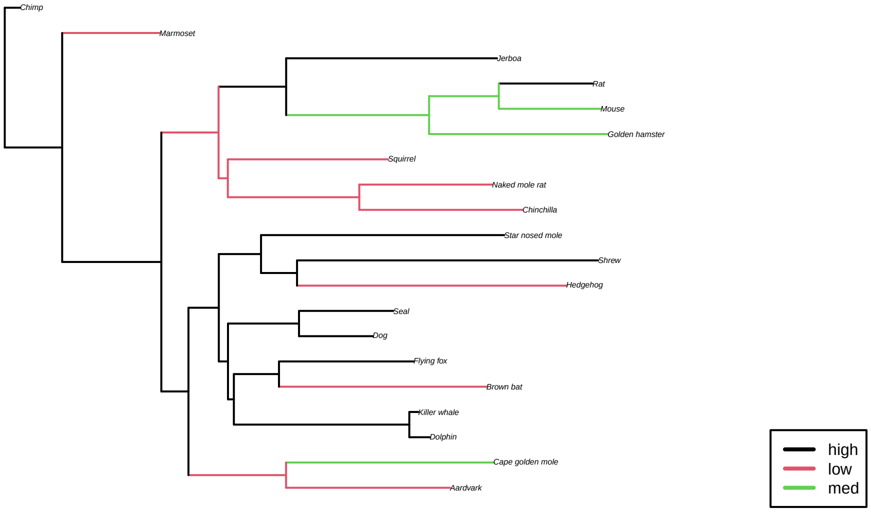


By default, `char2TreeCategorical` infers the ancestral states of species in the tree such that internal species can be assigned to any of the categories. However, there may be phenotypes in which it makes more sense for all ancestral species to belong to one category with some of the extant species belonging to the remaining categories. This is analogous to using the `clade = terminal` option in `foreground2Tree` for binary traits. (See the "RERconverge Analysis Walkthrough vignette for a more detailed discussion of the `clade` argument.)

By providing an ancestral trait via the `anctrait` argument, `char2TreeCategorical` will not infer the ancestral states of species. Instead, all ancestral species will be assigned to the ancestral trait while the extant species will be assigned to categories according to the named phenotype vector. The ancestral trait must be one of the traits in the phenotype vector, thus it is important to ensure that it is spelled the same and contains the same pattern of capitalization. When using this option with a trait that contains only two categories, the output will be a binary phenotype tree (the same output as `foreground2Tree` with `clade = terminal`), and the function will prompt you to proceed as appropriate for a binary trait analysis.

```r
allspecs = names(basalRate)

phenTree = char2TreeCategorical(basalRate, toyTrees, useSpecies = allspecs, plot = TRUE,
                                anctrait = "high")
```

The output of `char2TreeCategorical` is a phenotype tree with the same topology as the master tree. The phenotype states are stored on the branches of the tree as edge lengths. The ancestral trait reconstruction functions use numerical (integer) tip labels. The integers do not have any biological significance and are typically assigned in alphabetical order. The integer values corresponding to the categories in the phenotype vector are printed to the console.

Additionally, to see the mapping of category names to integers, you can run the castor function that is used within `char2TreeCategorical` as shown below:

```r
intlabels = map_to_state_space(basalRate)
print(intlabels$name2index)
```

```
## high  low  med
##    1    2    3
```

**Note:** `char2TreeCategorical` can automatically detect a binary phenotype and return the inferred phenotype tree as a binary tree (with branch lengths of 1 and 0). This binary tree is not necessarily equivalent to the binary tree returned by `foreground2Tree` because it uses different methods for inferring ancestral states.

#### Interactive Phenotype Tree Construction

Alternatively, RERconverge provides an interactive selection tool. To open the interactive selection tool run `click_select_foreground_branches` as shown below. Running this function should open a plot of the master tree.

```r
phenTree = click_select_foreground_branches(toyTrees$masterTree)
```

Before making any selections, all the branches are automatically assigned to category 1. Begin by selecting branches corresponding to category 2. To select branches for the next category click the "New Category" button and select the corresponding branches. These will be assigned to category 3 and so on.

IMPORTANT: When using this approach, there is no method that maps category names to numbers. Please keep track of the order in which you add the categories as `map_to_state_space` will NOT give the correct mapping. When finished, click "End Selection". If you only select foreground branches for one category, this function will automatically return a binary phenotype tree in which the branch lengths are "1"s and "0"s, and it will prompt you to use `correlateWithBinaryPhenotype` instead. In this case, you should also generate paths as described in the "RERconverge Analysis Walkthrough" vignette for binary traits.

### Generating paths using `char2PathsCategorical`

The `toyTrees` object contains a separate gene tree for each gene in the analysis with branch lengths representing the evolutionary rates of that gene. All of the gene trees have the same overall topology as the master tree, but some of them are missing certain species. To handle missing species, RERconverge generates something called paths. For a more detailed discussion of paths see the "RERconverge Analysis Walkthrough" vignette. For categorical traits we use the function `char2PathsCategorical`. This function has the same inputs as `char2TreeCategorical`.

```r
allspecs = names(basalRate)

# unrooted
charP = char2PathsCategorical(basalRate, toyTrees, useSpecies = allspecs, model = "ER",
                              plot = FALSE)
```

```
## Species from master tree not present in useSpecies: Platypus,Opossum,Tasmanian_devil,Wallaby,Armadill

## [1] "The integer labels corresponding to each category are:"
## high  low  med
##    1    2    3
```

Alternatively, you can generate paths directly from a phenotype tree generated by `char2TreeCategorical` or `click_select_foreground_branches` using the function `tree2Paths`.

IMPORTANT: The phenotype tree MUST have the same topology as the master tree or a subset of the master tree. This is always the case when using `click_select_foreground_branches` or `char2TreeCategorical`.

```r
charP = tree2Paths(phenTree, toyTrees, categorical = TRUE)
```

(To see how to use `tree2Paths` on binary trees reference the "RERconverge Analysis Walkthrough" vignette.)

## Visualization

**Visualizing the phenotype tree:** In addition to visualizing the phenotype tree with `char2TreeCategorical` and `char2PathsCategorical` when `plot = TRUE`, the tree can be plotted with `plotTreeCategorical`. The inputs of this functiona are:

- `tree`: The phenotype tree returned by `char2TreeCategorical`
- `category_names`: If provided, the plot includes a legend with the category names and corresponding colors. The category names MUST be provided in the same numerical order as the mapping from names to integers. This can be done easily using the `intlabels` object returned by `map_to_state_space`. `category_names` can be set to `intlabels$state_names`. Otherwise the default value is `NULL` and no legend is included.
- `master`: The master tree in the trees object returned by `readTrees`. This tree will be plotted with its branches colored by the phenotypes stored in the phenotype tree.
- `node_states`: The states at each node (in order of the nodes in the tree). If provided, these are used to color the vertical bars corresponding to each node in the tree. The default is `NULL`.

```
# get the states from the edges of the phenotype tree
root_state = 1 # high (1) is the state at the root
states = getStatesFromPhenTree(phenTree, root_state, intlabels$mapped_states)

# plotting phenotypes without relative evolutionary rates represented by branch length
plotTreeCategorical(tree = phenTree, category_names = intlabels$state_names,
                    node_states = states)
```

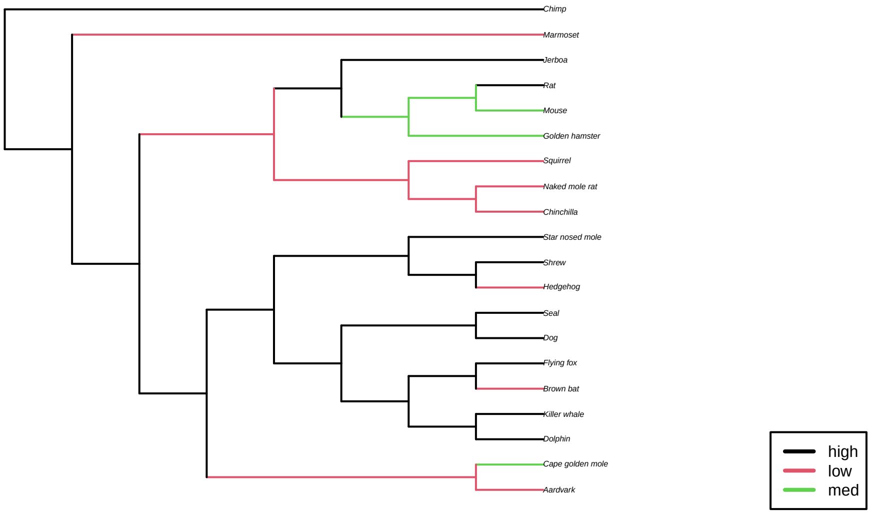


```
# plotting phenotypes with branch lengths representing evolutionary rates from the master tree
plotTreeCategorical(tree = phenTree, category_names = intlabels$state_names,
                    master = toyTrees$masterTree, node_states = states)
```

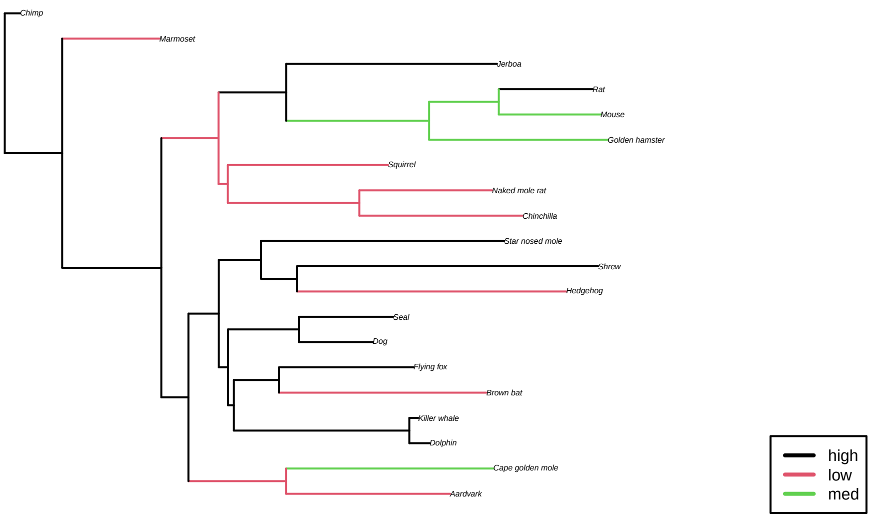


**Visualizing the relative evolutionary rates:** The relative evolutionary rates for a specific gene can be visualized using the function `plotRers`. For more details regarding reading this plot, see the "RERconverge Analysis Walkthrough" vignette. A negative value indicates a relative evolutionary rate that is below average while a positive value indicates a relative evolutionary rate that is above average. The colors used to distinguish categories match the colors in the phenotype tree that is plotted by `plotTreeCategorical`, `char2TreeCategorical`, or `char2PathsCategorical`.

The example below will show how to plot the RERs for one of the top genes, `"AP5M1"`. The `plotRers` function takes the following as input:

- `RERmat`: The RER matrix returned by `getAllResiduals`.
- `gene`: Either the name of the gene or the numerical index of the gene in the RER matrix.
- `phenv`: The paths generated by `char2PathsCategorical`.

The default method for calculating the correlation statistics that are displayed at the top of the plot is `"kw"` for Kruskal Wallis. To use ANOVA, use the parameter `method = "aov"`.

```
gene = "AP5M1"
plotRers(RERmat, gene, phenv = charP)
```

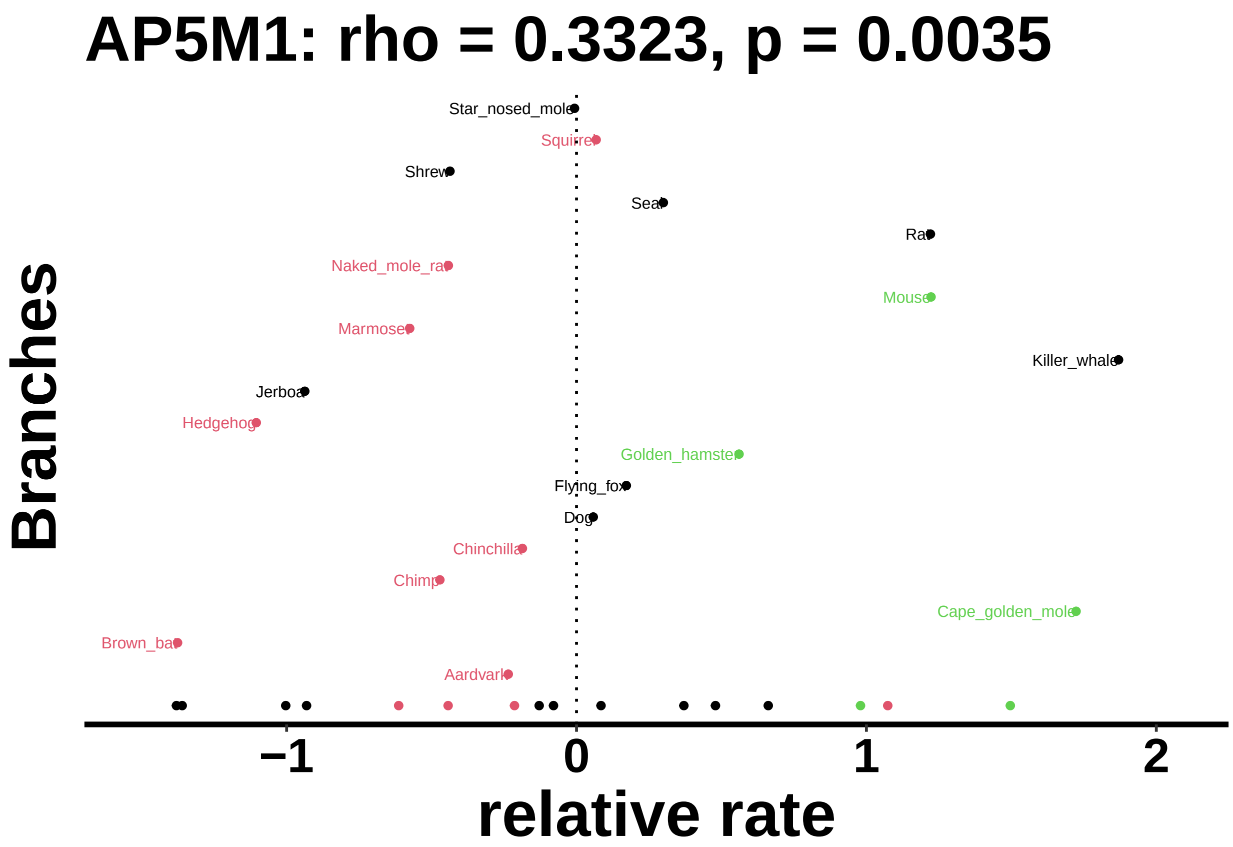


### Correlating gene evolution with categorical trait

To correlate gene evolution with categorical trait evolution we use the function `correlateWithCategoricalPhenotype`. The function tests for association between the relative evolutionary rates of genes and the evolution of the phenotype. It takes the following as input:

- `RERmat`: The RER matrix from `getAllResiduals`.
- `charP`: The paths vector from `char2PathsCategorical`.
- `min.sp`: The minimum number of species in the gene tree in order for that tree to be included in the analysis. The default value is 10.
- `min.pos`: The minimum number of species in each category in the gene tree in order for that gene to be included in the analysis. The default value is 2.
- `method`: The input options are `"kw"` for performing a Kruskal-Wallis test (the non parametric option). The pairwise testing is done using a Dunn Test. To use an ANOVA test instead (the parametric option), use `method = "aov"`. In this case the pairwise testing is done using a Tukey Test. When not specified, the default is `"kw"`.

The function for performing the Dunn Test comes from the package FSA(Ogle et al. 2022).

```
# KW/Dunn (default)
cors = correlateWithCategoricalPhenotype(RERmat, charP)
```

```
# ANOVA/Tukey
cors = correlateWithCategoricalPhenotype(RERmat, charP, method = "aov")
```

The output (`cors`) is the two-element list described previously in the above section. The first element of cors is a table with the following output for each gene: Rho, N, P, and p.adj. Descriptions of each are given in the "RERconverge Analysis Walkthrough" vignette. However, Rho is not just the test statistic, rather it is the

effect size. The Kruskal Wallis test uses epsilon squared as the measure of effect size. The epsilon squared effect size for the Kruskal Wallis test is calculated by taking the H statistic and dividing it by the number of observations minus one as described in this article. For the ANOVA test, eta squared is used as the effect size. Eta squared is calculated according to the method in this article.

Extract the first element of `cors` as follows:

```
allresults = cors[[1]]

# view the first few results
head(allresults[order(allresults$P),])
```

```
##               Rho  N           P    p.adj
## AP5M1  0.3323218 35 0.003519378 0.363086
## BRAF   0.3700350 28 0.006768521 0.363086
## ADAD1  0.2885543 35 0.007406307 0.363086
## BRSK2  0.3040665 31 0.010451633 0.363086
## ACTL7B 0.2738857 34 0.010898678 0.363086
## ARSA   0.3112253 29 0.012814799 0.363086
```

The second element in the cors object is a list of tables from the pairwise analysis. Extract this list as follows:

```
pairwise_tables = cors[[2]]
```

Run `names(pairwise_tables)` to see the order of the pairwise comparisons in this list. They are labeled numerically so, for example, the element named `1 - 3` is the data frame with the results of the pairwise comparison between the category mapped to the number `1` and the category mapped to the number `3`.

Recall that the mapping of category names to integers was printed to the console when `char2TreeCategorical` or `char2PathCategorical` was run. Additionally, recall that you can view the mapping using functions from the castor library as shown below:

```
intlabels = map_to_state_space(basalRate)
print(intlabels$name2index)
```

Each data frame in the list `pairwise_tables` contains the following output for each gene:

1. Rho: Though the column is labeled Rho (in order to stay consistent), this is the test statistic returned from either the Dunn test or the Tukey test. For the Dunn test it is known as the Z statistic and for the Tukey test it is the Honest Significant Difference. It represents the relationship between the relative evolutionary rate of a gene and evolution of the phenotype.

2. P: The p-value corrected for pairwise testing, but not corrected for multiple hypothesis testing for the many genes.

3. p.adj: The p-value corrected for multiple hypothesis testing using the Benjamini & Hochberg method.

```
# View the top results of the third pairwise comparison
table = pairwise_tables[[3]]
head(table[order(table$P),])
```

```
##               Rho           P     p.adj
## AP5M1  -3.361228 0.002327904 0.3887599
## ADAD1   3.132053 0.005207653 0.4348390
## ACTL7B  2.999228 0.008119932 0.4520095
## BRSK2   2.840025 0.013532984 0.5650021
## BIRC5  -2.682493 0.021922723 0.6003820
## ABLIM2  2.664433 0.023135501 0.6003820
```

# Enrichment Walkthrough

The enrichment analysis is performed in the same way as for binary and continuous traits.

You will need to download the gene sets and gene symbols from GSEA-MSigDB as gmtfile.gmt. Follow the instructions in the "RERconverge Analysis Walkthrough" vignette in order to properly download and save the gmt file in your current working directory. The "RERconverge Analysis Walkthrough" may say to download the file named c2.all.v6.2.symbols.gmt, however if that is not available, c2.all.v7.5.1.symbols.gmt will work. Ensure that the name of the gmt file in your working directory is "gmtfile.gmt".

**Input** is the output from the correlation function. This can be `allresults` (`cors[[1]]`) or any of the tables in the list, `pairwise_tables` (`cors[[2]]`). The second input is the pathways of interest with gene symbols.

**Important**: The default behavior of `fastwilcoxGMTall` is to calculate p-values for a two-sided test. However, when performing an enrichment analysis on the gene correlation results from the categorical omnibus test (either ANOVA or Kruskal Wallis), the p-values should be for a one-sided test. This is because, the omnibus tests are one-sided, where only more positive test statistics indicate strength of correlation. As shown below, the user should specify this by setting the `alternative` parameter to `"greater"`. For the pairwise tests, the default two-sided behavior is correct.

**Output** is the enrichment statistics from each pathway including the genes in the pathway and their ranks.

```
# read in the annotations
annots = read.gmt("gmtfile.gmt")
```

```
# format in a list
annotlist=list(annots)
names(annotlist)="MSigDBpathways"

# calculate enrichment statistics for the results including all categories
# specify alternative = "greater" to get p-values for a one-sided test
allenrichments = fastwilcoxGMTall(getStat(allresults), annotlist, outputGeneVals=T,
                                  alternative="greater")
```

```
## 25 results for annotation set MSigDBpathways
```

```
# View the stat, pval, and p.adj of the top enrichment results
head(allenrichments$MSigDBpathways[order(allenrichments$MSigDBpathways$pval),])[1:3]
```

```
##                                                          stat       pval
## REACTOME_TRANSPORT_OF_SMALL_MOLECULES              0.13212670 0.03847459
## REACTOME_METABOLISM_OF_LIPIDS                      0.12070707 0.06308092
## FLECHNER_BIOPSY_KIDNEY_TRANSPLANT_REJECTED_VS_OK_DN 0.12366310 0.08660459
## DODD_NASOPHARYNGEAL_CARCINOMA_DN                   0.12408759 0.09546697
## NUYTTEN_EZH2_TARGETS_DN                            0.11970803 0.10353506
## ZWANG_TRANSIENTLY_UP_BY_2ND_EGF_PULSE_ONLY         0.09989259 0.10983787
##                                                        p.adj
## REACTOME_TRANSPORT_OF_SMALL_MOLECULES              0.4576578
## REACTOME_METABOLISM_OF_LIPIDS                      0.4576578
## FLECHNER_BIOPSY_KIDNEY_TRANSPLANT_REJECTED_VS_OK_DN 0.4576578
## DODD_NASOPHARYNGEAL_CARCINOMA_DN                   0.4576578
## NUYTTEN_EZH2_TARGETS_DN                            0.4576578
## ZWANG_TRANSIENTLY_UP_BY_2ND_EGF_PULSE_ONLY         0.4576578
```

```
# the third table is for the pairwise comparison between low and medium species
# (with integer labels 2 and 3 respectively)
low_med_enrichments = fastwilcoxGMTall(getStat(pairwise_tables[[3]]), annotlist, outputGeneVals=T)
```

```
## 21 results for annotation set MSigDBpathways
```

```r
# View the stat, pval, and p.adj of the top enrichment results
head(low_med_enrichments$MSigDBpathways[order(low_med_enrichments$MSigDBpathways$pval),])[1:3]
```

```
##                                                 stat       pval     p.adj
## REACTOME_TRANSPORT_OF_SMALL_MOLECULES     0.18331053 0.01418013 0.2977827
## HOUNKPE_HOUSEKEEPING_GENES               -0.12867647 0.17515792 0.7883529
## KINSEY_TARGETS_OF_EWSR1_FLII_FUSION_UP -0.10150376 0.22785575 0.7883529
## CHEN_METABOLIC_SYNDROM_NETWORK           -0.07977621 0.26286102 0.7883529
## REACTOME_METABOLISM_OF_LIPIDS             0.08625954 0.27461457 0.7883529
## BLALOCK_ALZHEIMERS_DISEASE_UP            -0.08052885 0.29402292 0.7883529
```

For a more in depth explanation of performing an enrichment analysis, see the "RERconverge Analysis Walkthrough" vignette.

# Conclusion

This concludes the walk through of how to use the new functions in RERconverge for analyzing categorical traits. Thank you!

Louca, Stilianos, and Michael Doebeli. 2017. "Efficient Comparative Phylogenetics on Large Trees." https://doi.org/10.1093/bioinformatics/btx701.

Ogle, Derek H., Jason C. Doll, Powell Wheeler, and Alexis Dinno. 2022. "FSA: Fisheries Stock Analysis." https://github.com/fishR-Core-Team/FSA.

Paradis, E., and K. Schliep. 2019. "Ape 5.0: An Environment for Modern Phylogenetics and Evolutionary Analyses in {r}" 35: 526–28.

Revell, Liam J. 2012. "Phytools: An r Package for Phylogenetic Comparative Biology (and Other Things)." 3: 217–23.

# Ancestral State Reconstruction for Binary and Categorical Traits Walkthrough

November 26, 2022

## Contents

**Overview** **2**

**Getting Started** **2**
Data Input Requirements and Formatting . . . . . . . . 2
Reading in the Data . . . . . . . . 3

**Finding the Optimal Rate Model** **3**
Specifying a Rate Model . . . . . . . . 3
Performing a Rate Model Search . . . . . . . . 4
Comparing Rate Models . . . . . . . . 7
Assessing Prediction Accuracy . . . . . . . . 9

**Visualizing Reconstructions Under Different Rate Models** **17**

**Conclusion** **19**

**References** **19**

This walkthrough describes the phenotype inference step of an RERconverge analysis in more detail. The method of ancestral state reconstruction described in this walkthrough can be used with binary or categorical traits, but not continuous traits. This walkthrough builds on existing RERconverge objects. First time users should refer to the "RERconverge Analysis Walkthrough" vignette and the "Categorical Trait Analysis Walkthrough" vignette.

# Overview

In order to follow along with this walkthrough, you will first need to install RERconverge, read in your gene trees, and read in your phenotype data. For detailed instructions on how to install RERconverge, reference the RERconverge install page. For instructions on reading in trees and phenotype data, reference the "RERconverge Analysis Walkthrough" vignette.

Every RERconverge analysis involves a phenotype inference step, in which the phenotype data for the living species at the tips of the tree is used to infer the phenotypes of ancestral species in the tree. For categorical traits, RERconverge uses maximum likelihood estimation to obtain the likelihoods at each internal node of being in any given state of the phenotype. This method can just as easily be used on binary traits though it works differently than the method currently used by RERconverge for binary traits.

Maximum likelihood estimation requires a model of evolution to describe the probability of transitioning between phenotype states. This model is a continuous time markov model, in which a square transition matrix, denoted Q, describes the instantaneous rate of transitioning from state i to state j at position (i,j). All off diagonal elements of the transition matrix are non-negative, while the diagonal elements are negative such that each row sums to zero. On each branch of the tree, the transition probability matrix, P, is obtained by taking the exponential of Q multiplied by the branch length. One nice property of this formulation is that the rows of P will sum to 1 such that the entries represent probabilities of transitioning between states. The probabilities on the diagonal represent the probability of staying in a given state. The true rates in the transition matrix are hardly ever known for sure. Therefore, they are inferred in order to maximize the probability of the observed data at the tips of the tree. This walkthrough will refer to the process of estimating optimal transition rates as fitting the transition matrix.

RERconverge relies on functions from the castor(Louca and Doebeli 2017) and ape(Paradis and Schliep 2019) packages to fit the transition matrix and obtain ancestral likelihoods. Currently, RERconverge assigns to each node the state with the maximum likelihood.

Thus, to obtain ancestral states in the phenotype tree, an RERconverge user must only supply a phylogenetic tree, phenotype data, and a rate model. The rate model describes constraints on fitting the transition matrix. For instance, an equal rates model forces every transition rate to be the same. On the other hand, an all rates different model allows each transition rate to take on an independent value.

Rate models can be used to incorporate prior biological knowledge. For instance, position (i,j) can be set to zero if you know that transitions between state i and j cannot happen directly. It is important to note that the transition matrix describes instantaneous transition rates. Multiple transitions can occur along a single branch in the tree. Thus, setting position (i,j) to zero does not prevent an i to j transition from occurring along a single branch.

This walkthrough will review the diagnostics provided by RERconverge for choosing the optimal rate model for your analysis.

# Getting Started

## Data Input Requirements and Formatting

The required inputs are as follows:

1. Phylogenetic trees of the same format described in the "RERconverge Analysis Walkthrough" vignette.

2. Species-labeled phenotype values

- The species labels MUST match the tree tip labels that were used in `getAllResiduals` to calculate the relative evolutionary rates (RERs)
- a named vector of categorical trait values

### Reading in the Data

For the purpose of this walkthrough, we will read in the gene trees and phenotype data as shown below. The data used in this walkthrough is on mammal sleep patterns, a categorical trait with the following categories: cathemeral, crepuscular, diurnal and nocturnal.

For more details about reading in the specific data you need for your analysis, refer to the "Categorical Trait Analysis Walkthrough" vignette and the "RERconverge Analysis Walkthrough" vignette.

```
# check that RERconverge was successfully installed
library(RERconverge)

# find where the package is located on your machine
rerpath = find.package('RERconverge')

# read in the trees with the given file name
toytreefile = "subsetMammalGeneTrees.txt"
toyTrees=readTrees(paste(rerpath,"/extdata/",toytreefile,sep=""),
max.read = 200)

# load the phenotype data into your workspace
# This will create a named vector with the name basalRate
data("basalRate")
```

## Finding the Optimal Rate Model

The "optimal" rate model depends on a number of factors, and even then there may be no way of knowing the true best rate model. Therefore, the diagnostics that will be described in this section are designed to provide more information and insight about potential rate models in order to help you make an educated selection. These diagnostics do not take prior biological knowledge into account. Thus it is up to the user to interpret the results, possibly rejecting models that do not make sense biologically.

Most of the diagnostics explained below use the likelihood ratio to compare rate models. The likelihood ratio is calculated as $-2 \times log_2(\frac{\text{likelihood of simpler model}}{\text{likelihood of more complex model}})$. When the simpler model is a special case of the more complex model (a.k.a. when the models are nested), then the likelihood ratio is distributed as a chi squared distribution with the degrees of freedom equal to the difference in the number of free parameters between the simpler and more complex models. When they are not nested, the p-value can still be obtained by using simulations to generate the null distribution for the likelihood ratio (Pagel 1994).

### Specifying a Rate Model

All rate models can be described by a square matrix with dimensions equal to the number of phenotype states. The diagonal elements must be zero because they are dependent on the value of the off-diagonal elements, thus are not a free parameter. Everywhere else in the matrix, each free parameter should be assigned a unique number and any off-diagonal elements that are set to zero represent transitions that will be set to zero in the transition matrix. The order of unique numbers does not matter as long as the numbers are consecutive and begin at 1. For example, the all rates differnt model can be specified as `"ARD"` for short or, equivalently for a phenotype with three states, `matrix(c(0,1,2,3,0,4,5,6,0),3)`. The equal rates model can be specified as `"ER"` for short, or equivalently for a phenotype with three states,

`matrix(c(0,1,1,1,0,1,1,1,0),3)`. Finally, the rate model abbreviated as `"SYM"` for a symmetric model is equivalent to `matrix(c(0,1,2,1,0,3,2,3,0),3)`in which every rate (i,j) is equal to the rate (j,i).

## Performing a Rate Model Search

One guiding principle for selecting a rate model is to choose the simplest model that still fits the data well. However, the number of all possible rate models to search over becomes too large once the number of phenotype categories exceeds two or three. Therefore, the `searchRateModels` function uses an iterative approach to find simpler models that don't sacrifice their ability to fit the data. This approach was inspired by an algorithm described in Jayaswal 2011 (Jayaswal et al. 2011). A brief overview of the method is as follows:

1. A list is created to store the rate models. To begin with, the list will only contain the all rates different (ARD) model which allows every transition to take on a different value and is thus the most complex model. We start our counter at `i = 1` and fit a transition matrix using the `ith` rate model in this list, which to begin with is the ARD model. However, if the `ith` rate model has only one free parameter (the simplest rate model), we do not move on to step 2 since we cannot generate simpler models from this model. Instead we skip to step 4 to move on to the next rate model in the list. If there is no next model, we are done.
2. Based on the transition matrix, we generate two new models that decrease the number of free parameters. In the first new model, we set the two positions with the closest transition rates equal. In the second new model, we set the position with the smallest transition rate to zero. More precisely, this step may generate more than two new models if there are multiple positions in the transition matrix with the same value, but each new model is generated similarly to one of the two ways described above.
3. Fit the new models to the data and calculate the likelihood ratios between the previous model and each new model. If the previous, more complex, model is NOT significantly better at describing the observed data as determined by the likelihood ratio, then we add the new model to the list of rate models. The new model represents a simpler model that is equally capable of describing the observed data.
4. Increment the counter, `i = i + 1`.

Eventually, no new models are added to the list of rate models either because they cannot be simplified further or because any simpler model would not be able to describe the observed data as well as the more complex models.

This function **outputs** the list of rate models that was generated during the search. It also outputs a table containing the likelihood ratios between the new models and previous models that were computed on each iteration of the algorithm. When the models are nested, a p-value for the likelihood ratio is also included in the table. In this table, `prevIndex` and `newIndex` refer to the indices of the previous and new rate models in the list of rate models that is also returned by this function. This table also contains the AIC and loglikelihood of fitting the transition matrix in step 1 above.

To perform a rate model search use `searchRateModels`, which takes the following inputs:

- `treesObj`: the trees object returned by `readTrees` (see the "RERconverge Analysis Walkthrough" vignette for information about `readTrees`)
- `phenvals`: the named phenotype vector
- `pthreshold`: If the likelihood ratio has a p-value that is less than or equal to `pthreshold`, then the more complex model is considered significantly better at fitting the transition matrix to the observed data and the new model is discarded. `pthreshold` is only used when the new model and previous model are nested, thus a p-value can be obtained from the chi squared distribution.
- `lthreshold`: The threshold for the likelihood ratio used when the previous model and new model are not nested, thus a p-value cannot be obtained from the chi squared distribution. The simulation based approach is not used in this case to determine a p-value because it is too time consuming. If the

likelihood ratio is above this threshold, then the more complex model is considered significantly better at fitting the transition matrix to the observed data and the new model is discarded.

- `max_iterations`: If not `NULL`, the algorithm will stop when `i > max_iterations` in order to avoid long run times. The default is `2000`. When an analysis is stopped due to exceeding the `max_iterations`, a message indicating such will be printed to the console.
- `...`: additional parameters for `fit_mk`, the castor function used to fit the transition matrix from the rate model

Increasing `pthreshold` and decreasing `lthreshold` weakens the conditions under which the more complex model is considered significantly better. Equivalently, it creates stricter conditions for considering a simpler rate model equally capable of fitting the transition matrix to the observed data. This *increases* the number of simpler rate models that get discarded, speeding up the analysis and limiting the total number of results that are generated.

```
library(RERconverge)
search_res = searchRateModels(toyTrees, basalRate, pthreshold = 0.25, lthreshold = 1.3)

# view the generated models
tail(search_res$models)

# view the model statistics
tail(search_res$stats)
```

Although `searchRateModels` significantly reduces the search space, it can still generate over one thousand models. In order to filter through these models, use `filterByCriteria`, which takes the following parameters:

- `models`: The list of rate models to filter
- `criteria`: A function specifying the filter criteria. The body of this function must be a boolean expression, the form of which is shown below. This function may be used to filter for models that match prior biological knowledge and/or to filter for models with a certain number of zero transitions and/or number of free parameters.

Use `map_to_state_space` to see which indices in the rate models correspond to the categories in your analysis.

```
# View the category to integer mapping
map_to_state_space(basalRate)$name2index
```

```
## high  low  med
##    1    2    3
```

```
# rm is a rate model in the list returned by searchRateModels
# Example 1: If you know something about transitions between states
criteria1 <- function(rm) {
  # filter out rate models in which the high to med transitions are not zero
  # and the high to low transitions are not zero
  rm[1,3] != 0 && rm[1,2] != 0
}

filtered_res1 = filterByCriteria(search_res$models, criteria1)
tail(filtered_res1)
```

```
## [[1]]
##      [,1] [,2] [,3]
## [1,]    0    1    2
## [2,]    0    0    1
## [3,]    1    2    0
```

```
##
## [[2]]
##      [,1] [,2] [,3]
## [1,]    0    1    2
## [2,]    2    0    1
## [3,]    1    0    0
##
## [[3]]
##      [,1] [,2] [,3]
## [1,]    0    1    2
## [2,]    2    0    1
## [3,]    1    1    0
##
## [[4]]
##      [,1] [,2] [,3]
## [1,]    0    1    1
## [2,]    1    0    0
## [3,]    1    1    0
##
## [[5]]
##      [,1] [,2] [,3]
## [1,]    0    1    2
## [2,]    1    0    0
## [3,]    1    1    0
##
## [[6]]
##      [,1] [,2] [,3]
## [1,]    0    1    1
## [2,]    1    0    2
## [3,]    1    1    0
```

```
# Example 2: filtering by number of zeros and number of free parameters
criteria2 <- function(rm) {
  # no more than 3 transitions between different states set to zero
  # (recall there will be 3 zeroes from the diagonal, so <= 6 zeroes overall)
  # and no more than 4 free parameters
  sum(rm == 0) <= 6 && max(rm) <= 4
}

filtered_res2 = filterByCriteria(search_res$models, criteria2)
tail(filtered_res2)
```

```
## [[1]]
##      [,1] [,2] [,3]
## [1,]    0    1    1
## [2,]    1    0    0
## [3,]    1    1    0
##
## [[2]]
##      [,1] [,2] [,3]
## [1,]    0    1    0
## [2,]    1    0    2
## [3,]    1    1    0
##
## [[3]]
```

```
##      [,1] [,2] [,3]
## [1,]    0    1    2
## [2,]    1    0    0
## [3,]    1    1    0
##
## [[4]]
##      [,1] [,2] [,3]
## [1,]    0    1    1
## [2,]    1    0    2
## [3,]    1    1    0
##
## [[5]]
##      [,1] [,2] [,3]
## [1,]    0    1    0
## [2,]    0    0    1
## [3,]    1    2    0
##
## [[6]]
##      [,1] [,2] [,3]
## [1,]    0    1    0
## [2,]    2    0    1
## [3,]    1    1    0
```

## Comparing Rate Models

Once you have a set of candidate rate models, you can use `compareRateModels` to perform pairwise likelihood ratio comparisons and determine which rate model best fits the observed data. For instance, one could select a subset of rate models from the rate model search described previously. The rate model comparison is fastest and most interpretable on a small set of ten or fewer rate models.

The **output** of `compareRateModels` is a square table of either p-values or likelihood ratios for pairwise comparisons between rate models. The diagonal of the table contains only `NA` values since rate models are not compared to themselves. Additionally, each comparison is only performed once with the simpler model in the numerator. Thus if position (i,j) in the table has a p-value or likelihood ratio, you should expect position (j,i) to be `NA`.

The model corresponding to the row will always be the simpler model and the model corresponding to the column will always be the more complex model. Thus, a small p-value or large likelihood ratio is evidence that the more complex model, corresponding to the column, provides a significantly better fit to the data at the tips. On the other hand, a large p-value or small likelihood ratio is evidence that the simpler model, corresponding to the row, is equally capable of providing a good fit to the data as the more complex model.

The code below runs you through how to make a list of rate models and run it on `compareRateModels` which takes the following as input:

- `rate_models`: a named list of rate models to compare. All rate models in the list must be matrices. Use the function `getMatrixFromAbbr` to convert `"ER"`, `"ARD"`, and `"SYM"` to their respective matrices.
- `treesObj`: the trees object returned by `readTrees`.
- `phenvals`: the named phenotype vector
- `nsims`: the number of simulations to use in order generate the null distribution of likelihood ratios for calculating a p-value when two models are not nested. The default value is `100`. Decreasing `nsims` would decrease run time at the expense of statistical validity.
- `nested_only`: a boolean specifying whether to only calculate p-values for nested models. The default is `FALSE`. Setting `nested_only` to `TRUE` speeds up the analysis, but will leave out comparisons between

non-nested models.

- `return_type`: The default value is `"pvals"`, meaning the comparisons between rate models are returned as p-values for the likelihood ratios. Alternatively, setting `return_type` to `"ratios"` will return the likelihood ratios themselves without calculating p-values. This is an alternative way to speed up the analysis while still calculating comparisons between non-nested models. Note: while p-values have traditional thresholds for statistical significance, interpreting likelihood ratios is less well defined. Nonetheless, the larger the likelihood ratio, the more support there is for the more complex model being a significantly better fit.
- `...`: additional parameters for `fit_mk`, the castor function used to fit the transition matrix from the rate model.

```
# the list of rate models includes the three common rate models-- "ER", "ARD", and "SYM"--, two rate mo

custom = matrix(c(0, 0, 1, 0, 0, 2, 0, 3, 0),3)

# getMatrixFromAbbr takes the abbreviation ("ER", "ARD", or "SYM") and the number of phenotype states
rate_models = list(getMatrixFromAbbr("ER", 3), getMatrixFromAbbr("ARD",3),
                   getMatrixFromAbbr("SYM",3), filtered_res1[[23]], filtered_res1[[5]],
                   custom)

names(rate_models) = c("ER", "ARD", "SYM", "search1", "search2", "custom")

comp = compareRateModels(rate_models = rate_models,
                         treesObj = toyTrees,
                         phenvals = basalRate,
                         nsims = 100,
                         nested_only = FALSE,
                         return_type = "pvals")
```

```
# view the comparisons
comp
```

```
##         ER     ARD     SYM search1 search2 custom
## ER      NA 0.70816 0.49896    0.23 0.41327   0.66
## ARD     NA      NA      NA      NA      NA     NA
## SYM     NA 0.66928      NA      NA 0.40000     NA
## search1 NA 0.95055 0.77000      NA 0.52000   0.74
## search2 NA 0.95875      NA      NA      NA     NA
## custom  NA 0.02121 0.00000      NA 0.00000     NA
```

```
# refer back to the rate models
rate_models
```

```
## $ER
##      [,1] [,2] [,3]
## [1,]    0    1    1
## [2,]    1    0    1
## [3,]    1    1    0
##
## $ARD
##      [,1] [,2] [,3]
## [1,]    0    3    5
## [2,]    1    0    6
## [3,]    2    4    0
##
```

```
## $SYM
##      [,1] [,2] [,3]
## [1,]    0    1    2
## [2,]    1    0    3
## [3,]    2    3    0
##
## $search1
##      [,1] [,2] [,3]
## [1,]    0    1    2
## [2,]    1    0    2
## [3,]    1    0    0
##
## $search2
##      [,1] [,2] [,3]
## [1,]    0    1    3
## [2,]    4    0    4
## [3,]    1    2    0
##
## $custom
##      [,1] [,2] [,3]
## [1,]    0    0    0
## [2,]    0    0    3
## [3,]    1    2    0
```

From the results above, we may conclude that the more complex models "ARD", "SYM", search1, and search2 were not significantly better at fitting the data compared to the "ER" model because the p-values in the "ER" row were relatively high. On the other hand, notice that the custom rate model had many transition rates set to 0 which does not seem very plausible in the context of this trait. This is reflected in the bottom row of the table in which the p-values are 0.021 and 0, indicating that the more complex models "ARD", "SYM", and search2 were significantly better at fitting the data compared to this implausible model. Additionally, the custom model was not significantly better at fitting the data compared to the simpler rate models "ER" and search1 (with p-values of 0.81 and 0.73 respectively). This is not to say that the model(s) with the best fit are necessarily the best model(s), however comparing goodness of fit is a useful diagnostic for narrowing down the search space of potential rate models and comparing a few candidate rate models to each other.

## Assessing Prediction Accuracy

The third method for comparing rate models aims to measure prediction accuracy rather than comparing goodness of fit to the observed data.

The RERconverge function, `boxPlotTest`, takes a list of rate models and returns a box plot for each rate model in the list. This function works as follows:

1. Get the next rate model in the list
2. Fit a transition matrix using the rate model obtained in step 1
3. Simulate `nsims` times from the transition matrix obtained in step 2 (using the `simulate_mk_model` function provided by the castor package)
4. For each simulation, use the simulated states at the tips of the tree to infer the states of internal nodes under every rate model in the list. Compare the inferred states to the actual states generated by the simulation and calculate the percentage of states that were assigned correctly.
5. Make a box plot showing the percent of correct predictions on the y axis and the rate model on the x axis.
6. Repeat steps 1-5 for every rate model in the list of rate models.

`boxPlotTest` takes the following parameters:

- `treesObj`: the trees object returned by `readTrees`
- `phenvals`: named phenotype vector
- `rate_models`: the list of rate models to test
- `nsims`: The number of simulations to use per transition matrix
- `confidence_threshold`: the default value is `NULL`. However, if a confidence threshold in the range [0,1] is provided, the percent of correct matches will only be calculated for nodes whose maximum likelihood is greater than or equal to the confidence threshold. This can be beneficial to see whether the more confident state assignments tend to be accurate and if accuracy breaks down at nodes with greater uncertainty, or not.
- `...`: additional parameters for `fit_mk`, the castor function used to fit the transition matrix from the rate model

Note that the current implementation of this function may take a few minutes to run on larger trees and will be fastest when all the rate models are symmetrical as long as `use_simmap` is `FALSE`.

```
boxPlots = boxPlotTest(toyTrees, basalRate, rate_models, nsims = 25)
```

When viewing the box plots you may see the following warning: `## Warning: Removed n rows containing non-finite values (stat_boxplot).` This is not a problem, it just means that for some of the simulations, the percent correct (on the y-axis) could not be calculated for one or more of the rate models. This occurs when the rate model is a poor fit to the simulated data thus producing `NaN` values when calculating ancestral likelihoods. (This occurred in the case of our custom model which was purposely chosen to represent an implausible model).

```
# View the box plots
boxPlots$plots[[1]]
```

```
## Warning: Removed 25 rows containing non-finite values (`stat_boxplot()`).
```

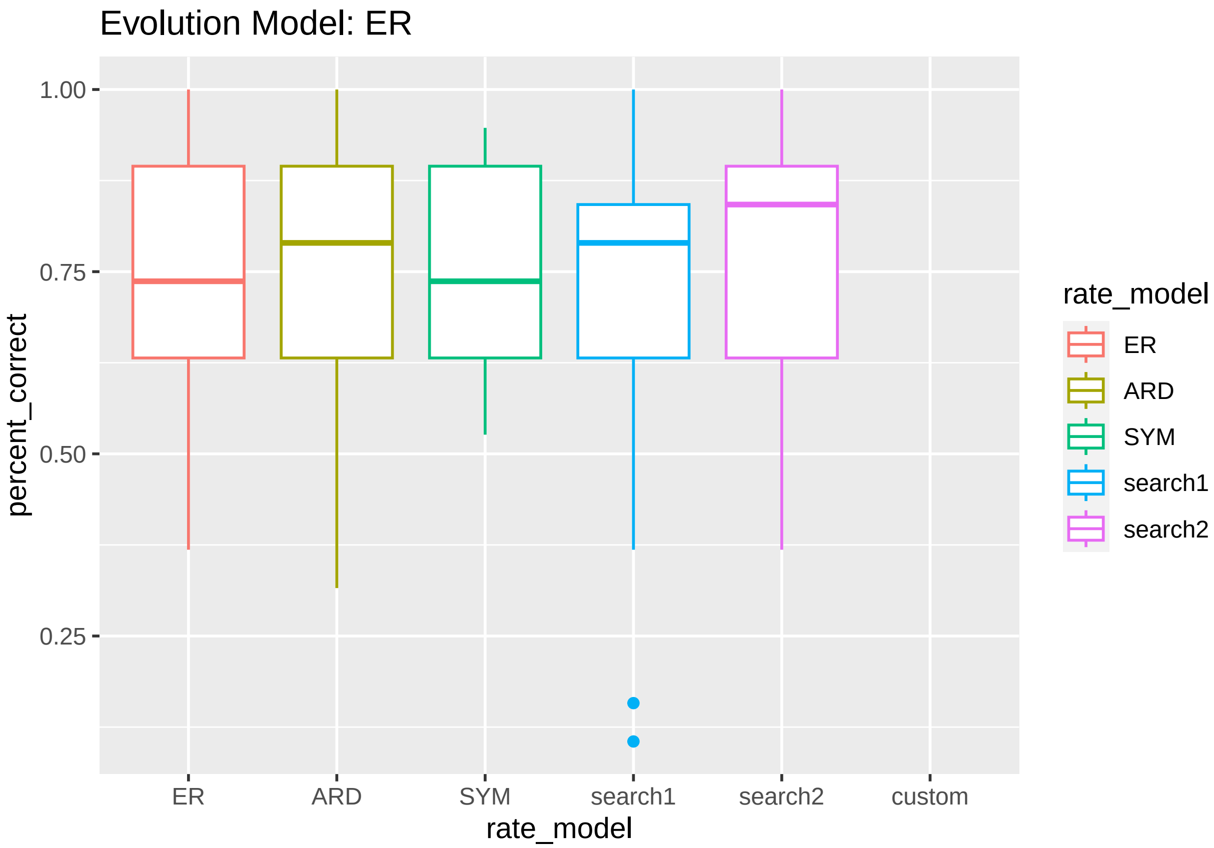


```
boxPlots$plots[[2]]
```

```
## Warning: Removed 29 rows containing non-finite values (`stat_boxplot()`).
```

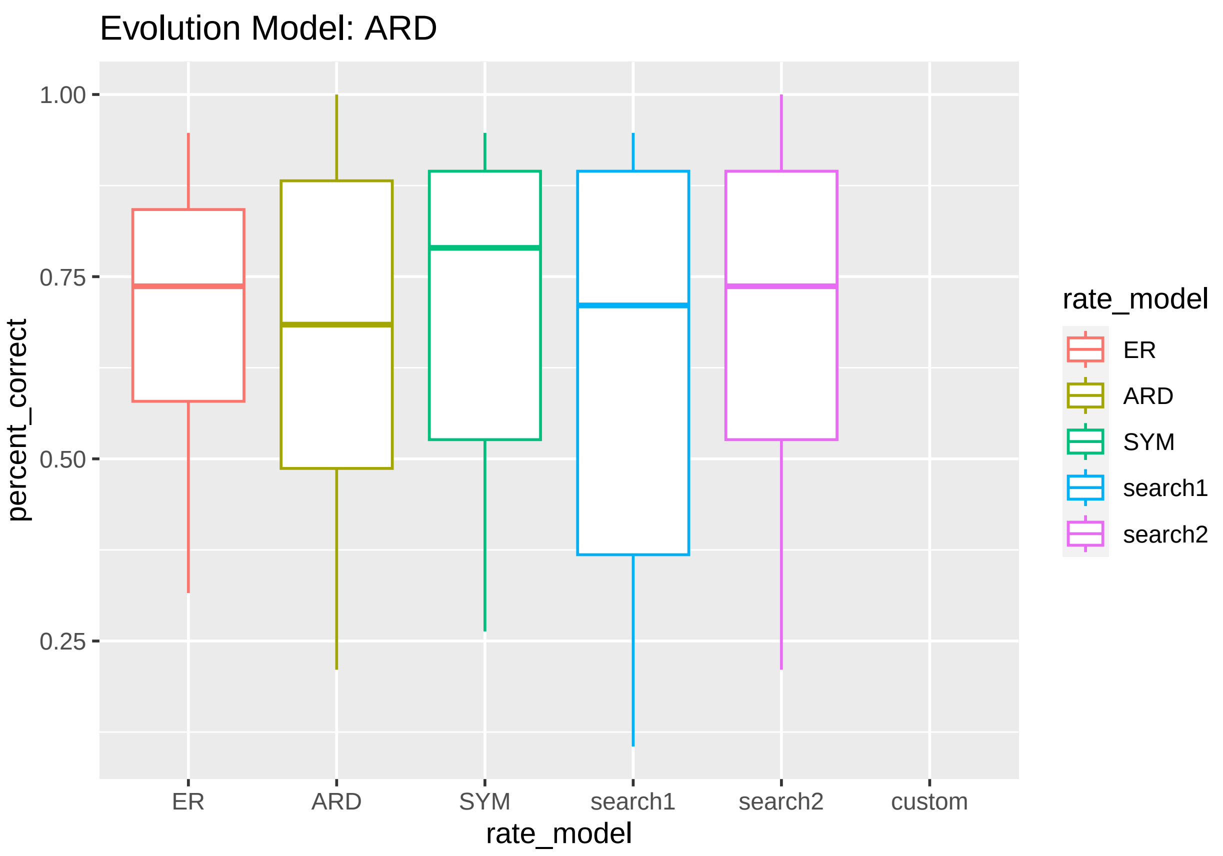


```
boxPlots$plots[[3]]
```

```
## Warning: Removed 29 rows containing non-finite values (`stat_boxplot()`).
```

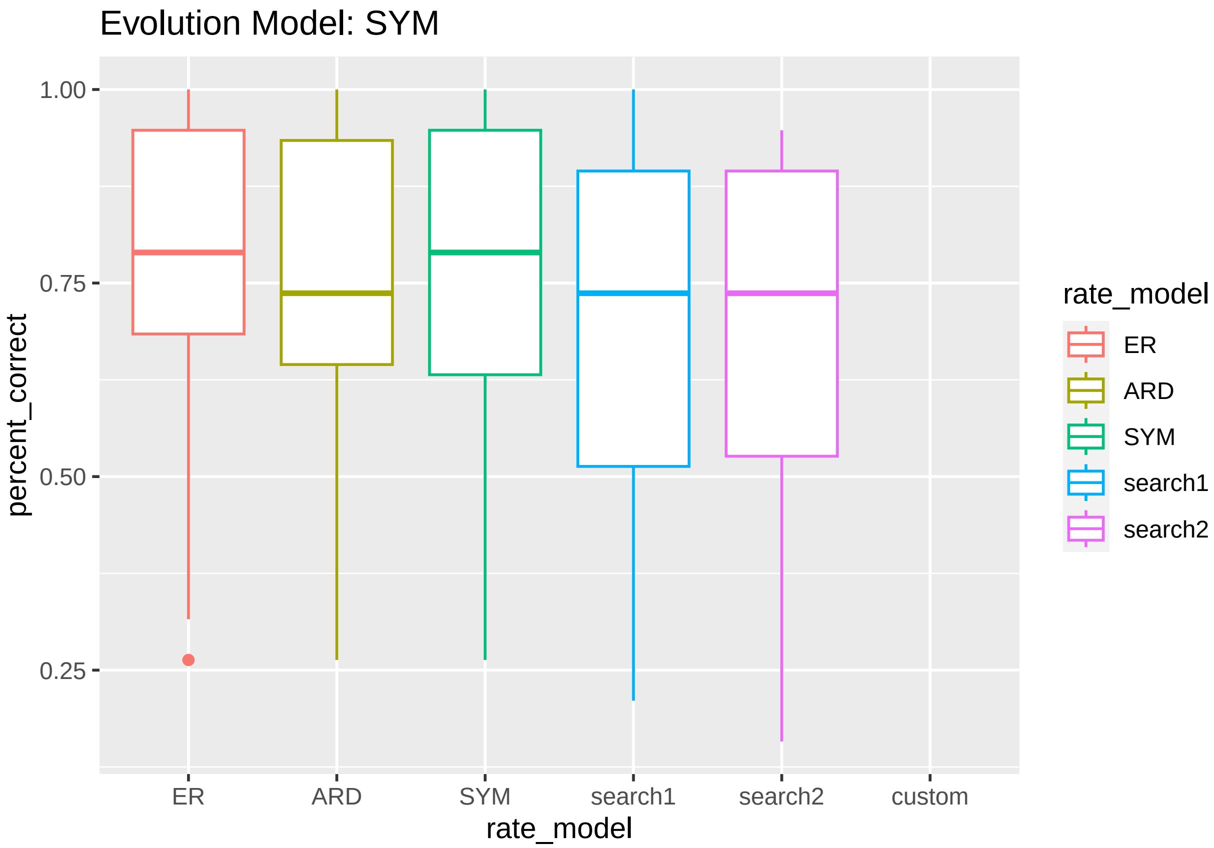


```
boxPlots$plots[[4]]
```

```
## Warning: Removed 27 rows containing non-finite values (`stat_boxplot()`).
```

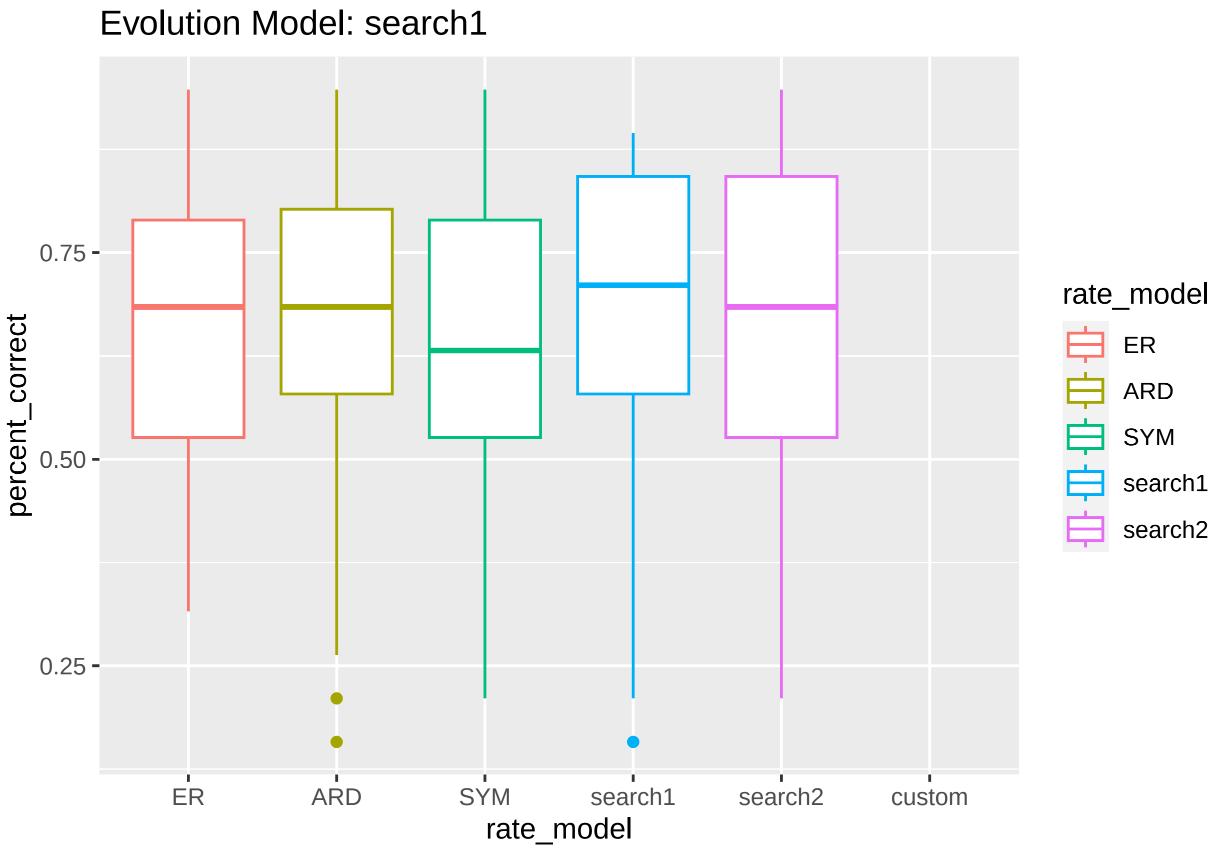


```
boxPlots$plots[[5]]
```

```
## Warning: Removed 28 rows containing non-finite values (`stat_boxplot()`).
```

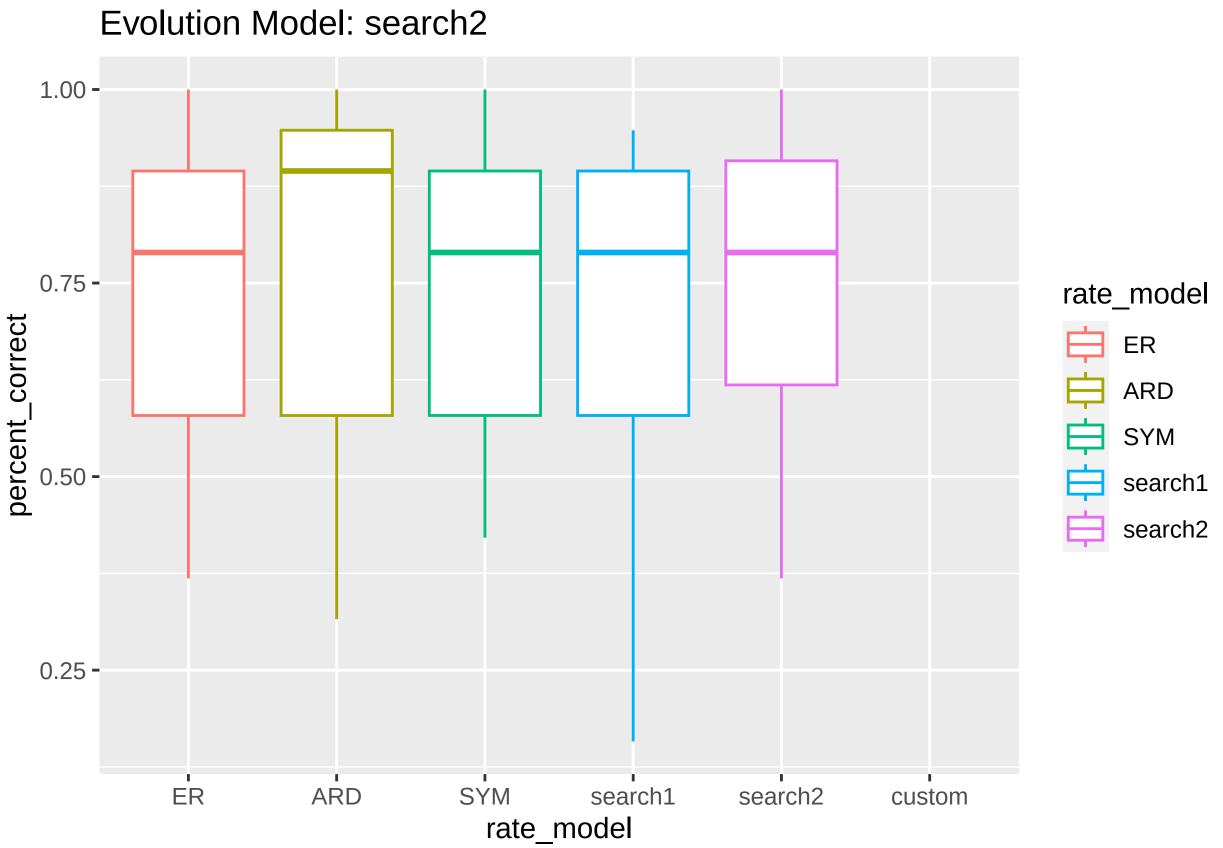


```
boxPlots$plots[[6]]
```

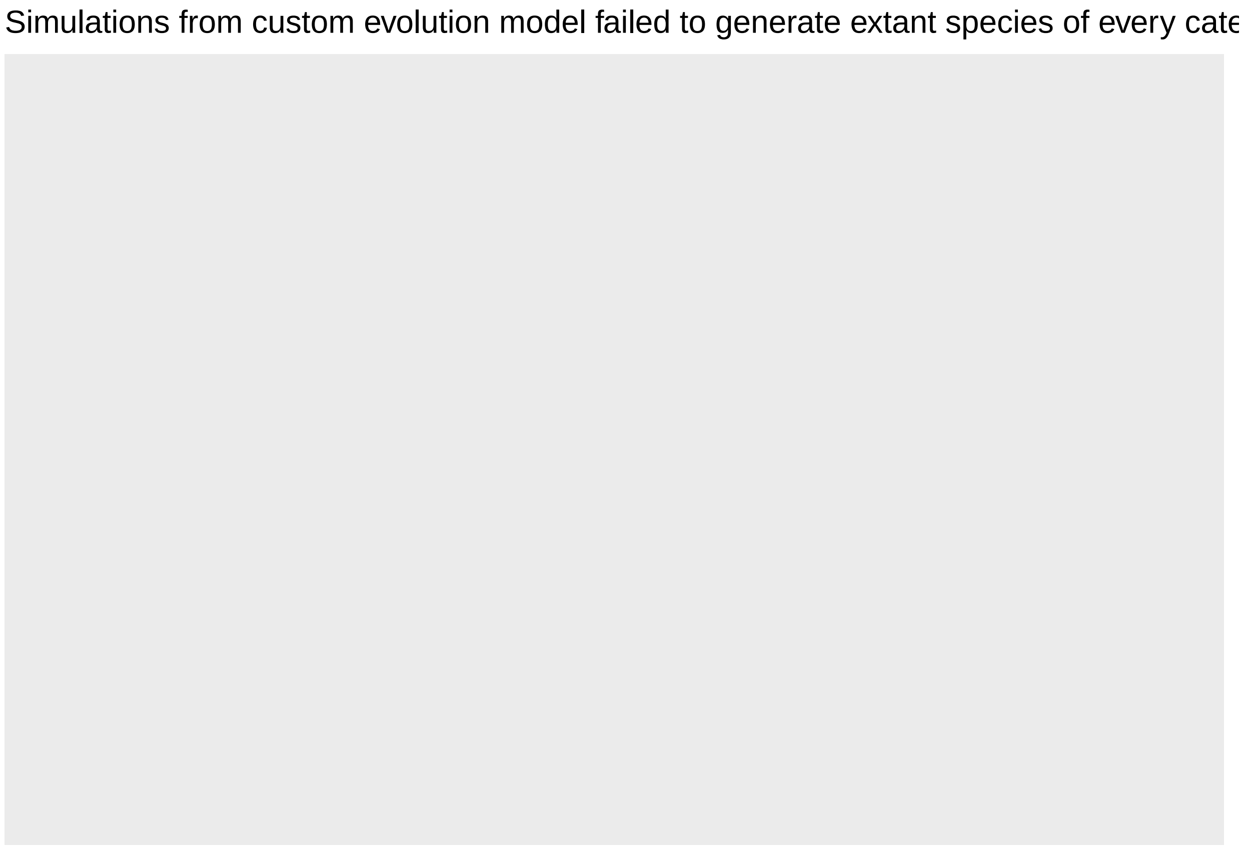


```r
# View the transition matrices fit under each model
boxPlots$transition_matrices
```

```
## $ER
##           [,1]      [,2]      [,3]
## [1,] -7.190801  3.595401  3.595401
## [2,]  3.595401 -7.190801  3.595401
## [3,]  3.595401  3.595401 -7.190801
##
## $ARD
##           [,1]      [,2]      [,3]
## [1,] -6.314164  6.314164  0.000000
## [2,]  2.302702 -6.164785  3.862083
## [3,]  6.034574  0.000000 -6.034574
##
## $SYM
##           [,1]       [,2]       [,3]
## [1,] -9.457521  7.1365768  2.3209441
## [2,]  7.136577 -8.0353985  0.8988216
## [3,]  2.320944  0.8988216 -3.2197657
##
## $search1
##           [,1]      [,2]      [,3]
## [1,] -8.277957  6.621821  1.656137
## [2,]  6.621821 -8.277957  1.656137
## [3,]  6.621821  0.000000 -6.621821
##
```

```
## $search2
##           [,1]      [,2]      [,3]
## [1,] -6.642621  6.642621  0.000000
## [2,]  3.478894 -6.957788  3.478894
## [3,]  6.642621  0.000000 -6.642621
##
## $custom
##          [,1]      [,2]      [,3]
## [1,]  0.00000   0.00000   0.00000
## [2,]  0.00000 -21.12679  21.12679
## [3,] 17.12491  49.35531 -66.48022
```

Simulating from a transition matrix that was fit on each rate model represents alternative models of evolution that the phenotype may have followed along the tree. Then under each model of evolution, the box plot allows you to evaluate the predication accuracy of each rate model. This can reveal whether one or more rate models perform better over others regardless of the model of evolution.

# Visualizing Reconstructions Under Different Rate Models

Finally, you can use `visCompareTwoRateModels` to compare how closely ancestral likelihoods or ancestral state assignments agree between two different rate models. `visCompareTwoRateModels` takes the following as input:

- `A`: the first rate model
- `B`: the second rate model
- `treesObj`: the trees object returned by `readTrees`
- `phenvals`: the named phenotype vector
- `mode`: the default mode is `"entropy"`. Under this mode, the relative entropy between the ancestral likelihoods at each node is calculated and plotted along the branches of the tree. Red corresponds to greater relative entropy, indicating greater disagreement between ancestral likelihoods. Blue corresponds to lower relative entropy, indicating greater agreement between ancestral likelihoods. The other option is `"match"`, which measures whether or not the states with the maximum ancestral likelihood at each node are the same between the different rate models. A red node indicates that the states differ and a blue node indicate that they are the same.
- `cex`: A graphing parameter for setting the font size of node labels and tree tip labels. The default value is `0.5`.
- `...`: additional parameters for `asr_mk_model`, the castor function for calculating ancestral likelihoods.

The function returns either a list of relative entropies or a table of state assignments under each rate model in node order. It also returns the lists of ancestral likelihoods for each rate model. The numbers on the nodes are simply the node indices in the tree.

```r
# comparing rate_models$search2 to rate_models$ER
relative_entropies = visCompareTwoRateModels(rate_models$ER,
                                             rate_models$search2,
                                             toyTrees,
                                             basalRate, mode = "entropy")
```

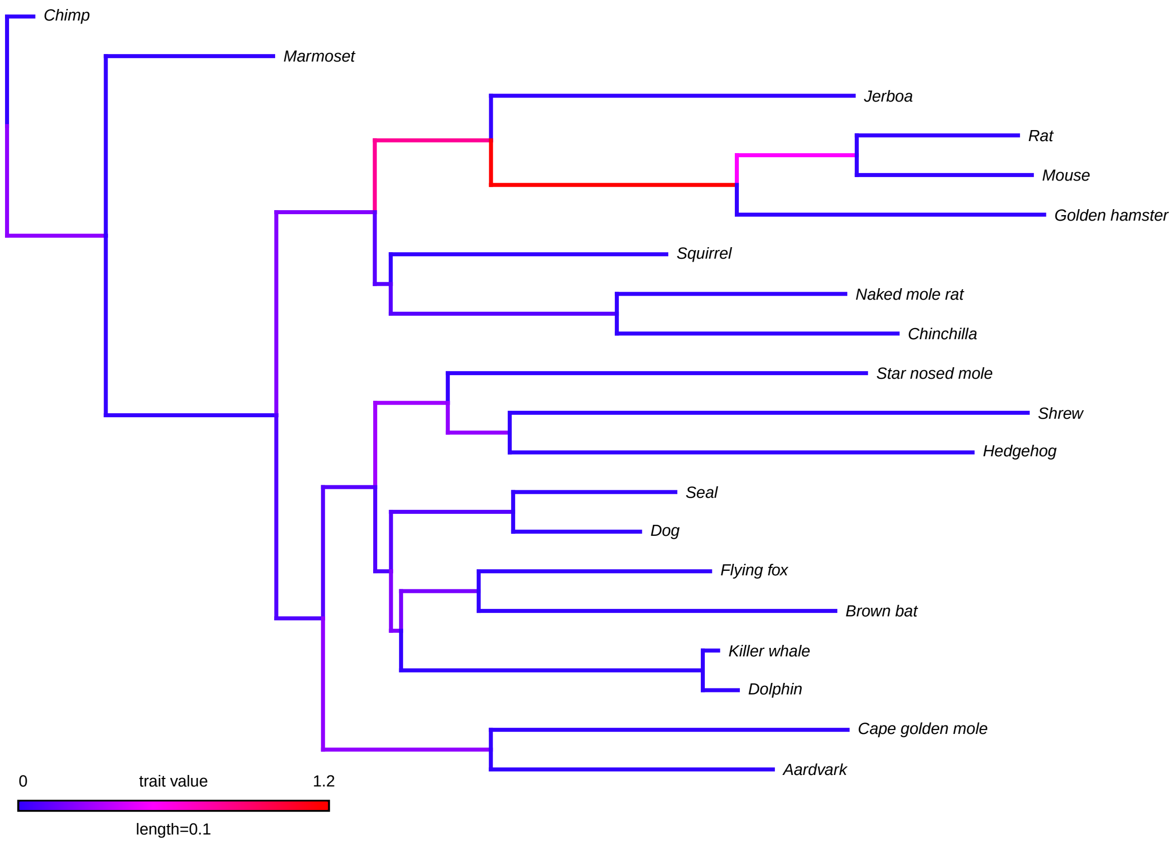


```
head(relative_entropies$relative_entropies)
```

```
##      21      22      23      24      25      26
## 0.01542 0.24476 0.00612 0.08126 0.25210 0.09531
```

```
head(relative_entropies$anc_liks1) # ancestral likelihoods for the ER model
```

```
##           [,1]       [,2]       [,3]
## [1,] 0.9112745 0.06928253 0.01944302
## [2,] 0.6262402 0.32951222 0.04424757
## [3,] 0.6440816 0.30563231 0.05028610
## [4,] 0.7153664 0.23712253 0.04751107
## [5,] 0.3672484 0.40331141 0.22944017
## [6,] 0.8769956 0.10495617 0.01804819
```

```
head(relative_entropies$anc_liks2) # ancestral likelihoods for the search2 model
```

```
##           [,1]       [,2]       [,3]
## [1,] 0.9466677 0.03994015 0.01339210
## [2,] 0.3544074 0.61749880 0.02809378
## [3,] 0.6136817 0.31539486 0.07092345
## [4,] 0.7366194 0.14180587 0.12157471
## [5,] 0.1351489 0.60372694 0.26112417
## [6,] 0.9117855 0.03337857 0.05483590
```

```
node_states = visCompareTwoRateModels(rate_models$ER,
                                      rate_models$search2,
                                      toyTrees, basalRate,
                                      mode = "match")
```

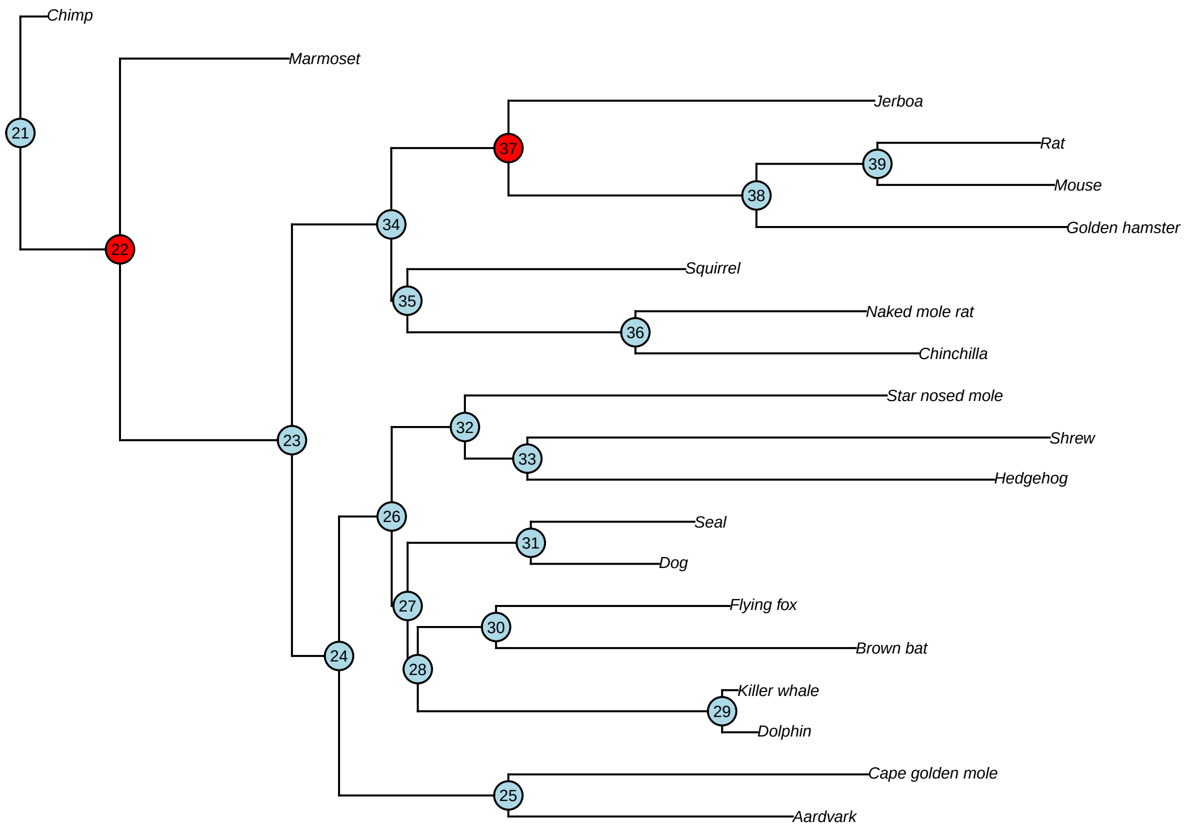


```
head(node_states$states)
```

```
##    statesA statesB
## 21       1       1
## 22       1       2
## 23       1       1
## 24       1       1
## 25       2       2
## 26       1       1
```

# Conclusion

This walkthrough reviewed some diagnostics provided by RERconverge to make an informed decision on which rate model to use in the phylogenetic inference step of a binary or categorical RERconverge analysis. After using one or more of the tools above to select a rate model, run `char2TreeCategorical` and/or `char2PathsCategorical` with that rate model and proceed with the RERconverge analysis as described in the "RERconverge Analysis Walkthrough" vignette or the "Categorical Trait Analysis Walkthrough" vignette.

# References

Pagel, Mark. "Detecting Correlated Evolution on Phylogenies: A General Method for the Comparative Analysis of Discrete Characters." *Proceedings: Biological Sciences*, vol. 255, no. 1342, 1994, pp. 37–45. *JSTOR*, http://www.jstor.org/stable/49836. Accessed 26 Nov. 2022.

Jayaswal, V., F. Ababneh, L. S. Jermiin, and J. Robinson. 2011. "Reducing Model Complexity of the General Markov Model of Evolution." *Molecular Biology and Evolution* 28 (11): 3045–59. https://doi.org/10.1093/molbev/msr128.

Louca, Stilianos, and Michael Doebeli. 2017. "Efficient Comparative Phylogenetics on Large Trees." https://doi.org/10.1093/bioinformatics/btx701.

Paradis, E., and K. Schliep. 2019. "Ape 5.0: An Environment for Modern Phylogenetics and Evolutionary Analyses in {r}" 35: 526–28.

# Categorical Permulation Walkthrough

Ruby Redlich

20 December, 2023

# Introduction

This walkthrough explains how to perform permulation analysis to calculate empirical p-values for genes and pathways for Categorical traits. For a description of what permulations are and why they are important, refer to the Permulations Walkthrough.

## Categorical Permulations Overview

Categorical permulations are accomplished slightly differently than binary and categorical permulations because the simulation step is not based on a Brownian motion model. Instead, a simulated phenotype is generated according to a continuous time Markov Model, the same model that was used to reconstruct the ancestral history of the trait. As with binary and categorical permulations, the simulation is based on a phylogeny with branch lengths representing the average genome-wide evolutionary rate along that branch. Next, 3 steps are taken to ensure that the permulated phenotype contains the same number of species with each trait value as the original phenotype:

1) Rejection: any simulated phenotype in which there are not the same number of **extant** species with each trait value as the original phenotype is rejected.
2) Permutation of internal traits: the simulated values for **internal** species are ignored. Instead, the original trait values for the internal species in the phylogeny are permuted and assigned to internal species in the permulated phenotype.
3) Re-organize internal traits: a search technique similar to simulated annealing is used to re-organize the internal traits relative to the traits of the extant species to improve the likelihood of the permulated phenotype. This generates a plausible trait history that exactly matches trait category counts and has a comparable probabilistic likelihood to the original simulation.

**Note that the permulation functions can take a long time to run on large data sets and for large numbers of permulations.**

# Categorical Permulations

This vignette will use the basal metabolic rate (BMR) categorical phenotype to demonstrate how to run a categorical permulation analysis. This vignette will briefly walk through the steps for ancestral state reconstruction (ASR) and calculating correlation statistics that are required for this analysis, but for more details regarding these steps please refer to the Categorical Walkthrough and the ASR Walkthrough.

## Getting Started With a Categorical Trait Analysis in RERconverge

Start by loading the RERconverge library. For more detailed instructions on getting started with RERconverge, refer to the RERconverge Analysis Walkthrough vignette.

```
if (!require("RERconverge", character.only = T, quietly = T)) {
  require(devtools)
  install_github("nclark-lab/RERconverge", ref = "master")
  # ref refers to the branch of RERconverge being installed
}
library(RERconverge)
```

Next read in the phenotype data and the gene trees. Additionally, calculate the relative evolutionary rates. For more details on using `readTrees` and `getAllResiduals` refer to the RERconverge Analysis Walkthrough vignette.

```
# find where the package is located on your machine
rerpath = find.package('RERconverge')

# read in the trees with the given file name
toytreefile = "subsetMammalGeneTrees.txt"
toyTrees=readTrees(paste(rerpath,"/extdata/",toytreefile,sep=""), max.read = 200)

# load the phenotype data into your workspace
# This will create a named vector with the name basalRate
data("basalRate")

# calculate the relative evolutionary rates with getAllResiduals
RERmat = getAllResiduals(toyTrees, useSpecies = names(basalRate))
```

The next steps is to infer the phenotypes of the ancestral species and calculate paths using the function `char2PathsCategorical`. For the purpose of this walkthrough, we will just use `"ARD"` (all rates different) as the model of evolution. (Note that the choice of rate model impacts the ancestral reconstruction and the analysis. Refer to the ASR Walkthrough for more details).

The `toyTrees` object contains a separate gene tree for each gene in the analysis with branch lengths representing the evolutionary rates of that gene. All of the gene trees have the same overall topology as the master tree, but some of them are missing certain species. To handle missing species, RERconverge generates something called paths. For a more detailed discussion of paths see the "RERconverge Analysis Walkthrough" vignette.

```
# get the names of all the species for which there is phenotype data
allspecs = names(basalRate)

# infer ancestral states and calculate paths
charP = char2PathsCategorical(basalRate, toyTrees, useSpecies = allspecs, model = "ARD",
                              plot = TRUE)
```

```
## Species from master tree not present in useSpecies: Platypus,Opossum,Tasmanian_devil,Wallaby,Armadil

## [1] "The integer labels corresponding to each category are:"
## high  low  med
##    1    2    3
```

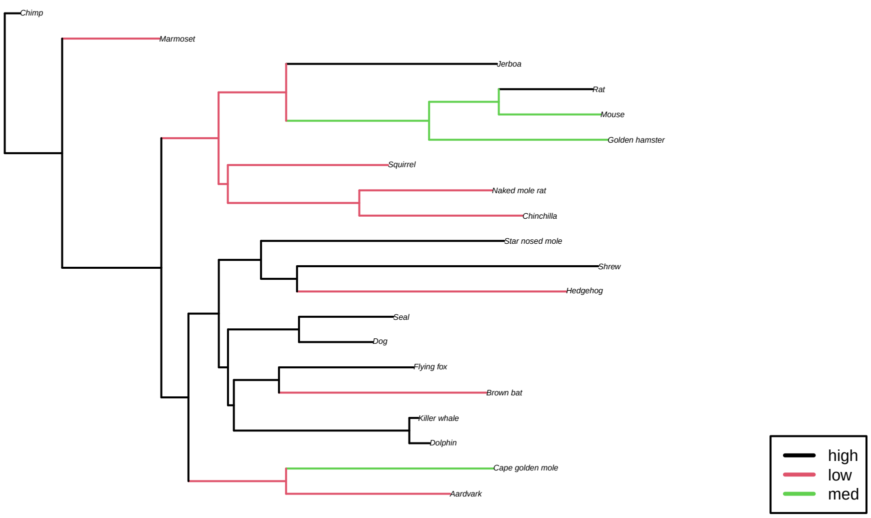


Next calculate the association statistics relating the relative evolutionary rates to basal metabolic rate phenotype. For more details on how the output of `correlateWithCategoricalPhenotype` is organized, refer to the Categorical Walkthrough.

```
# Kruskal Wallis/Dunn posthoc testing (default)
cors = correlateWithCategoricalPhenotype(RERmat, charP)

# view the first few results
head(cors[[1]][order(cors[[1]]$P),])
```

```
##             Rho  N           P     p.adj
## ADAD1 0.3229020 35 0.004130593 0.2876621
## ARSA  0.3764943 29 0.005138883 0.2876621
## AP5M1 0.3056010 35 0.005543016 0.2876621
## BRAF  0.3700350 28 0.006768521 0.2876621
## BRSK2 0.3024616 31 0.010706289 0.2907447
## BDH1  0.2717130 34 0.011296477 0.2907447
```

## Performing Permulations

The goal of running the permulation analysis is to generate many permulated phenotypes then calculate correlation statistics relating evolutionary rates of genes to the trait for each permulated phenotype. This generates a set of null correlation statistics – the statistics we would expect by chance given the same phylogeny and same numbers of species with each trait value. Permulation p-values are thus the fraction of correlation statistics among the permulated phenotypes that are more extreme than the correlation statistic calculated for the original trait data.

### Generate Permulated Phenotypes

**Standard Approach** To run a permulation analysis, start by generating a set of permulated phenotypes using the function `categoricalPermulations`. In this example we generate 100 permulated phenotypes. For a more rigorous analysis we recommend using 1000 or more permulations (though this may be time consuming). This is because the permulation p-values can only be as precise as one over the number of permulations performed. `categoricalPermulations` takes the following as input:

- `treesObj` : The trees object containing every gene tree returned by `readTrees`

- `phenvals` : The named vector of phenotype data (should be a categorical phenotype)
- `rm` : The rate model. **This should be the same rate model as passed to `char2PathsCategorical` to perform the ancestral reconstruction.**
- `rp` : The root probabilities to use when simulating the phenotype. This gives the probability of each state at the root. The default value is `"auto"`. It can also be a numeric vector with length equal to the number of phenotype categories. Other options are `"stationary"` and `"flat"`. `"flat"` sets the probabilities of all categories at the root equal. `"stationary"` uses the stationary distribution of the transition matrix. For more details refer to the documentation for the castor function `simulate_mk_model` (Louca and Doebeli 2017).
- `ntrees` : the number of permulated phenotypes to generate

The following code generates 100 permulated phenotypes. This step may take a few minutes.

```
perms <- categoricalPermulations(toyTrees, phenvals = basalRate, rm = "ARD",
                                 rp = "auto", ntrees = 100)
```

```
## Fitting transition matrix

## Simulating trees

## Shuffling internal states

## Improving tree likelihoods

## Done
```

`perms`, the output of `categoricalPermulations` is a 3-element list. The first element `sims` contains the original simulated trees. `sims` is a list of two matrices, `tips` and `nodes`. The matrices have `ntrees` rows corresponding to the `ntrees` simulations. Columns of the `tips` and `nodes` matrices correspond to the extant or internal species respectively. The second element is `trees`. These are the permulated phenotypes. `trees` is an `ntrees`-element list. Each element of `trees` is itself a list containing a `tips` vector and a `nodes` vector corresponding to the states of the extant and internal species. The third element of `perms` is `startingTrees`. `startingTrees` has the same structure as `trees` and corresponds to the permulated phenotypes before step 3, re-organize internal traits (see Categorical Permulations Overview).

**Relaxed Approach** The `categoricalPermulations` function in fact takes another optional parameter, `percent_relax`, which by default is set to zero. This argument defines the percentage of the original category counts by which the permulated phenotype may differ. It can either be a single percentage value or a vector of percentage values - one for each category, in the same order as the integer labels used by `char2TreeCategorical` and `char2PathsCategorical`. For phenotypes with a large number of categories, using relaxation may be required to get permulations to run in a tractable amount of time. A small relaxation, of around 10%, has been shown to work for phenotypes with up to 6 categories without noticeably impacting the quality of the results (Redlich et al. 2023). The following code can be used to generate permulations with relaxation. (Shown with a relaxation of 10%).

```
relaxedPerms <- categoricalPermulations(toyTrees, phenvals = basalRate, rm = "ARD",
                                 rp = "auto", ntrees = 100, percent_relax = 10)
```

```
## Fitting transition matrix
```

```
## Simulating trees

## Shuffling internal states

## Improving tree likelihoods

## Done
```

The output is in the same format as when there is no relaxation applied, and all subsequent steps are identical.

### Visualize Permulated Phenotypes

We can easily visualize some of the permulated phenotypes. For convenience we define a function that will plot the states as colored circles on the tree. Note that your trees will look slightly different from the ones shown here.

```
# define a function to plot the permulated phenotypes on the tree
library(RERconverge)
```

```
## Warning: package 'phytools' was built under R version 4.2.3

## Warning: package 'maps' was built under R version 4.2.3

## Warning: package 'RcppArmadillo' was built under R version 4.2.3

## Warning: package 'dplyr' was built under R version 4.2.3

## Warning: package 'Matrix' was built under R version 4.2.3
```

```
plotPermPhen <- function(tree, tips, internal_states) {
  plot(tree, label.offset = 0.005, cex = 0.5)
  tiplabels(pie = to.matrix(tips, sort(unique(tips))),cex = 0.5)
  nodelabels(pie = to.matrix(internal_states, sort(unique(internal_states))), cex = 0.5)
}

# prune the master tree to only contain species for which there are phenotype values
tree = toyTrees$masterTree
tree = pruneTree(tree, names(basalRate))

# plot some of the permulated trees
plotPermPhen(tree, perms$trees[[1]]$tips, perms$trees[[1]]$nodes)
```

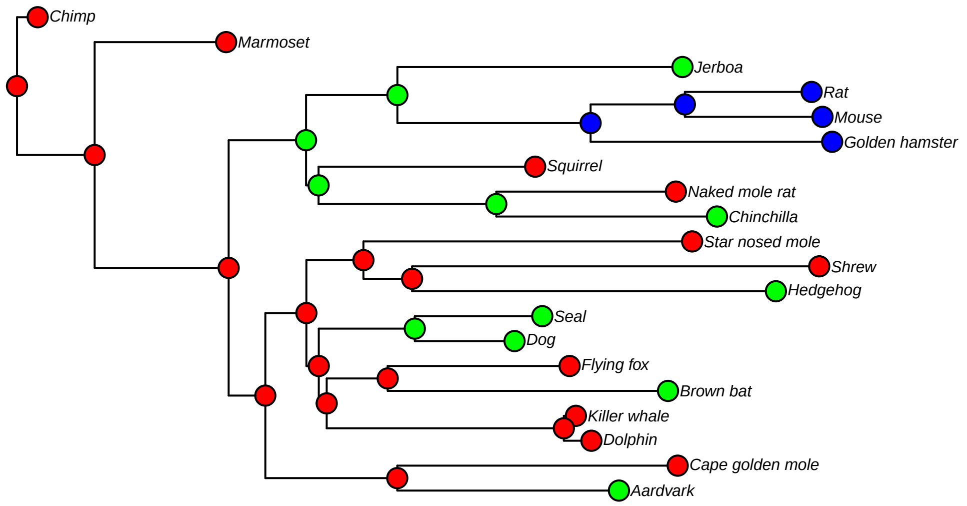


```
plotPermPhen(tree, perms$trees[[25]]$tips, perms$trees[[25]]$nodes)
```

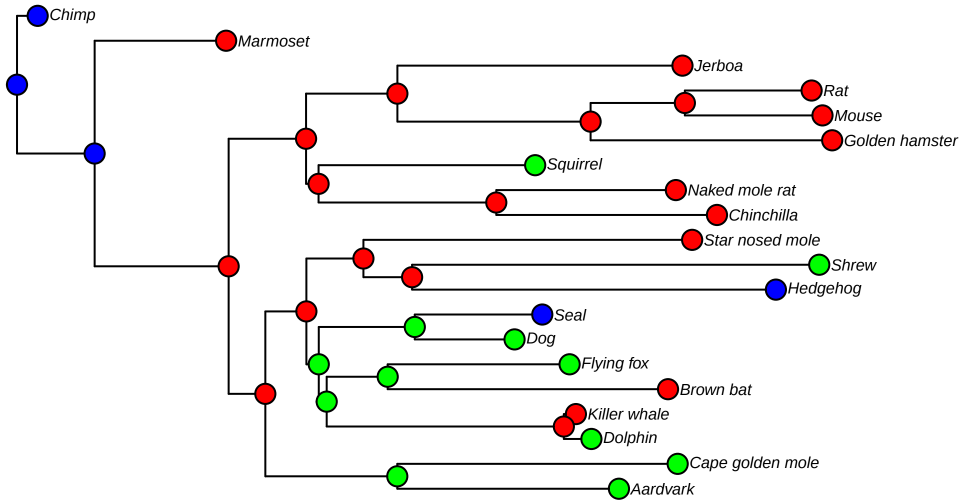


```
plotPermPhen(tree, perms$trees[[50]]$tips, perms$trees[[50]]$nodes)
```

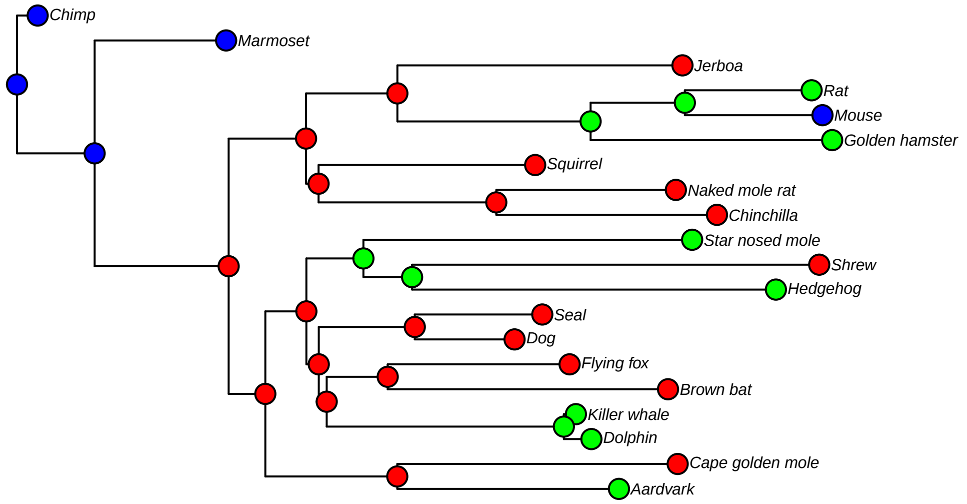

```
plotPermPhen(tree, perms$trees[[75]]$tips, perms$trees[[75]]$nodes)
```

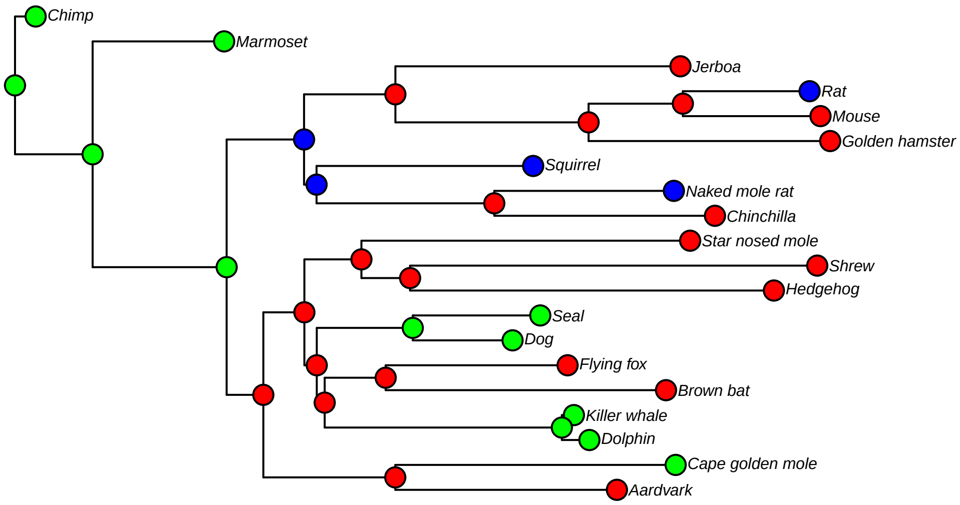


```
plotPermPhen(tree, perms$trees[[100]]$tips, perms$trees[[100]]$nodes)
```

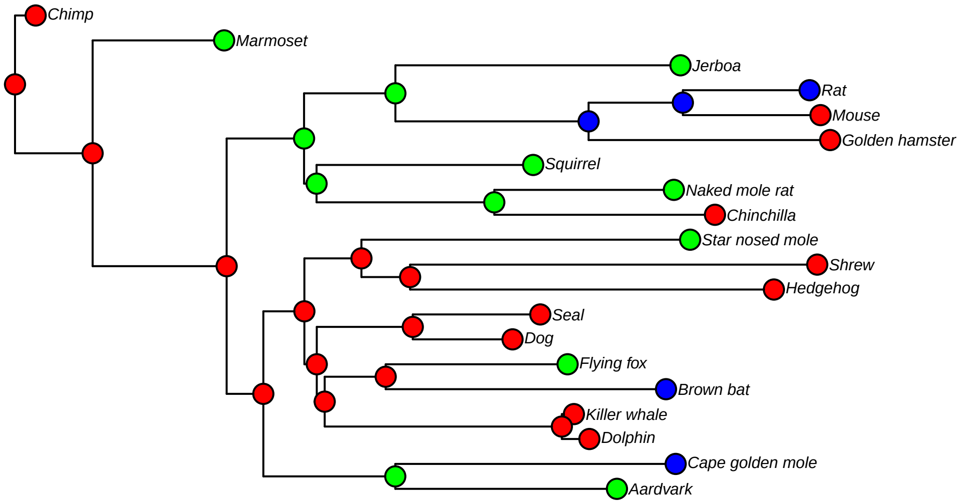


### Obtain Permulation P-values

We can obtain permulation p-values using the function `getPermPvalsCategorical`. This function takes as input:

- `realCors` : the correlation statistics object returned by `correlateWithCategoricalPhenotype`.
- `nullPhens` : the permulated phenotypes. This should be the `trees` element in the list returned by `categoricalPermulations`.
- `phenvals` : The named phenotype vector.
- `treesObj` : The trees object returned by `readTrees`.
- `RERmat` : The matrix of relative evolutionary rates returned by `getAllResiduals`.

- `method` : either `"kw"` for Kruskal Wallis or `"aov"` for ANOVA; this should be whichever method was used to calculate the correlation statistics using `correlateWithCategoricalPhenotype`, the default method of which is `"kw"`. If another method is provided that is not `"kw"` or `"aov"`, then the trait will be treated as a binary trait.

```
pres <- getPermPvalsCategorical(realCors = cors, nullPhens = perms$trees,
                               phenvals = basalRate, treesObj = toyTrees,
                               RERmat = RERmat, method = "kw")
```

```
## Generating null paths

## Calculating correlation statistics

## Obtaining permulations p-values

## Done
```

The output of `getPermPvalsCategorical` is a 3-element list. The first element, `res`, has the same format as `cors`, the output of `correlateWithCategoricalPhenotype`, except each data frame has an additional column called `permP` containing the permulation p-values. The second and third elements are `pvals` and `effsize`. Each of these is a 2-element list. The first element contains a (number of genes) x (number of permulations) table containing the p-value or effect size of each gene for each permulation. The second element contains a list of tables for each pairwise test. Each table has dimensions (number of genes) x (number of permulations) and contains the p-value or effect size of each gene for each permulation.

If the trait is binary (has only 2 categories) then the output will look very similar except it will not contain lists of tables for the pairwise tests.

The pairwise tables are named with the integer mappings to the categories e.g. `"1 - 2"`. Recall that the integer mapping is printed by `char2PathsCategorical` and can also be obtained by running the following code:

```
# view the names of the pairwise tables
names(pres$res[[2]])
```

```
## [1] "1 - 2" "1 - 3" "2 - 3"
```

```
# get the category to integer mapping
intlabels = map_to_state_space(basalRate)
print(intlabels$name2index)
```

```
## high  low  med
##    1    2    3
```

```
# view the results ordered by permulation p-value
head(pres$res[[1]][order(pres$res[[1]]$permP),])
```

```
##                     Rho  N           P     p.adj permP
## ADAD1         0.3229020 35 0.004130593 0.2876621  0.00
## AP5M1         0.3056010 35 0.005543016 0.2876621  0.00
## ARSA          0.3764943 29 0.005138883 0.2876621  0.00
## BIRC5         0.3047823 27 0.019021805 0.3216999  0.01
## BRAF          0.3700350 28 0.006768521 0.2876621  0.01
## Em:AC008101.5 0.1797311 33 0.056376766 0.3986397  0.02
```

```r
# view the results of the pairwise tests ordered by permulation p-value
head(pres$res[[2]][[1]][order(pres$res[[2]][[1]]$permP),]) # high - low
```

```
##              Rho          P p.adj permP
## ARSA   -2.708968 0.02024788     1  0.00
## BIRC5   1.775278 0.22755608     1  0.01
## BTBD18 -2.502992 0.03694448     1  0.01
## APOH    2.144787 0.09590962     1  0.03
## BRAF   -2.389181 0.05065792     1  0.03
## ABHD5  -1.859582 0.18883420     1  0.04
```

```r
head(pres$res[[2]][[2]][order(pres$res[[2]][[2]]$permP),]) # high - med
```

```
##                Rho          P     p.adj      permP
## ACOT13    2.658824 0.02352420 0.7058718 0.00000000
## ADAM1A    2.498884 0.03737552 0.7802140 0.01000000
## ANO2      2.927877 0.01023855 0.7058718 0.01000000
## ADM      -2.376555 0.05242547 0.8348500 0.01000000
## BRAF     -2.726717 0.01919035 0.7058718 0.01000000
## BC118554 -1.767767 0.23129962 1.0000000 0.01388889
```

```r
head(pres$res[[2]][[3]][order(pres$res[[2]][[3]]$permP),]) # low - med
```

```
##                     Rho           P     p.adj permP
## Em:AC008101.5  2.397934 0.049463461 0.6740709  0.00
## ADAD1          3.281030 0.003102857 0.3328440  0.00
## AP5M1         -3.209704 0.003986156 0.3328440  0.00
## ARSA           2.691795 0.021320581 0.4705343  0.00
## BIRC5         -2.682493 0.021922723 0.4705343  0.00
## ACTL7B         2.710343 0.020164073 0.4705343  0.02
```

## Categorical Permulations for Pathway Enrichment Statistics

For details on how pathway enrichment statistics are calculated, refer to the RERconverge Analysis Walkthrough vignette. Essentially a pathway enrichment analysis identifies groups of genes that are evolving faster or slower with the phenotype of interest. We recommend calculating permulation p-values for the pathway enrichment statistics in addition to the gene-evolutionary rate association statistics due to non-independence between genes in pathways.

### Getting Started with Pathway Enrichment

You will need to download the gene sets and gene symbols from GSEA-MSigDB as gmtfile.gmt. Follow the instructions in the "RERconverge Analysis Walkthrough" vignette in order to properly download and save the gmt file in your current working directory. The "RERconverge Analysis Walkthrough" may say to download the file named c2.all.v6.2.symbols.gmt, however if that is not available, c2.all.v7.5.1.symbols.gmt will work. Ensure that the name of the gmt file in your working directory is "gmtfile.gmt".

```r
# read in the annotations
annots = read.gmt("gmtfile.gmt")

# format in a list
annotlist=list(annots)
names(annotlist)="MSigDBpathways"
```

#### Obtain Permulation P-values for Pathway Enrichment Statistics

**Calculate Enrichment Statistics for the Original Gene Association Results** The first step is the calculate the pathway enrichment statistics for the original gene association results before permulations (the output of `correlateWithCategoricalPhenotype`). This can be done using the function `getRealEnrichments` which calls the RERconverge function, `fastwilcoxGMTall`, to calculate enrichment statistics for the categorical results and the results of each posthoc pairwise test.

Note that running `getRealEnrichments` can be time consuming especially if the number of pairwise tests is large.

`getRealEnrichments` takes the following as input:

- `cors` : the output of `correlateWithCategoricalPhenotype`
- `annotlist` : the pathway annotations formatted as a list
- `outputGeneVals` : the default value is `FALSE`. If set to `TRUE`, the genes in each pathway will be included in the output.

```r
# run enrichments
realenrich <- getRealEnrichments(cors, annotlist)
```

The output of `getRealEnrichments` is a 2-element list. The first element contains the enrichment statistics for the categorical correlations results. The second element contains a list of enrichment statistics for each posthoc pairwise test. For more information on interpreting pathway enrichment results, refer to the Enrichment Walkthrough section in the RERconverge Analysis Walkthrough vignette.

**Calculate Permulation P-values** Recall that `getPermPvalsCategorical` returns a list of p-value matrices and effect size matrices. Each column in these matrices corresponds to the parametric p-values or effect size statistics returned by `correlateWithCategoricalPhenotype` (or `getAllCors`) for one permulated phenotype. To calculate permulation p-values for the enrichment statistics, null enrichment statistics are calculated for each permulated phenotype using a ranked gene list based on the p-values and effect size statistics for that permulated phenotype. This is handled by the functions, `getEnrichPermsCategorical`. Then the permulation p-value is determined by the proportion of times the null enrichment statistics are more extreme than the real enrichment statistics returned by `getRealEnrichments`. This is handled by the function `getEnrichPermPvals`.

During a call to `getEnrichPermsCategorical`, `fastwilcoxGMTall` (the RERconverge function that calculates enrichmentis statistics) is called many times (once per permulation for the categorical results and once per permulations for EACH pairwise test). As a result `getEnrichPermsCategorical` can take a long time to run.

`getEnrichPermsCategorical` takes the following as input:

- `perms`: The output of `getPermPvalsCategorical`; the object containing the null p-values and null enrichment statistics for each permulated phenotype.

- `realenrich`: The output of `getRealEnrichments`; the pathway enrichment statistics on the original gene association results.
- `annotlist`: the list of pathway annotations formatted as a list as shown above

```
# run enrichments permulations
permenrich = getEnrichPermsCategorical(perms = pres, realenrich = realenrich,
                                       annotlist = annotlist)
```

`permenrich`, the output of `getEnrichPermsCategorical`, is a 2-element list. The first element contains a list of tables of P-values and a list of tables of enrichment statistics. There is one table of p-values or enrichment statistics for each annotation pathway set. For the annotation list in this walkthrough these sets are: mgi, canonical, GO, hairfollicle, and tissueannots. The second element contains a list of such lists, one for each posthoc pairwise test.

To calculate permulation p-values, call the function `getEnrichPermPvals` which takes the following as input:

- `permenrich`: the output of `getEnrichPermsCategorical` containing the enrichment statistics and p-values for each permulated phenotype
- `realenrich`: the output of `getRealEnrichments` containing the enrichment statistics and p-values for the original gene association results for the original phenotype.

```
pvals = getEnrichPermPvals(permenrich, realenrich)
```

The output of `getEnrichPermPvals` is also a 2-element list of very similar format to the output of of `getEnrichPermsCategorical` except that instead of tables of p-values and enrichment statistics, there is a list of named numeric vectors of permulation p-values for each pathway in each annotation pathway set.

The code below demonstrates how to view the permulation p-values for the enrichment pathways.

```
# convert the mgi annotations ordered by p-value to a dataframe
df = as.data.frame(pvals[[1]]$MSigDBpathways[order(pvals[[1]]$MSigDBpathways)])
colnames(df) = c("permulation p-values")
head(df)
```

```
##                                                        permulation p-values
## FLECHNER_BIOPSY_KIDNEY_TRANSPLANT_REJECTED_VS_OK_DN               0.0200000
## REACTOME_TRANSPORT_OF_SMALL_MOLECULES                             0.1000000
## REACTOME_METABOLISM_OF_LIPIDS                                     0.1300000
## BRUINS_UVC_RESPONSE_VIA_TP53_GROUP_A                              0.1600000
## REACTOME_INNATE_IMMUNE_SYSTEM                                     0.1600000
## DODD_NASOPHARYNGEAL_CARCINOMA_DN                                  0.1702128
```

```
# do the same for the first posthoc pairwise test
# change the number 1 in the second set of brackets (to 2 or 3) to view the other posthoc tests
df = as.data.frame(pvals[[2]][[1]]$MSigDBpathways[order(pvals[[2]][[1]]$MSigDBpathways)])
colnames(df) = c("permulation p-values")
head(df)
```

```
##                                             permulation p-values
## GOBERT_OLIGODENDROCYTE_DIFFERENTIATION_DN             0.06000000
## ZWANG_TRANSIENTLY_UP_BY_2ND_EGF_PULSE_ONLY            0.07000000
```

```
## MIKKELSEN_ES_ICP_WITH_H3K4ME3                          0.08695652
## NUYTTEN_EZH2_TARGETS_DN                                0.14583333
## CHEN_METABOLIC_SYNDROM_NETWORK                         0.18000000
## BRUINS_UVC_RESPONSE_VIA_TP53_GROUP_A                   0.36000000
```

We are often interested not only in the permulation p-values of the pathways, but the direction and magnitude of the association given by the enrichment statistic. The following code demonstrates how to add the permulation p-values to the original enrichment results.

```
# make a copy of the real enrichment results
enrichWithPvals = realenrich

# add p-values for each annotation set in the first element of enrichWithPvals
for(cnt in 1:length(enrichWithPvals[[1]])) {
  indices = match(rownames(enrichWithPvals[[1]][[cnt]]), names(pvals[[1]][[cnt]]))
  enrichWithPvals[[1]][[cnt]]$permpvals = pvals[[1]][[cnt]][indices]
}

# add p-values for each annotation set in the second element of enrichWithPvals
# (the list of posthoc pairwise tests)
for(j in 1:length(enrichWithPvals[[2]])){
  name = names(enrichWithPvals[[2]])[j] # the name of the pairwise test
  for(cnt in 1:length(enrichWithPvals[[2]][[j]])){
    indices = match(rownames(enrichWithPvals[[2]][[j]][[cnt]]),
                    names(pvals[[2]][[name]][[cnt]]))
    enrichWithPvals[[2]][[j]][[cnt]]$permpvals = pvals[[2]][[name]][[cnt]][indices]
  }
}

# view some of the results
head(enrichWithPvals[[1]]$MSigDBpathways[order(enrichWithPvals[[1]]$MSigDBpathways$permpvals),])
```

```
##                                                    stat       pval
## FLECHNER_BIOPSY_KIDNEY_TRANSPLANT_REJECTED_VS_OK_DN 0.14572193 0.05425690
## REACTOME_TRANSPORT_OF_SMALL_MOLECULES               0.15520362 0.01887403
## REACTOME_METABOLISM_OF_LIPIDS                       0.09898990 0.10487603
## BRUINS_UVC_RESPONSE_VIA_TP53_GROUP_A                0.07962963 0.18072012
## REACTOME_INNATE_IMMUNE_SYSTEM                       0.03141711 0.36466665
## DODD_NASOPHARYNGEAL_CARCINOMA_DN                    0.08540146 0.18403651
##                                                         p.adj num.genes
## FLECHNER_BIOPSY_KIDNEY_TRANSPLANT_REJECTED_VS_OK_DN 0.6572732        11
## REACTOME_TRANSPORT_OF_SMALL_MOLECULES               0.4718508        17
## REACTOME_METABOLISM_OF_LIPIDS                       0.6572732        15
## BRUINS_UVC_RESPONSE_VIA_TP53_GROUP_A                0.6572732        12
## REACTOME_INNATE_IMMUNE_SYSTEM                       0.8673555        11
## DODD_NASOPHARYNGEAL_CARCINOMA_DN                    0.6572732        10
##                                                     gene.vals permpvals
## FLECHNER_BIOPSY_KIDNEY_TRANSPLANT_REJECTED_VS_OK_DN        NA 0.0200000
## REACTOME_TRANSPORT_OF_SMALL_MOLECULES                      NA 0.1000000
## REACTOME_METABOLISM_OF_LIPIDS                              NA 0.1300000
## BRUINS_UVC_RESPONSE_VIA_TP53_GROUP_A                       NA 0.1600000
## REACTOME_INNATE_IMMUNE_SYSTEM                              NA 0.1600000
## DODD_NASOPHARYNGEAL_CARCINOMA_DN                           NA 0.1702128
```

```r
# view some of the results for the first pairwise test
head(enrichWithPvals[[2]][[1]]$MSigDBpathways[order(enrichWithPvals[[2]][[1]]$MSigDBpathways$permpvals)
```

```
##                                                   stat      pval     p.adj
## GOBERT_OLIGODENDROCYTE_DIFFERENTIATION_DN   0.13502674 0.1369837 0.9386787
## ZWANG_TRANSIENTLY_UP_BY_2ND_EGF_PULSE_ONLY -0.11600430 0.1540540 0.9386787
## MIKKELSEN_ES_ICP_WITH_H3K4ME3               0.15364964 0.1053633 0.9386787
## NUYTTEN_EZH2_TARGETS_DN                     0.11094891 0.2422642 0.9386787
## CHEN_METABOLIC_SYNDROM_NETWORK              0.08799342 0.2165926 0.9386787
## BRUINS_UVC_RESPONSE_VIA_TP53_GROUP_A        0.09444444 0.2790683 0.9386787
##                                            num.genes gene.vals  permpvals
## GOBERT_OLIGODENDROCYTE_DIFFERENTIATION_DN         11        NA 0.06000000
## ZWANG_TRANSIENTLY_UP_BY_2ND_EGF_PULSE_ONLY        14        NA 0.07000000
## MIKKELSEN_ES_ICP_WITH_H3K4ME3                     10        NA 0.08695652
## NUYTTEN_EZH2_TARGETS_DN                           10        NA 0.14583333
## CHEN_METABOLIC_SYNDROM_NETWORK                    19        NA 0.18000000
## BRUINS_UVC_RESPONSE_VIA_TP53_GROUP_A              12        NA 0.36000000
```

# Conclusion

This concludes the walkthrough of how to use the functions for permulations for categorical traits in RERconverge. Thank you!


Louca, Stilianos, and Michael Doebeli. 2017. "Efficient Comparative Phylogenetics on Large Trees." https://doi.org/10.1093/bioinformatics/btx701.

Redlich, Ruby, Amanda Kowalczyk, Michael Tene, Heather H. Sestili, Kathleen Foley, Elysia Saputra, Nathan Clark, Maria Chikina, Wynn K. Meyer, and Andreas Pfenning. 2023. "RERconverge Expansion: Using Relative Evolutionary Rates to Study Complex Categorical Trait Evolution." https://doi.org/10.1101/2023.12.06.570425.

# Calculation of Association Statistics from Extant Species Only

December 04, 2022

## Contents

**Overview** **2**
Data and Input Requirements . . . . . 2

**Analysis Walkthrough** **2**
Reading in Trees and Calculating Relative Evolutionary Rates . . . . . 2
Binary Traits . . . . . 2
Continuous Traits . . . . . 3
Categorical Traits . . . . . 4

**Conclusion** **5**

This walkthrough demonstrates how to perform an RERconverge analysis using only the data at the tips of the tree, skipping the phylogenetic inference step of a typical RERconverge analysis. This walkthrough builds on existing RERconverge objects. First time users should first read the "RERconverge Analysis Walkthrough" vignette for information on installation, setup, and getting started.

# Overview

Typically, we recommend including ancestral states in an RERconverge analysis because incorporating evolutionary information can strengthen the statistical power of the analysis. However, there may be phenotypes in which ancestral states are not as informative and could add noise to the results. In that case, we present a method for calculating association statistics between relative evolutionary rates and phenotype values using only the extant species in the tree.

## Data and Input Requirements

The required inputs are as follows:

1. Phylogenetic trees of the same format described in the "RERconverge Analysis Walkthrough" vignette.
2. Species-labeled phenotype values

- The species labels MUST match the tree tip labels that will be used in `getAllResiduals` to calculate the relative evolutionary rates (RERs)
- a named vector of binary, continuous, or categorical trait values

# Analysis Walkthrough

## Reading in Trees and Calculating Relative Evolutionary Rates

Refer to the "RERconverge Analysis Walkthrough" vignette to learn how to read in gene trees using `readTrees` and calculate evolutionary rates using `getAllResiduals`.

Running the code below will read in some example trees that come with the RERconverge package that we will use for this walkthrough.

```
# check RERconverge is properly installed
library(RERconverge)

# find where the package is located on your machine
rerpath = find.package('RERconverge')

# read in the trees with the given file name
toytreefile = "subsetMammalGeneTrees.txt"
toyTrees=readTrees(paste(rerpath,"/extdata/",toytreefile,sep=""), max.read = 200)

# calculate the relative evolutionary rates with getAllResiduals
RERmat = getAllResiduals(toyTrees)
```

## Binary Traits

First, we define foreground species for the hibernation binary phenotype and generate a named phenotype vector.

```
library(RERconverge)
# define the foreground species
hibextantforeground = c("Vole", "Brown_bat", "Myotis_bat", "Squirrel", "Jerboa")
```

```r
# make a phenotype vector for the species in the tree
# the phenotype values must be numeric (0 and 1 instead of TRUE and FALSE)
hibphenvals = rep(0, length(toyTrees$masterTree$tip.label))
names(hibphenvals) = toyTrees$masterTree$tip.label
# set the foreground species to true
hibphenvals[hibextantforeground] = 1
```

Finally, we calculate statistics using `getAllCorExtantOnly` which takes the following as input:

- `RERmat`: The RER matrix returned by `getAllResiduals`.
- `phenvals`: the named phenotype vector with names matching those used to calculated RERs in `getAllResiduals`.
- `method`: set to `"k"` for binary traits to calculate Kendall rank coefficients, `"p"` for continuous traits to use a Pearson correlation, and `"aov"` or `"kw"` for categorical traits to use an ANOVA or Kruskal Wallis test respectively.
- `min.sp`: The minimum number extant species in the gene tree for that gene to be included in the analysis.
- `min.pos`: The minimum number of extant foreground species in the gene tree for that gene to be included in the analysis.
- `winsorizeRER/winsorizetrait`: pulls the most extreme N values (default N=3) in both the positive and negative tails to the value of the N+1 most extreme value. This process mitigates the effect of extreme outliers before calculating correlations.

```r
# set method to k to use a Kendall rank test since this is a binary phenotype
cors = getAllCorExtantOnly(RERmat, hibphenvals, method = "k")

# view the top results
head(cors[order(cors$P),])
```

```
##                        Rho  N           P     p.adj
## AGTRAP           0.3309892 48 0.005948218 0.3315598
## AL833346         0.3601512 39 0.007255622 0.3315598
## Em:AC008101.5    0.3332454 42 0.009799975 0.3315598
## ACOXL            0.2811551 58 0.009948652 0.3315598
## ASIC3           -0.2701134 57 0.014120008 0.3315598
## AMMECR1L         0.3573159 30 0.020430973 0.3315598
```

For further analysis of the gene results, such as calculating functional enrichments, refer to the "RERconverge Analysis Walkthrough" vignette.

## Continuous Traits

We will follow much the same steps for continuous traits as we did for binary traits. First, ensure that you followed the instructions above for reading in the gene trees and calculating relative evolutionary rates.

Next, we will load in some example data provided by RERconverge for the mammal body weight phenotype and calculate association statistics.

```r
# load in the example data
data("logAdultWeightcm")

# set method to p to use a Pearson correlation since this is a continuous phenotype
cors = getAllCorExtantOnly(RERmat, phenvals = logAdultWeightcm, method = "p")
```

```r
# view the top results
head(cors[order(cors$P),])
```

```
##                 Rho  N            P       p.adj
## DNAH7    -0.5349841 59 1.269896e-05 0.002488997
## ADAMTSL4 -0.4827883 59 1.076584e-04 0.010550520
## DNAH6    -0.4010073 61 1.361267e-03 0.058734961
## AL833346 -0.4962212 38 1.532360e-03 0.058734961
## ATP2A1    0.4189829 54 1.614352e-03 0.058734961
## ADH7      0.4574178 44 1.798009e-03 0.058734961
```

For further analysis of the gene results, such as calculating functional enrichments, refer to the "RERconverge Analysis Walkthrough" vignette.

## Categorical Traits

Once again, we will follow very similar steps for categorical traits as for both binary and continuous traits. First, ensure that you followed the instructions above for reading in the gene trees and calculating relative evolutionary rates.

Next, we will load in some example data provided by RERconverge for the basal rate phenotype and calculate association statistics.

```r
# load in the example data
data("basalRate")

# set method to kw to use a Kruskal Wallis test since this is a categorical phenotype
cors = getAllCorExtantOnly(RERmat, phenvals = basalRate, method = "kw")
```

Finally, we can view the results for all categories or for the pairwise comparisons between categories.

```r
# the first element of cors is a table of association statistics for the Kruskal Wallis
# or ANOVA test across categories
all_categories_results = cors[[1]]
# view top results
head(all_categories_results[order(all_categories_results$P),])
```

```
##               Rho  N          P p.adj
## ABLIM2   7.373950 16 0.02504766     1
## AK124326 6.679487 12 0.03544605     1
## AP5M1    6.402005 19 0.04072136     1
## ADAD1    6.240000 19 0.04415717     1
## ACRV1    5.798496 19 0.05506461     1
## BTBD18   5.726316 19 0.05708820     1
```

```r
# the second element of cors is a list of tables of pairwise comparisons between categories
pairwise_tests = cors[[2]]
names(pairwise_tests)
```

```
## [1] "high - low" "high - med" "low - med"
```

```r
# view top results of pairwise test between low and medium basal rate species
head(pairwise_tests[[3]][order(pairwise_tests[[3]]$P),])
```

```
##                Rho          P p.adj
## AP5M1    -2.464820 0.04112461     1
## ADAD1     2.387467 0.05089474     1
## ATP6V1G2 -2.325377 0.06015543     1
```

```
## ACRV1      2.293141 0.06551964     1
## BBOX1      2.210365 0.08123948     1
## APOH      -2.207302 0.08187898     1
```

## Conclusion

This concludes the walkthrough on how to calculate association statistics between phenotype and relative evolutionary rates with only the extant species in the tree.
